# Labeling matters: A multicenter machine learning study on visual field progression in Glaucoma

**DOI:** 10.1371/journal.pone.0357179

**Published:** 2026-08-28

**Authors:** Hyobeen Kim, EunAh Kim, Sangwoo Moon, Sang Wook Jin, Jung Lim Kim, Seung Uk Lee, Jeong Rye Park, Jiwoong Lee

**Affiliations:** 1 Department of Mathematics, Chonnam National University, Gwangju, Korea; 2 Department of Ophthalmology, Samsung Changwon Hospital, Sungkyunkwan University School of Medicine, Changwon, Korea; 3 Department of Ophthalmology, Pusan National University Yangsan Hospital, Pusan National University College of Medicine, Yangsan, Korea; 4 Department of Ophthalmology, Dong-A University College of Medicine, Busan, Korea; 5 Department of Ophthalmology, Busan Paik Hospital, Inje University College of Medicine, Busan, Korea; 6 Department of Ophthalmology, Kosin University College of Medicine, Busan, Korea; 7 Department of Mathematical Sciences, Gwangju Institute of Science and Technology, Gwangju, Korea; 8 Department of Ophthalmology, Pusan National University College of Medicine, Busan, Korea; 9 Biomedical Research Institute, Pusan National University Hospital, Busan, Korea; Daegu Veterans Health Service Medical Center, KOREA, REPUBLIC OF

## Abstract

**Background:**

To compare machine learning (ML) performance for detecting visual field (VF) progression across different labeling strategies using a large multicenter dataset.

**Methods:**

In this multicenter retrospective study, VF data were collected from five tertiary referral hospitals. Two algorithm-derived labeling approaches were evaluated without an independent clinical reference standard: an inclusive Consensus label, defined as progression detected by at least one of five conventional algorithms (mean deviation slope, Visual Field Index slope, Advanced Glaucoma Intervention Study, Collaborative Initial Glaucoma Treatment Study, and pointwise linear regression), and a conservative Wiggs’ label, based on a region-based event–threshold rule. Four ML classifiers, support vector machine, random forest, logistic regression, and extreme gradient boosting, were trained using each labeling strategy. Model performance was assessed using the area under the receiver operating characteristic curve (AUC), sensitivity, specificity, and precision–recall analysis summarized by average precision (AP).

**Results:**

Using the Consensus label, all models demonstrated excellent discrimination (AUC, 0.92–0.95), with high sensitivity (0.82–0.85) and near-perfect specificity (0.99–1.00). Precision–recall analysis showed consistently high reliability of progression detection, with AP values ranging from 0.93 to 0.94. In contrast, models trained with the Wiggs’ label exhibited lower AUCs (0.88–0.89) and reduced sensitivity (0.63–0.72), while maintaining moderate-to-high specificity (0.87–0.92) and lower AP values (0.84–0.85), reflecting a stricter, region-based progression definition. Ablation analysis showed that Consensus-based performance was not driven by any single criterion, but rather by complementary information across heterogeneous progression algorithms.

**Conclusion:**

In this multicenter study, the labeling strategy was a major determinant of ML performance in VF progression detection. The Consensus label enabled sensitive and reliable identification of progression with high specificity with respect to the Consensus label definition across heterogeneous clinical settings, whereas the Wiggs’ label provided conservative, spatially consistent confirmation. The observed performance differences primarily reflect model-label compatibility rather than the clinical validity of either detection system, underscoring that careful definition of ground truth is critical for interpreting ML-based glaucoma progression research.

## Introduction

Accurate and consistent determination of progressive visual field (VF) loss is a key challenge in glaucoma management. VF testing is inherently subject to test–retest variability, which has prompted the development of various statistical approaches to detect progression [1–3]. Among these, event-based methods (guided progression analysis, Advanced Glaucoma Intervention Study [AGIS], and Collaborative Initial Glaucoma Treatment Study [CIGTS] criteria) and trend-based methods (mean deviation [MD] slope, Visual Field Index [VFI] slope, pointwise linear regression [PLR], and permutation of PLR [PoPLR]) are widely used [4–6]. However, agreement among these progression algorithms is low (κ = 0.12–0.52), leading to inconsistent classification of progression and uncertainty in clinical decision-making [5,7–9]. Furthermore, relying solely on any single algorithm carries an inherent risk of false-negative classification, as each method may miss progression signals that others detect [10]. This variability poses a fundamental challenge when constructing labeled datasets for supervised machine learning, as the choice of labeling strategy directly determines what the model learns to detect.

To address this challenge, we proposed a “Consensus label” defined as positive when at least one of the five algorithms detected progression. This “any-positive” strategy was adopted based on both clinical and methodological rationale. Glaucoma is the leading cause of irreversible blindness worldwide, and because visual loss once incurred cannot be restored, early detection and timely intervention are paramount [11]. Given this irreversibility, the clinical consequence of a missed progression event (false negative) far outweighs the cost of a false-positive alert. Since each algorithm may detect progression signals that others miss, particularly given that different methods are sensitive to different patterns of VF loss, requiring agreement among multiple algorithms before assigning a positive label risks systematically discarding genuine progression signals present in the training data. The any-positive Consensus label was therefore designed to maximize sensitivity in label assignment, ensuring that eyes with any algorithm-detectable signal of deterioration are retained as positive cases, consistent with the clinical principle that any indicator of progression in an irreversible disease warrants heightened vigilance.

Previous studies have proposed adopting a consensus-based ground-truth to address inconsistency among multiple progression algorithms [4,6]. Sabharwal et al. [6] defined progression when at least four out of six criteria agreed and developed a deep learning model that integrated spatial and temporal information based on this consensus labeling. Recent studies across medical AI applications have shown that outcome definition and labeling strategy can substantially influence model performance and generalizability, often exceeding the impact of model architecture itself [12–14]. We therefore hypothesized that, in glaucoma progression modeling, the definition of VF progression used for labeling would substantially influence machine learning performance, independent of the underlying model. However, to our knowledge, prior studies have not directly and systematically compared how the choice of labeling strategy, independent of model architecture or dataset, affects machine learning performance in VF progression detection, and the relative impact of labeling definition on model learning remains poorly characterized.

The application of machine learning to glaucoma-related tasks has grown substantially in recent years. A comprehensive survey of AI methods in glaucoma diagnosis reported that deep learning accounts for the largest proportion of published approaches (31.5%), yet conventional machine learning methods, particularly support vector machines (SVM), remain widely adopted, representing 25.9% of reported methods, underscoring the continued clinical and methodological relevance of traditional classifiers [15]. Beyond model architecture, the quality and definition of training labels have emerged as a critical determinant of model performance across ophthalmic AI applications. For instance, a recent multicenter study applying ensemble machine learning models, including logistic regression (LR), random forest (RF), and support vector classifier, to electronic health records for glaucoma identification demonstrated that severe class imbalance and heterogeneous data definitions substantially impaired model performance, and that resampling strategies designed to address label distribution directly improved classification outcomes [16]. These findings, alongside evidence from broader medical AI literature [12–14], indicate that the challenge of ground truth definition is not unique to glaucoma but represents a fundamental issue across AI-based clinical applications. The present study addresses this challenge directly by systematically comparing the effect of labeling strategy on machine learning performance in VF progression detection.

In this study, we newly proposed an inclusive “Consensus label,” in which a case was considered progressive if any one of five major progression algorithms classified it as “True.” This label was directly compared with the spatiotemporal rule (“Wiggs’ label”) proposed by Wiggs et al., which emphasizes regional spatial consistency [17,18]. Furthermore, we conducted an ablation analysis to assess the relative contribution of each progression criterion to Consensus label–based model performance and to explore the potential for defining an algorithm-derived functional reference label for glaucoma progression detection. Importantly, the present study is not designed to validate a clinically deployable progression-detection system. Rather, it investigates how the choice of outcome label determines the apparent performance of machine learning classifiers trained on the same dataset, and what this reveals about the learnability and internal structure of each labeling scheme.

## Materials and Methods

This retrospective study was conducted in accordance with the principles of the Declaration of Helsinki. VF data from patients with glaucoma or suspected glaucoma were collected from Pusan National University Hospital, Kosin University Gospel Hospital, Dong-A University Hospital, Busan Paik Hospital, and Pusan National University Yangsan Hospital between 1, June 2004 and 31, January 2021. The study protocol was reviewed and approved by the institutional review boards (IRBs) of Pusan National University Hospital (2203-018-113), Kosin University Gospel Hospital (2018-12-028), Dong-A University Hospital (22–074), Busan Paik Hospital (2023−11179), and Pusan National University Yangsan Hospital (05-2018-172). The requirement for patient consent was waived by the IRBs due to the retrospective nature of the study. The data was accessed for research purpose, and the access periods are as follows: Pusan National University Hospital (25, March, 2022–31, December, 2025), Kosin University Gospel Hospital (30, January, 2019–31, December, 2025), Dong-A University Hospital (25, April, 2022–31, December, 2025), Busan Paik Hospital (11, December, 2023–31, December, 2025), and Pusan National University Yangsan Hospital (16, October, 2018–31, December, 2025). The data were de-identified, and the authors did not have access to information that could identify individual participants.

At all five participating centers, patients underwent comprehensive ophthalmological examinations including best-corrected visual acuity, slit-lamp biomicroscopy, intraocular pressure measurement using Goldmann applanation tonometry, gonioscopy, fundus examination, and keratometry using an auto-kerato-refractometer. Automated perimetry was performed using a Humphrey Visual Field Analyzer 750i (Carl Zeiss Meditec, Dublin, California, USA) with the Swedish Interactive Thresholding Algorithm standard 24–2. To account for the learning effect, the first VF test for each eye was excluded. Reliability criteria for VF selection included a fixation loss rate < 33% and a false-positive rate < 15%. The false-negative rate was not applied as a reliability criterion based on a previous study [19]. Eyes were included if patients were aged ≥ 18 years, had at least five reliable VF examinations, and had an interval of ≥ 28 days between successive tests.

The initial dataset consisted of 364,153 VF examinations from 74,722 patients and 144,123 eyes collected between June 2004 and January 2021. After deduplication and restricting the cohort to patients aged ≥18 years, 347,514 VF examinations remained. Application of reliability criteria (false-positive rate <15% and fixation loss rate <33%) yielded 271,070 VF examinations from 113,739 eyes. After excluding the first VF examination from each eye to minimize learning effects, 157,331 VF examinations remained. Restricting the dataset to eyes with at least five VF examinations resulted in 96,867 VF examinations. Following the segmentation and preprocessing procedures described in the Methods, the final analytical dataset comprised 47,570 VF examinations corresponding to 9,514 eye-episodes from 7,196 eyes and 4,496 patients (S1 Fig).

**Fig 1:**
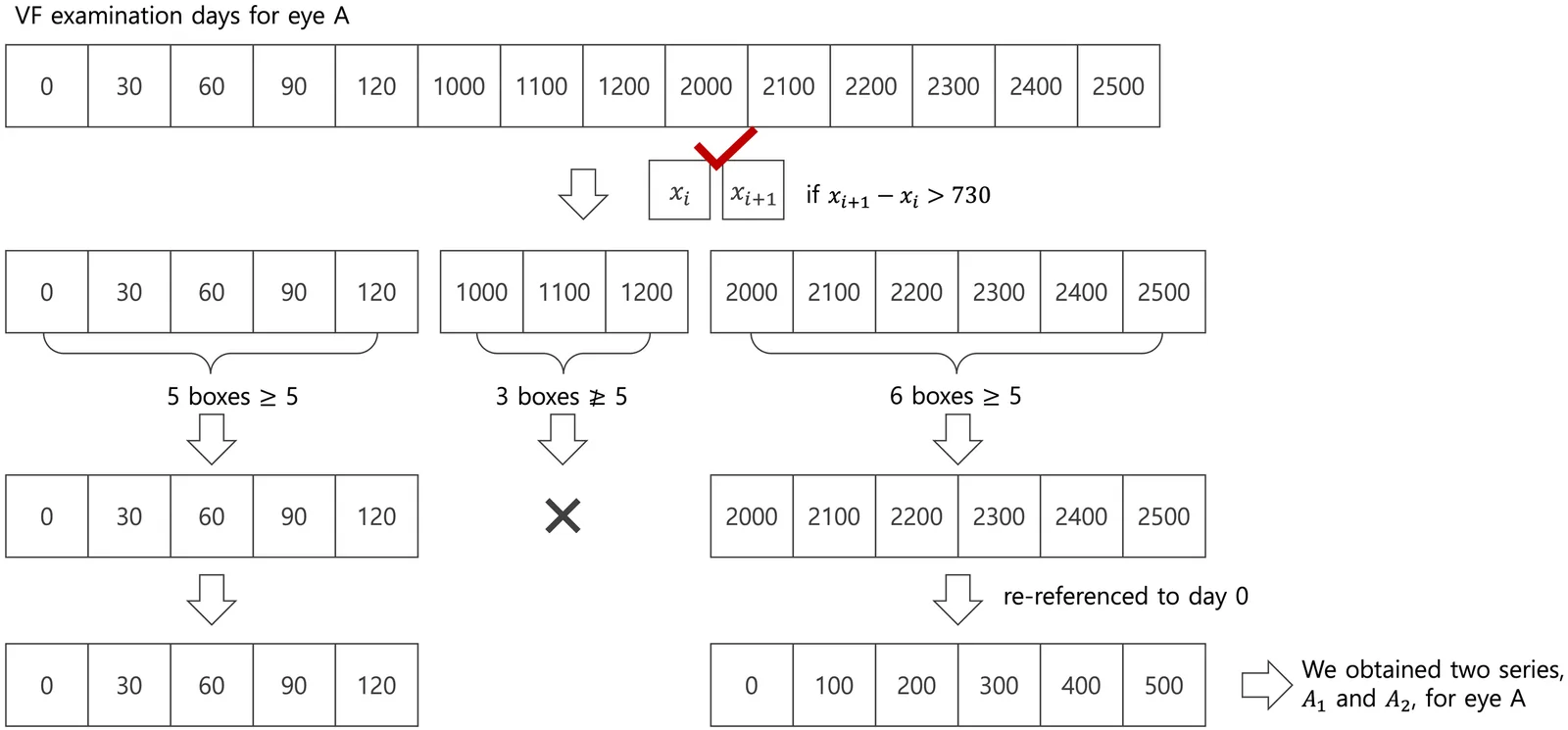
Preprocessing workflow for visual field time series and definition of eye-episodes. Example illustrating the splitting of visual field examinations into segments and re-referencing of time.

**Fig 2:**
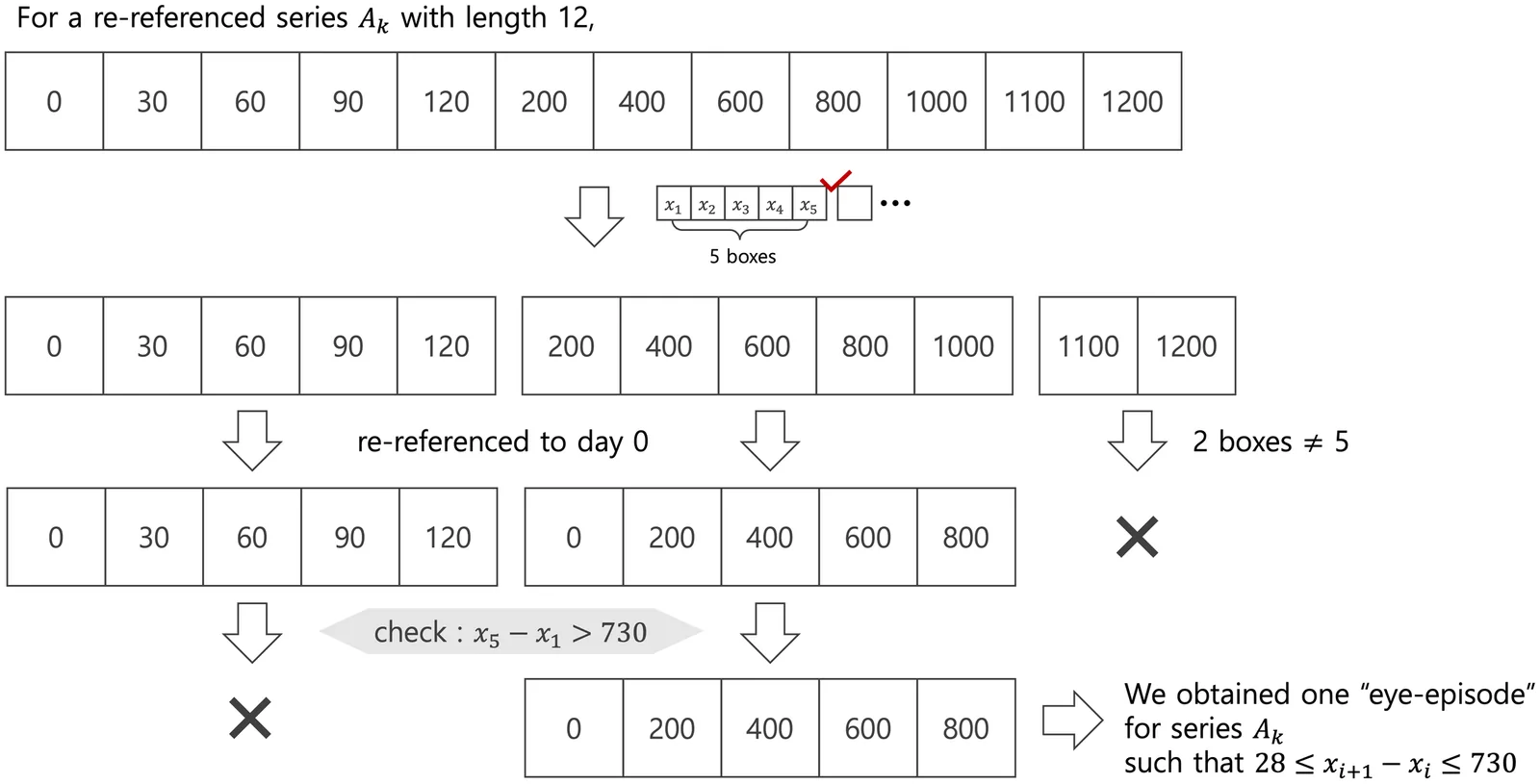
Example of sequential grouping of five consecutive visual field examination within a retained segment.

For each eye, the data were split whenever the interval between consecutive VF examinations exceeded 730 days (2 years). Only segments containing at least five examinations were retained, and the starting date of each segment was re-referenced to time zero (Fig 1). This re-referencing procedure was performed to define the “Consensus label” and the “Wiggs label” described in the following section. For each segmented series with length 𝑛 ≥ 5, the data were further subdivided sequentially into groups of five consecutive VF examinations. Thus, each segment was partitioned into [n/5] subsegments. Any remaining examinations (i.e., the remainder when n was divided by 5) were excluded from the analysis (Fig 2). The resulting subsegments were defined as eye-episodes. This preprocessing framework enabled the inclusion of a larger dataset by retaining only valid VF sequences that met the predefined criteria after removal of the initial VF test.

Five methods were employed to identify perimetric progression as follows: (1) the AGIS scoring system [20], (2) the CIGTS scoring system [21], (3) MD slope [22], (4) VFI slope [22], and (5) PLR [3].

The AGIS and CIGTS scoring systems have been described in detail elsewhere [20,21]. Progression was defined as an increase of ≥ 4 and ≥ 3 points, respectively, relative to the mean of the first two tests, confirmed on three consecutive examinations. For MD and VFI, the slope over time was calculated using linear regression. If the slope was negative and its p-value was < 0.05, the eye was classified as progressing [22]. For PLR, ordinary least squares regression of the total deviation value (TDV) over time was performed for each of the 52 VF test locations. Progression was defined as ≥ 3 locations showing a significant regression slope of −1 dB/year or worse (P < 0.01) [3]. VF worsening was considered present if at least one of the following five algorithms identified progression: AGIS, CIGTS, MD slope, VFI slope, and PLR. This reference standard was termed the “Consensus label.”

In addition, pattern deviation plots were divided into six peripheral regions (superior and inferior nasal steps, superior and inferior Bjerrum areas, and superior and inferior temporal wedge regions) and two paracentral regions [17]. In each peripheral region, a VF defect was defined as a cluster of at least three adjacent test points conforming to nerve fiber layer topology, with −5 dB or worse at each point. For the paracentral region, a VF defect was defined as at least two adjacent points with a summed deviation of −15 dB or more. If the final VF in the series showed no VF defect in any region, the status was classified as no progression.

Progression was defined using three criteria: (1) the presence of a VF defect in one or more regions in the final VF that was reproduced on a prior VF but not present in the baseline VF; (2) a VF defect present in one or more regions on the final two VFs that was worse than the first two tests, defined as a worsening of −3 dB or more in the average pattern deviation value (PDV) across all test points within the region; or (3) a worsening of −3 dB or more in the average MD values of the final two VFs compared with the average of the first two VFs [18]. If progression was identified by at least one of these three criteria, the eye-episode was labeled as progression and this definition was termed the “Wiggs’ label.”

In this study, predictive models were implemented in Python 3.12. For classification of VF progression, we employed scikit-learn version 1.7.2. and XGBoost version 3.2.0. We utilized the following commonly used machine learning classifiers: SVM, RF, LR, and XGBoost. Prior to model training, all input features were standardized using the StandardScaler implementation in scikit-learn. To ensure consistent preprocessing across classifiers, each model was implemented within a scikit-learn pipeline incorporating the standardization step. No additional hyperparameter optimization was performed because the primary aim of this study was to compare the effect of labeling strategies under the same modeling framework rather than to maximize the performance of individual classifiers. Therefore, all models were trained using the default hyperparameters of each package, except for the fixed random seed (random_state = 0). No class weighting, resampling, or threshold adjustment was applied, and the default decision threshold of 0.5 was used for binary classification.

Because machine learning classifiers require inputs of fixed length, the unit of analysis in this study was defined as an eye-episode, with all inputs standardized to five consecutive VF examinations. Following the method described in reference [4], each eye-episode was divided into an early phase (the first two examinations) and a late phase (the last three examinations). For AGIS score, CIGTS score, MD, and VFI, the feature values were calculated as the differences between the late-phase and early-phase means. For TDV and PDV, the mean values for the early and late phases were first calculated at each of the 52 VF locations. Pointwise changes were then obtained by subtracting the early-phase mean from the late-phase mean at each location. The mean and standard deviation of these pointwise changes across the 52 locations were calculated for each eye-episode. MD slope and VFI slope were calculated using linear regression across the five consecutive VF examinations within each eye-episode, with time in years from the first examination in the episode as the independent variable. The features used in this study were the AGIS score, CIGTS score, MD, MD slope, VFI, VFI slope, and the mean and standard deviation of TDV and PDV for each eye-episode. Of all eye-episodes, 80% were used as the training dataset and the remaining 20% were used as the test dataset.

Using an identical training dataset, two separate detection experiments were performed. The first experiment evaluated classification performance on the test dataset using the Consensus label, whereas the second experiment evaluated performance using the Wiggs’ label. To ensure an objective comparison between the two labeling standards, no deliberate hyperparameter optimization was performed for any model. Model performance was evaluated using accuracy, sensitivity, specificity, precision, and F1 score, derived from the confusion matrix. Discriminative performance was further assessed using the area under the receiver operating characteristic curve (AUC) and precision–recall (PR) curves, summarized by average precision (AP). 95% confidence intervals were calculated for AUC and AP. Because no formal statistical comparisons between labeling strategies or classifiers were performed, performance comparisons were interpreted descriptively.

## Results

After applying the preprocessing procedures, we identified 9,514 eye-episodes from 4,496 patients, comprising a total of 47,570 VF examinations for analysis (Table 1).

**Table 1:** Demographic characteristics of all subjects.

Characteristics	All data
Total number of visual field tests	47,570
Total number of patients	4,496
Total number of eyes	7,196
Total number of eye-episode	9,514
Age (years), mean ± SD	56.735 ± 14.425
Initial visual field MD (dB), mean ± SD	−6.053 ± 6.238
Follow-up duration (years), mean ± SD	4.434 ± 2.596
Mean number of visual field tests per eye-episode, mean ± SD	5.0 ± 0.0
MD ≥ −6 dB	30,724
−6 dB > MD ≥ −12 dB	8,512
−12 dB > MD	8,334

MD, mean deviation

The means and standard deviations of the features, as well as age and the intervals between VF examinations, for each dataset are presented in Table 2.

**Table 2:** Demographic and visual field–derived features used as machine learning inputs in the training and test sets. Age and the interval between visual field examinations are reported as the mean and standard deviation for each eye-episode, whereas all other variables are presented as the mean and standard deviation of the change values, calculated as the difference between the late-phase and early-phase averages within each eye-episode.

		Training data (n = 7611)	Test data (n = 1903)
Dataset characteristics	Age (year), mean ± SD	56.755 ± 14.484	56.656 ± 13.932
	The intervals between visual field examinations (year), mean ± SD	1.933 ± 0.546	1.924 ± 0.536
	Initial MD ≥ −6 dB	7536	1886
	−6 dB > Initial MD ≥ −12 dB	68	16
	−12 dB > Initial MD	7	1
	Consensus label: stable/ progressing, n (%)	5333 (70.1%)/ 2278 (29.9%)	1334 (70.1%)/ 569 (29.9%)
	Wiggs’ label: stable/ progressing, n (%)	4814 (63.3%)/ 2797 (36.7%)	1203 (63.2%)/ 700 (36.8%)
	Center balance, n (%)	Dong-A:3262 (42.9%), Busan Paik: 979 (12.9%), Kosin: 236 (3.1%), Pusan: 2627 (34.5%), Yangsan: 507 (6.7%)	Dong-A: 784 (41.2%), Busan Paik: 247 (13.0%), Kosin: 68 (3.6%), Pusan: 666 (35.0%), Yangsan: 138 (7.3%)
Features used for machine learning	AGIS score, mean ± SD	0.179 ± 1.744	0.176 ± 1.718
	CIGTS score, mean ± SD	0.012 ± 2.561	−0.006 ± 2.533
	MD, mean ± SD	−0.154 ± 1.87	−0.149 ± 1.81
	MD slope, mean ± SD	−0.14 ± 0.429	−0.129 ± 0.394
	VFI, mean ± SD	−1.026 ± 5.408	−1.08 ± 5.239
	VFI slope, mean ± SD	−0.469 ± 1.466	−0.427 ± 1.373
	Mean 52 TDV mean, mean ± SD	−0.149 ± 1.905	−0.143 ± 1.861
	Mean 52 TDV SD, mean ± SD	2.52 ± 1.419	2.504 ± 1.382
	Mean 52 PDV mean, mean ± SD	−0.21 ± 1.16	−0.231 ± 1.133
	Mean 52 PDV SD, mean ± SD	2.519 ± 1.419	2.503 ± 1.383

AGIS, Advanced Glaucoma Intervention Study; CIGTS, Collaborative Initial Glaucoma Treatment Study; MD, mean deviation; PLR, pointwise linear regression; PDV, pattern deviation value; TDV, total deviation value; VFI, Visual Field Index

Using the Consensus label, defined as progression detected by any of the five algorithms, 2,847 eye-episodes (29.9%) were classified as progressing. The proportion of progression varied across individual criteria, with the highest rate observed for the VFI slope and the lowest rate for AGIS criterion (Table 3).

**Table 3:** Rate of progression for each algorithm and consensus label.

	AGIS	CIGTS	MD slope	VFI slope	PLR	Consensus label
Stable	8,935	8,418	7,705	7,551	8,455	6,667
Progressing	579	1,096	1,809	1,963	1,059	2,847

AGIS, Advanced Glaucoma Intervention Study; CIGTS, Collaborative Initial Glaucoma Treatment Study; MD, mean deviation; PLR, pointwise linear regression; VFI, Visual Field Index. Counts are reported at the eye-episode level, with each eye-episode consisting of five consecutive visual field examinations.

When trained with the Consensus label, all models demonstrated high specificity (≥0.995) while maintaining moderate-to-high sensitivity (0.817–0.852). The RF model achieved the highest sensitivity (0.852), followed by XGBoost (0.849), whereas LR showed the highest specificity (1.000). These results indicate that Consensus label–based training captures a broad spectrum of progression patterns without compromising false-positive control, suggesting that even less complex models can achieve high classification performance relative to the training label under this inclusive labeling scheme (Table 4).

**Table 4:** Classification performance of machine learning models for detecting visual field progression using the Consensus label.

	Accuracy (95% CI)	Sensitivity (95% CI)	Specificity (95% CI)	Precision (95% CI)	F1 score (95% CI)
SVM	0.947 (0.936-0.956)	0.824 (0.790-0.853)	0.999 (0.998-1.000)	0.998 (0.993-1.000)	0.903 (0.882-0.920)
RF	0.955 (0.944-0.964)	0.852 (0.821-0.879)	0.999 (0.996-1.000)	0.996 (0.990-1.000)	0.919 (0.899-0.934)
LR	0.945 (0.935-0.955)	0.817 (0.783-0.846)	1.000 (1.000-1.000)	1.000 (1.000-1.000)	0.899 (0.879-0.917)
XGBoost	0.951 (0.941-0.960)	0.849 (0.817-0.877)	0.995 (0.991-0.998)	0.986 (0.974-0.996)	0.912 (0.891-0.929)

CI, Confidence Interval; SVM, Support Vector Machine; RF, Random Forest; LR, Logistic Regression

**Fig 3:**
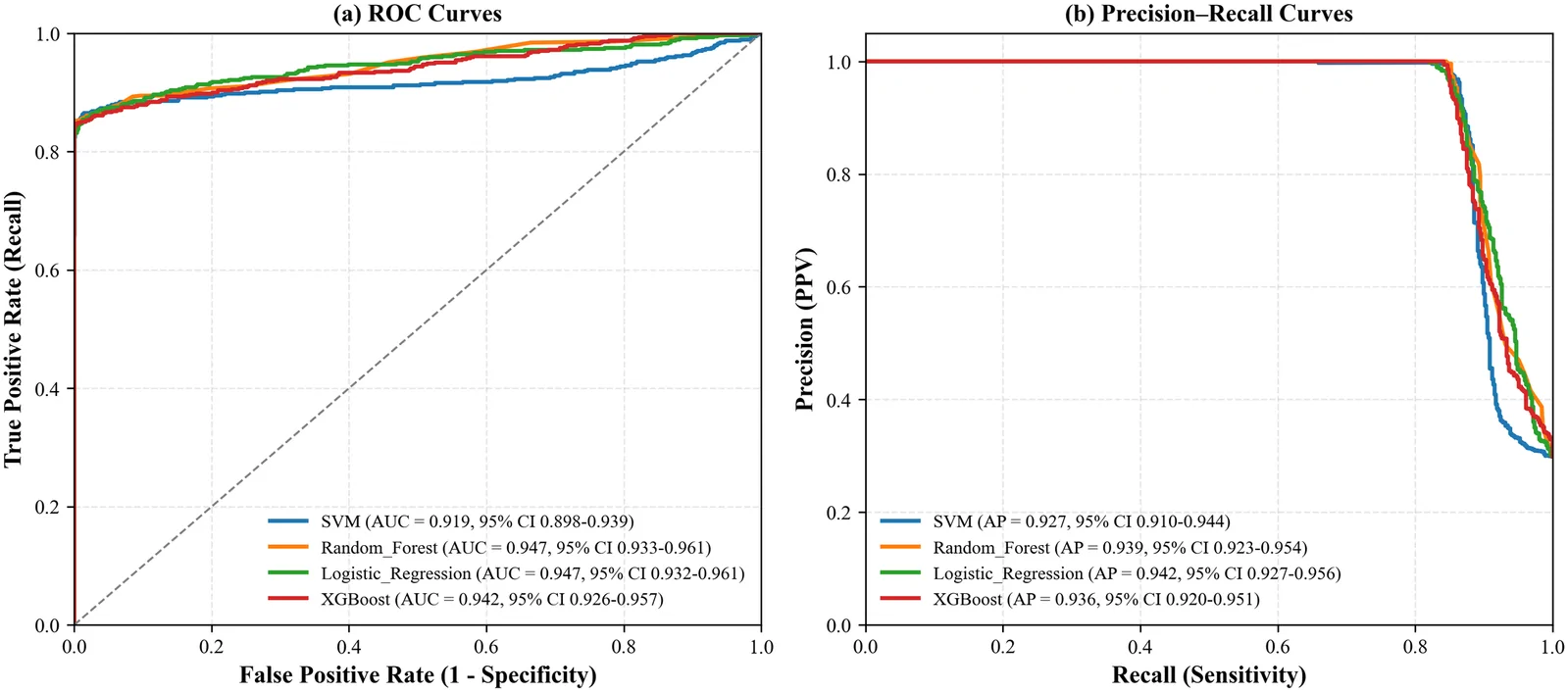
Receiver operating characteristic (ROC) and precision–recall (PR) curves of four machine learning classifiers (SVM, RF, LR, and XGBoost) for detecting visual field progression using the Consensus label. LR and RF achieved the highest discriminative performance (AUC = 0.947), followed by XGBoost (AUC = 0.942) and SVM (AUC = 0.919). PR curve analysis showed comparable precision–recall performance across models, with LR (AP = 0.942) and RF (AP = 0.939) demonstrating the highest average precision. (a) ROC curves. (b) Precision–recall curves.

Receiver Operating Characteristic (ROC) and Precision–Recall (PR) analyses for the Consensus label, including 95% confidence intervals for AUC and average precision (AP), are presented in Fig 3. ROC curve analysis revealed that LR and RF showed the highest observed AUC values among the evaluated models (AUC = 0.947), followed by XGBoost (AUC = 0.942) (Fig 3a). In contrast, SVM showed relatively lower discrimination (AUC = 0.919). PR analysis demonstrated high and comparable performance across models (AP range, 0.927–0.942), with LR and RF showing the highest reliability (Fig 3b). Consistent with the ROC analysis, RF showed slightly higher sensitivity with comparable F1 scores, whereas LR maintained similarly strong discriminative performance.

Using the Wiggs’ label, which defines progression as detection by any of the three criteria, 36.8% of eye-episodes were classified as progressing. PDV-based regional worsening and the appearance of new regional defects identified a larger number of progressing cases compared with MD-based progression alone (Table 5).

**Table 5:** Rate of progression for each algorithm and the Wiggs’ label.

Progression	PDV progression	MD progression	Visual field defect in a new region	Wiggs’ label
Stable	7,077	8,163	7,196	6,017
Progressing	2,437	1,351	2,318	3,497

MD, mean deviation; PDV, pattern deviation value. Counts are reported at the eye-episode level, with each eye-episode consisting of five consecutive visual field examinations.

When trained with the Wiggs’ label, all models showed a marked decrease in sensitivity (0.630–0.717) compared with the Consensus label, while maintaining moderately high specificity (0.867–0.922). The RF model achieved the highest sensitivity (0.717), whereas SVM preserved relatively high specificity (0.922) (Table 6).

**Table 6:** Classification performance of machine learning models for detecting visual field progression using the Wiggs’ label.

	Accuracy (95% CI)	Sensitivity (95% CI)	Specificity (95% CI)	Precision (95% CI)	F1 score (95% CI)
SVM	0.826 (0.809-0.843)	0.660 (0.624-0.694)	0.922 (0.907-0.936)	0.831 (0.802-0.859)	0.736 (0.707-0.761)
RF	0.828 (0.811-0.844)	0.717 (0.683-0.747)	0.892 (0.875-0.910)	0.794 (0.766-0.825)	0.754 (0.728-0.776)
LR	0.811 (0.794-0.827)	0.630 (0.594-0.665)	0.916 (0.901-0.932)	0.814 (0.782-0.845)	0.710 (0.683-0.737)
XGBoost	0.799 (0.780-0.816)	0.681 (0.647-0.716)	0.867 (0.848-0.888)	0.749 (0.715-0.782)	0.714 (0.685-0.739)

CI, Confidence Interval; SVM, Support Vector Machine; RF, Random Forest; LR, Logistic Regression

**Fig 4:**
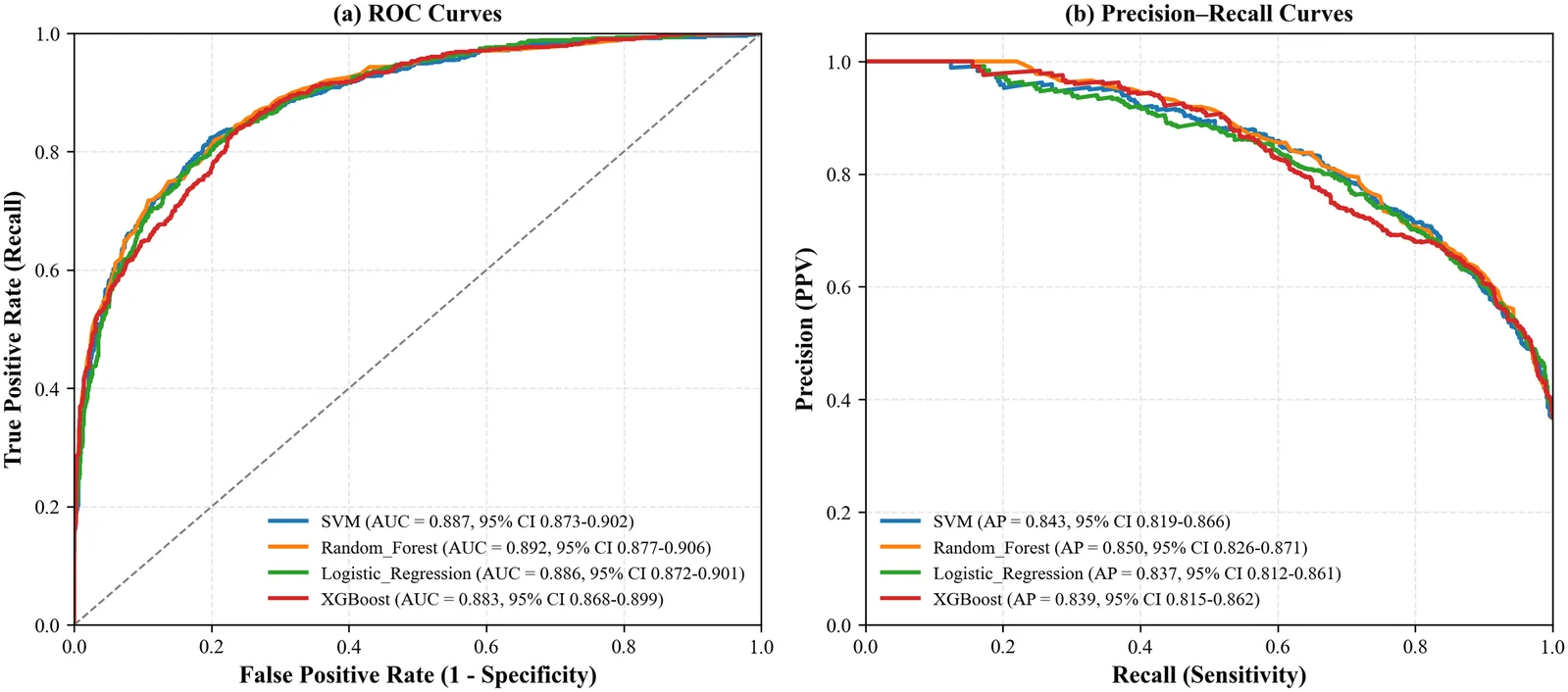
Receiver operating characteristic (ROC) and precision–recall (PR) curves of four machine learning classifiers (SVM, RF, LR, and XGBoost) for detecting visual field progression using the Wiggs’ label. RF achieved the highest discriminative performance (AUC = 0.892), followed by SVM (AUC = 0.887), LR (AUC = 0.886), and XGBoost (AUC = 0.883). PR curve analysis showed comparable precision–recall performance across models, with RF (AP = 0.850) demonstrating the highest average precision. (a) ROC curves. (b) Precision–recall curves.

ROC and PR analyses for the Wiggs’ label, including 95% confidence intervals for AUC and AP, are presented in Fig 4. ROC curve analysis using the Wiggs’ label showed that RF showed the highest observed AUC among the evaluated classifiers (AUC = 0.892), followed by SVM (AUC = 0.887) and LR (AUC = 0.886). In contrast, XGBoost demonstrated lower performance (AUC = 0.883) (Fig 4a). PR analysis showed similar performance across models (AP range, 0.837–0.850), with RF and SVM demonstrating the highest values (Fig 4b). Consistent with the ROC and PR analyses, RF achieved the highest discriminative performance, whereas SVM demonstrated comparatively stable classification characteristics. Compared with the Consensus label, models trained using the Wiggs’ label showed consistently lower sensitivity while maintaining moderate-to-high specificity.

To provide full transparency of the underlying classification results, confusion matrices for all four classifiers under both labeling strategies are presented in S2 Fig. These matrices show the absolute counts of true positives, false positives, true negatives, and false negatives, from which the reported sensitivity, specificity, and precision values are derived.

To assess the relative importance of each of the five progression algorithms to the Consensus label–based model, we performed an ablation analysis. To minimize bias caused by incorporating variables directly involved in defining each progression criterion, the corresponding features were excluded from the input feature set one at a time while retaining the Consensus label as the target, and models were trained and evaluated using the remaining features among the ten commonly used features. Ablation analysis showed that exclusion of event-based features (AGIS and CIGTS scores) resulted in minimal performance changes, whereas removal of global trend features (MD and VFI slopes) reduced sensitivity despite preserved specificity. Exclusion of PLR-related features led to a balanced decline in performance, indicating complementary contributions of pointwise trend information (S1-S5 Tables).

To further examine the relationship between input features and progression labels, and to address the potential concern that model performance under the Consensus label may partly reflect overlap between label-defining criteria and predictor variables, we calculated point-biserial correlation coefficients between each continuous input feature and both progression labels (S6 Table). Although the Consensus label showed stronger correlations with global trend-based features such as MD slope (r = −0.50) and VFI slope (r = −0.49), consistent with its algorithmic definition, the Wiggs’ label demonstrated comparatively stronger associations with spatial variability features, particularly TDV standard deviation (r = 0.49) and PDV standard deviation (r = 0.49). This divergence in feature-label correlation patterns indicates that each label captures a distinct and internally coherent dimension of VF deterioration. Importantly, the performance differences observed between the two labeling strategies are therefore not solely attributable to direct overlap between input features and label-defining criteria, but rather reflect the degree to which each label is aligned with the overall feature space available in this study. The lower detection performance observed under the Wiggs’ label likely reflects the relative underrepresentation of spatially granular features in the current input set, rather than the clinical inferiority of the label itself.

We performed an additional sensitivity analysis using a patient-level split, and the results have been provided in S7-S9 Tables. After ensuring that all eye-episodes originating from the same patient were assigned exclusively to either the training or test dataset, the relative performance trends across labeling strategies and the main study conclusions remained consistent. These findings suggest that the primary observations of this study are not solely dependent on eye-episode–level data partitioning and that the comparative results of the proposed labeling strategies are relatively robust (S7-S9 Tables).

To assess the generalizability of the models across participating institutions, a leave-one-center-out validation analysis was additionally performed. In each iteration, one of the five centers was held out as an independent test set while models were trained on data from the remaining four centers. Under the Consensus label, models consistently achieved high accuracy (0.934–0.964) and sensitivity (0.786–0.890) with near-perfect specificity (0.987–1.000) across all held-out centers. Under the Wiggs’ label, models maintained moderate-to-high accuracy (0.791–0.834) and specificity (0.821–0.926), with sensitivity ranging from 0.607 to 0.757. The relative performance patterns across labeling strategies were consistent with those observed in the primary analysis, supporting the robustness of the labeling effect across heterogeneous clinical settings (S10 Table).

## Discussion

Machine learning models trained with the Consensus label consistently demonstrated higher classification performance than those trained with the Wiggs’ label, achieving improved sensitivity without compromising specificity. This suggests that integrating heterogeneous progression signals enables robust learning beyond simple inflation of positive cases. The Consensus label successfully incorporated subtle or localized glaucomatous changes that might have been overlooked because of disagreement among individual algorithms, while still maintaining robust control over false-positive detections.

This trend is consistent with the previously reported low inter-algorithm agreement (κ = 0.12–0.52) described by Saeedi et al. [5], suggesting that the Consensus label provides a practical solution to mitigate such discrepancies. The Consensus label may help reduce false negatives while capturing clinically meaningful progression signals that may be overlooked by any single algorithm, thereby serving as a algorithm-derived functional reference label for detecting glaucomatous progression.

The Wiggs’ label used in this study is a region-level, spatially consistent approach that defines progression by combining PDV-based clustering and adjacency conditions in both peripheral and paracentral regions, together with event confirmation rules such as “two consecutive abnormal tests” [17,18]. This region-level thresholding framework favors reduction of false positives (higher specificity) but demonstrates an event–threshold trade-off, being less sensitive to subtle or early glaucomatous changes. Such trade-offs have been repeatedly observed in previous studies comparing VF progression criteria [3,23,24]. Previous studies consistently report a trade-off between sensitivity and specificity across VF progression criteria, with conservative event-based rules favoring specificity and trend-based approaches offering higher sensitivity [3,23,24]. This pattern also applies to the Wiggs’ rule, in which stricter requirements for spatial consistency and repeated confirmation increase specificity but reduce sensitivity when focal defects fail to meet regional criteria.

Although the Wiggs’ label was described as a more conservative progression framework, this characterization refers to the structure of the progression criteria rather than the overall prevalence of progression-positive cases. Although the Wiggs’ label yielded a higher progression-positive rate than the Consensus label in our dataset, it incorporates event-based criteria such as PDV-based regional worsening and the appearance of new regional defects, which may identify localized spatial changes not captured by global trend-based measures such as MD slope or VFI slope. Consequently, a labeling framework can be methodologically conservative while still classifying a relatively larger number of eye-episodes as progressing. This distinction helps reconcile the apparently counterintuitive relationship between the stricter progression criteria and the observed class distribution.

The higher AUC and precision–recall values observed with the Consensus label indicate that models trained under this labeling scheme can achieve both high sensitivity and high positive predictive value across a wide operating range. Importantly, the improvement in sensitivity was not accompanied by a loss of specificity, suggesting that the Consensus label does not merely inflate positive cases but instead enables the model to learn a broader spectrum of robust progression patterns shared across heterogeneous algorithms. In contrast, models trained with the Wiggs’ label showed lower AUC and average precision, reflecting the conservative, region-based nature of this labeling strategy, which prioritizes spatial consistency and repeated confirmation at the cost of reduced sensitivity to early or localized changes. Together, these findings suggest that the Consensus label may be better suited for sensitive and comprehensive identification of progression across heterogeneous clinical presentations, whereas the Wiggs’ label serves as a more stringent confirmatory criterion emphasizing spatial consistency.

Ablation analysis demonstrated that no single progression criterion dominated model performance under the Consensus label. While exclusion of event-based features (AGIS and CIGTS scores) resulted in minimal performance changes, removal of global trend features (MD and VFI slopes) led to reduced sensitivity despite preserved specificity. Exclusion of PLR-related features produced a more balanced decline in performance, highlighting the complementary contribution of pointwise trend information in integrating heterogeneous progression signals. Together, these findings indicate that the Consensus label derives its robustness from the integration of diverse progression patterns rather than reliance on any single criterion, providing a practical ground-truth framework that maintains high specificity while enhancing sensitivity in large multicenter datasets. In this context, the Wiggs’ label may serve as a complementary criterion, better suited for confirmatory assessment emphasizing spatial consistency.

A major strength of this study lies in providing a direct and controlled comparison of labeling strategies as the primary study variable in machine learning–based VF progression detection. While prior studies have employed various progression algorithms or consensus-based definitions as ground truth labels, the systematic evaluation of how labeling definition itself, independent of model architecture, determines model performance has been limited. By comparing models trained on the same dataset, input feature set, and model architecture using different ground-truth labeling strategies, this study demonstrates that the definition of ground truth is a critical determinant of performance in machine learning–based progression detection. Because different labeling strategies inherently result in differences in class prevalence and classification difficulty, the observed performance differences reflect the impact of alternative ground-truth definitions under otherwise consistent experimental conditions. A second strength is the scale and rigor of data curation. The dataset comprised 4,496 patients, 9,514 eye-episodes, and 47,570 valid VF tests, and was significantly larger and more diverse than those of previous single-center studies with limited sample sizes. Strict inclusion criteria regarding test intervals, reliability indices, and temporal continuity minimized noise-driven variability and improved data quality. A third strength is the clinical interpretation of labeling trade-offs, providing practical guidance for selecting ground truths in machine learning–based progression detection. This comparison offers practical guidance for defining ground truths in future machine learning–based clinical decision frameworks.

The robustness of the primary findings was further supported by two additional validation analyses. In a patient-level split analysis, all eye-episodes from the same patient were assigned exclusively to either the training or test set, thereby eliminating potential data leakage arising from episode-level partitioning. Performance trends across labeling strategies remained consistent with those observed in the primary analysis (S7-S9 Tables). Similarly, in a leave-one-center-out analysis in which each of the five participating centers was held out in turn as an independent test set, the relative performance patterns were preserved across all centers under both labeling strategies (S10 Table). Together, these analyses indicate that the observed labeling effect is not an artifact of the data-splitting strategy or center-specific characteristics, and that the performance differences between the Consensus and Wiggs’ labels generalize across heterogeneous multicenter settings.

A potential concern is that the observed performance difference between the two labeling strategies may partly reflect an asymmetry in input representation rather than a true difference in label quality. The Consensus label is algorithmically aligned with global trend-based features such as MD slope and VFI slope, which were among the strongest predictors in the current feature set (r = −0.50 and −0.49, respectively), potentially conferring an inherent advantage to Consensus label–based models. However, examination of feature-label correlations revealed that the Wiggs’ label showed comparatively stronger associations with spatial variability features, particularly TDV standard deviation and PDV standard deviation (r = 0.49 for both), which were less prominent predictors of the Consensus label (S6 Table). This pattern suggests that each label captures a distinct and internally coherent dimension of VF deterioration, and that the lower predictive performance observed under the Wiggs’ label reflects the relative underrepresentation of spatially granular features in the current input set rather than the clinical inferiority of the label itself. The present results should not be interpreted as demonstrating the clinical validity of either label. Rather, they demonstrate the relative learnability and internal consistency of two algorithm-derived functional progression labels within the selected feature space. Future studies incorporating raw VF maps or richer regional representations may better capture the spatially organized defect patterns underlying the Wiggs’ label and yield more balanced model performance across labeling strategies.

Several limitations of this study warrant consideration. First, the Consensus label was derived exclusively from functional VF data and was not linked to structural optical coherence tomography (OCT) evidence, such as retinal nerve fiber layer or macular ganglion cell layer thickness measurements. This is a critical limitation, as structural changes detected by OCT are known to frequently precede functional VF loss in glaucoma, suggesting that some eyes with genuine early structural progression may have been labeled as non-progressing under the current framework [25,26]. The absence of structural validation means that the Consensus label, despite its functional sensitivity, cannot be considered a fully comprehensive ground truth. Future studies incorporating longitudinal OCT data alongside VF measurements into the labeling strategy would be valuable to assess whether a combined structure–function label further improves model performance and clinical validity [26]. Second, conventional machine learning models were used instead of deep learning approaches to facilitate controlled cross-label comparisons and clearer interpretation of labeling effects. Although deep learning methods have shown promise in ophthalmic AI, the effective sample size and primary objective of isolating labeling effects favored the use of conventional models, ensuring methodological consistency across experiments.

This study is the first machine learning investigation to propose a new Consensus label (progression defined as ≥ 1 positive criterion) to address discrepancies among conventional VF progression algorithms and to directly compare it with the region-based Wiggs’ label. Models trained with the Consensus label achieved high AUCs and precision–recall performance together with unexpectedly strong specificity, demonstrating a balanced performance that favors sensitive identification of progression without an excessive false-positive burden. In contrast, the Wiggs’ label showed strength in ensuring spatial consistency and confirmatory progression assessment, suggesting that the two labeling schemes can serve complementary roles in clinical application.

In summary, this study demonstrates that the labeling strategy used to define glaucomatous progression is a critical determinant of apparent machine learning performance, and that differences in model performance across labeling schemes primarily reflect model-label compatibility given the input feature space. Whether the Consensus label constitutes a clinically valid ground truth remains an open question that requires prospective validation incorporating structural endpoints.

## Supporting information

S1 Fig Flowchart of patient selection and preprocessing for the visual field dataset. (PNG)

S2 Fig Confusion matrices for progression classification. (PNG)

S1 Table Classification performance of machine learning models after excluding Advanced Glaucoma Intervention Study-related features. (DOCX)

S2 Table Classification performance of machine learning models after excluding Collaborative Initial Glaucoma Treatment Study-related features. (DOCX)

S3 Table Classification performance of machine learning models after excluding mean deviation-related features. (DOCX)

S4 Table Classification performance of machine learning models after excluding Visual Field Index-related features. (DOCX)

S5 Table Classification performance of machine learning models after excluding pointwise linear regression analysis-related features. (DOCX)

S6 Table Correlation between input features and progression labels (Consensus label and Wiggs’ label). (DOCX)

S7 Table Demographic and visual field–derived features used as machine learning inputs in the training and test sets for patient-level split analyses. (DOCX)

S8 Table Classification performance of machine learning models for detecting visual field progression using the Consensus label with patient-level train–test splitting. (DOCX)

S9 Table Classification performance of machine learning models for detecting visual field progression using the Wiggs’ label with patient-level train–test splitting. (DOCX)

S10 Table Leave-one-center-out validation analysis. (DOCX)
