# Supplementary material for: Labeling matters: A multicenter machine learning study on visual field progression in Glaucoma
Source: PLoS One. 2026 Aug 28;21(8):e0357179. doi: 10.1371/journal.pone.0357179 (PMC13524274; doi:10.1371/journal.pone.0357179)
Supplement: Supplementary file 1 [file pone.0357179.s013.pdf]

Comments to authors

Recommendation: Major revision

This manuscript addresses a clinically relevant and methodologically important question: how ground-truth labeling strategy affects machine learning performance for detecting visual field progression in glaucoma. The multicenter design, relatively large sample size, and direct comparison of two labeling approaches are clear strengths. The study also raises an issue of broad relevance in medical AI, namely that label definition can materially influence downstream model performance. However, several aspects of the current analytic design and interpretation limit the strength of the conclusions, and these issues should be addressed before the manuscript can be considered for acceptance.

Major comments

1. The study is framed as prediction, but the present design appears closer to retrospective within-window classification: The unit of analysis is a 5-visit eye-episode. The labels are defined within that same episode, and the model inputs are also derived from the same episode by comparing the first two visits with the last three visits. In particular, the Wiggs label explicitly uses the final visit or final two visits in its definition. Because late-episode information contributes both to the target and to the predictor set, the present design appears to support retrospective classification of already observed progression within a fixed window more than true future prediction. Accordingly, claims such as "early progression detection" and "early warning systems" should be moderated unless the authors can demonstrate a strictly forward-looking prediction framework.

2. There is substantial overlap between label construction and model inputs, raising a serious concern about circularity: The Consensus label is defined by AGIS, CIGTS, MD slope, VFI slope, and PLR. However, the model inputs also include AGIS, CIGTS, MD, MD slope, VFI, VFI slope, and TDV/PDV-based summary variables. This means that the classifiers are trained using predictors that substantially overlap with the target definition itself. The ablation analysis is useful, but it does not fully resolve the core concern because the principal reported model performances still rely on overlapping labels and features. At minimum, this issue should be discussed more explicitly. Ideally, the authors should provide a sensitivity analysis using features that are independent of the criteria used to define the labels.

3. The manuscript should clarify the train-test split in enough detail to rule out patient-level or eye-level leakage: The paper states that the unit of analysis is the eye-episode, that 9,514 eye-episodes from 4,496 patients were analyzed, and that 80% of eye-episodes were assigned to training and 20% to testing. However, it is not stated whether the split was grouped by patient or by eye. If multiple episodes from the same eye, or both eyes from the same patient, were allowed to appear in both training and test sets, the reported performance could be optimistic. The authors should clarify this explicitly and, preferably, provide a grouped patient-level split as a sensitivity analysis.

4. The multicenter nature of the dataset is a strength, but the current validation strategy does not fully support the manuscript's generalizability claims: The manuscript emphasizes

heterogeneous clinical settings and concludes that the proposed labeling strategy supports robust and generalizable AI systems. However, the Methods describe a single 80/20 split rather than center-held-out validation or a leave-one-center-out design. If data from the same institutions appear in both training and test sets, institution-specific patterns may be shared across splits. A center-level validation strategy would provide much stronger support for the generalizability claims, or alternatively the wording in the Abstract and Discussion should be moderated.

5. The reporting of the predictor set is internally inconsistent and should be corrected: The Methods state that the model uses 10 features, including the mean and standard deviation of TDV and PDV. However, Table 2 lists age, interval between examinations, AGIS, CIGTS, MD, MD slope, VFI, VFI slope, Mean 52 TDV, and Mean 52 PDV, but does not show the standard deviation of TDV or the standard deviation of PDV. In addition, age and interval are described in Table 2 as machine learning inputs, yet they are not included in the explicit list of 10 features in the Methods. The authors should reconcile the Methods and Table 2 so that the exact predictor set is unambiguous.

6. The statistical reporting should be strengthened with uncertainty estimates and clearer comparison of labeling strategies: The paper reports point estimates for accuracy, sensitivity, specificity, precision, F1 score, AUC, and average precision, but I did not see confidence intervals, repeated resampling, or formal statistical comparisons between models or between the two labeling strategies. Because the central claim is that label definition materially affects model performance, uncertainty around these differences should be quantified. Bootstrapped confidence intervals and appropriate comparisons for AUC and threshold-based metrics would strengthen the manuscript considerably.

7. The Introduction is generally clear, but several framing claims should be moderated or better justified: The Introduction appropriately summarizes the problem of low agreement among visual field progression algorithms and motivates the study question. However, the novelty claim that no previous study has systematically compared how labeling strategy affects machine learning performance should be phrased more cautiously, especially because the manuscript itself cites prior consensus-based work. In addition, the rationale for defining the proposed Consensus label as positivity under any one of five criteria is underdeveloped, particularly given that the cited prior consensus-based approach used a much stricter threshold. Finally, the phrase “optimized ground truth” appears stronger than the present study design can support, because the labels are not externally validated against structural OCT, expert adjudication, or long-term clinical outcomes.

Minor comments

1. The interpretation of the Wiggs label as “stricter” or more “conservative” requires clearer explanation, because its observed positive rate is higher than that of the Consensus label. This may be explainable, but it is counterintuitive and should be discussed explicitly so that readers can reconcile the narrative with the empirical class distribution.

2. The data availability statement should be clarified. The submission materials state that all data are fully available without restriction and that the data and code supporting the findings are publicly available in a GitHub repository. However, the repository description indicates that the original dataset cannot be publicly released and that only preprocessed train and test data are provided. The authors should specify precisely what is available, what is restricted, and whether the shared materials are sufficient for independent reproduction of the reported analyses.

3. The reporting of follow-up timing appears internally inconsistent and should be clarified. Table 1 reports a mean follow-up duration of 1.53 years and exactly 5.0 visual field tests per eye-episode, whereas Table 2 reports the interval between visual field examinations as approximately 1.93 years in both training and test sets. As currently labeled, these quantities do not appear to be compatible and may reflect a unit error, mislabeled variable, or reporting inconsistency.

4. There appears to be an incorrect table citation in the Methods section. In the description of the Wiggs-based criteria, the manuscript refers to Table 3, whereas the Wiggs-related counts appear in Table 5. This should be corrected for clarity.

5. Please clarify the decision threshold used for the reported sensitivity, specificity, precision, and F1 score values. The manuscript refers to a fixed decision threshold, but the threshold itself is not clearly specified.

6. Please provide complete implementation details for all models, especially XGBoost, including package versions and key hyperparameters.

7. The preprocessing workflow would be easier to evaluate if the manuscript quantified how many eyes, segments, or episodes were excluded at each filtering step.

8. Table 2's independent t-tests comparing the random train and test splits are not especially informative. It would be more useful to report class balance, center balance, and disease severity balance across the two splits.

9. The limitation that the Consensus label reflects only functional progression and is not linked to structural OCT evidence is important and should be emphasized clearly, potentially earlier in the manuscript.

10. The description of the regional partition used for the Wiggs label is not fully clear. The Methods state that pattern deviation plots were divided into six peripheral regions and two paracentral regions, but the parenthetical description appears to name only superior and inferior nasal step and superior and inferior Bjerrum areas. Please describe the regional partition unambiguously.

11. The Discussion states that the models were compared on the same dataset and architecture with "only the labeling strategy varied." This wording appears somewhat too strong, because the positive class prevalence also differs between the Consensus and Wiggs labels. The authors should revise this sentence to reflect that the comparison changes not only label definition but also class balance and likely the difficulty of the classification task.

Overall assessment

This is a potentially valuable study with an important question, a multicenter design, and a sizable curated dataset. The manuscript's central idea, that labeling strategy itself can strongly influence machine learning results, is worthwhile. However, the current version overstates what can be concluded from the present design. The most important issues are the framing of the task as prediction rather than within-window classification, overlap between labels and predictors, unresolved risk of patient- or eye-level leakage, inconsistency in feature reporting, limited support for multicenter generalizability claims, and the need for clearer statistical reporting. For these reasons, I recommend major revision.
