# Supplementary material for: Labeling matters: A multicenter machine learning study on visual field progression in Glaucoma
Source: PLoS One. 2026 Aug 28;21(8):e0357179. doi: 10.1371/journal.pone.0357179 (PMC13524274; doi:10.1371/journal.pone.0357179)
Supplement: Supplementary file 2 [file pone.0357179.s015.pdf]

Comments to authors

Recommendation: Major revision

The revised manuscript has improved substantially, and I appreciate the authors' detailed responses and additional analyses. In particular, the authors have clarified the retrospective classification nature of the study, added patient-level split sensitivity analysis, added leave-one-center-out validation, expanded the limitation regarding the absence of structural OCT validation, clarified the interpretation of the Wiggs' label, and improved the reporting of preprocessing and implementation details. These changes address several important concerns raised in the previous review.

However, I do not recommend acceptance in the present form. Several important issues remain unresolved, particularly regarding predictor reporting, feature construction, data availability, reproducibility, and interpretation of the algorithm-derived labels. These issues should be addressed before the manuscript can be considered acceptable.

Major comments

1. The predictor set is still not reported consistently: The Methods state that 10 machine-learning input features were used, including the mean and standard deviation of TDV and PDV. However, Table 2 still reports only Mean 52 TDV and Mean 52 PDV and does not explicitly report TDV standard deviation and PDV standard deviation as separate input features. Since these variables appear to be part of the classifier input set, Table 2 should be revised to include all 10 input features clearly, or the Methods should be corrected if these variables were not included in the final models.

2. The feature construction procedure requires clearer description: The Methods state that each eye-episode was divided into an early phase and a late phase, that mean values of 10 features were calculated separately for the two phases, and that the late-minus-early difference was used as the classifier input. However, this description is not fully clear for slope-based variables such as MD slope and VFI slope, which are usually calculated as trend estimates over time rather than simple early/late mean values. The authors should define exactly how each of the 10 features was computed, particularly MD slope, VFI slope, TDV standard deviation, and PDV standard deviation, and clarify whether each feature represents a late-minus-early difference, a full-episode slope, or another summary measure.

3. The data availability statement remains inconsistent: The authors' response explains that the complete raw multicenter clinical dataset cannot be publicly released because of ethical and institutional restrictions, while processed train/test data, sample data, and analysis code are available through GitHub. However, the submitted data availability information still indicates

that all data are fully available without restriction. This discrepancy should be corrected. The final data availability statement should clearly distinguish restricted raw multicenter clinical data from publicly available processed datasets, sample data, and analysis code.

4. The public repository should be updated and aligned with the revised manuscript: The authors should ensure that the shared code, notebooks, and outputs reproduce the revised tables and analyses as presented in the manuscript. If some parts of the preprocessing workflow cannot be reproduced because the raw clinical data are restricted, this limitation should be stated clearly in both the repository and the manuscript. The repository should also clearly document which parts of the revised analysis can be reproduced from the shared materials, including bootstrap confidence intervals, patient-level split analysis, leave-one-center-out validation, and any hyperparameter-tuning sensitivity analysis. Any outputs in the public repository should also be updated to match the corrected values in the revised manuscript.

5. The concern about overlap between label construction and predictor variables remains only partially resolved: The added correlation and ablation analyses are useful and improve the manuscript. However, the primary models still use features that are closely aligned with the label-defining criteria. Therefore, the reported high performance should not be interpreted as evidence of clinical ground-truth validity. The manuscript should state more clearly that the results primarily reflect the learnability and internal consistency of algorithm-derived functional labels within the selected feature space.

6. The ground-truth terminology and interpretation remain too strong in places: Phrases such as “optimized ground truth,” “reliable ground truth,” and “true glaucomatous progression” remain stronger than the study design supports, given the absence of independent expert adjudication, structural OCT validation, or prospective clinical outcome validation. These phrases should be replaced with more precise wording such as “algorithm-derived functional reference label” or “functional progression label.”

7. The term “Consensus label” remains potentially misleading: The proposed label is defined as progression when at least one of five algorithms identifies progression. This is an inclusive any-positive composite rule rather than a consensus rule in the usual sense of agreement among methods. Because the term “Consensus label” may imply stronger agreement or clinical validity than the definition supports, the authors should either rename the label, for example as an “inclusive composite algorithmic label” or “any-positive algorithmic label,” or state more explicitly throughout the manuscript that the term refers to an inclusive composite definition rather than agreement among algorithms.

8. The manuscript should clarify whether performance comparisons are descriptive or inferential: The authors added confidence intervals for several performance metrics, which is

helpful. However, the manuscript should clearly state whether AUC and average precision confidence intervals were calculated and where they are reported. In addition, if no formal statistical comparison between labeling strategies was performed, the comparison should be described as descriptive rather than inferential.

Minor comments

1. The Discussion still states that the models were compared with "only the labeling strategy varied." This wording should be revised because changing the labeling strategy also changes positive class prevalence and likely the difficulty of the classification task. A more accurate statement would be that the same dataset, input feature set, and model architectures were used, while label definition, class balance, and classification difficulty differed between the two labeling approaches.

2. The authors added class balance across the training and test sets, which is helpful. However, my previous comment also requested center balance and disease severity balance across the split. These should either be reported, preferably in Table 2 or a supplementary table, or the authors should explain why they are not available.

3. The decision threshold or default prediction rule used to calculate sensitivity, specificity, precision, and F1 score should be stated explicitly in the Methods section, not only in the response letter.

4. The authors state that a fixed random seed was used, but the exact seed value should be reported in the Methods or Supplementary Materials to improve reproducibility.

5. The authors should ensure that the repository language is consistent with the revised manuscript. Any statements suggesting that the model or labeling strategy supports fully generalizable clinical AI systems should be moderated unless supported by external validation beyond the included centers.

6. The Methods and supplementary materials should clearly identify which analyses were part of the primary revision and which were sensitivity analyses. This is particularly important for the patient-level split, leave-one-center-out validation, bootstrap confidence intervals, and any hyperparameter-tuning analysis.

7. The preprocessing flow has improved, but the authors should ensure that Supplementary Figure 1 reports the number of visual field tests, eyes, patients, segments, and eye-episodes retained or excluded at each major filtering step, including the segmentation and five-visit episode construction steps.

Overall assessment

The revised manuscript is scientifically improved and addresses several of the original methodological concerns. The added patient-level and center-level validation analyses are valuable, and the overall framing is more appropriate than in the original submission. However, the remaining issues are not only editorial. The inconsistent predictor reporting, unclear feature construction, unresolved data availability discrepancy, repository-manuscript mismatch, incomplete reproducibility documentation, and remaining over strong ground-truth language require further correction. I therefore recommend major revision before the manuscript can be considered for acceptance.
