# Supplementary material for: Labeling matters: A multicenter machine learning study on visual field progression in Glaucoma
Source: PLoS One. 2026 Aug 28;21(8):e0357179. doi: 10.1371/journal.pone.0357179 (PMC13524274; doi:10.1371/journal.pone.0357179)
Supplement: Supplementary file 3 [file pone.0357179.s003.docx]

**Supporting Table 1. Classification performance of machine learning models after excluding Advanced Glaucoma Intervention Study-related features.**

|  | Accuracy  (95% CI) | Sensitivity  (95% CI) | Specificity  (95% CI) | Precision  (95% CI) | F1 score  (95% CI) |
| --- | --- | --- | --- | --- | --- |
| SVM | 0.945  (0.935-0.955) | 0.821  (0.788-0.851) | 0.999  (0.996-1.00) | 0.996  (0.989-1.00) | 0.900  (0.879-0.917) |
| Random Forest | 0.953  (0.943-0.962) | 0.849  (0.818-0.877) | 0.998  (0.995-1.00) | 0.994  (0.986-1.00) | 0.916  (0.896-0.932) |
| Logistic Regression | 0.945  (0.935-0.955) | 0.817  (0.783-0.846) | 1.00 (1.00-1.00) | 1.00  (1.00-1.00) | 0.899  (0.879-0.917) |
| XGBoost | 0.949  (0.938-0.958) | 0.847  (0.815-0.875) | 0.992  (0.987-0.996) | 0.978  (0.964-0.990) | 0.908  (0.887-0.925) |

Nine features excluding Advanced Glaucoma Intervention Study score were used for model training and evaluation.
