# Supplementary material for: Labeling matters: A multicenter machine learning study on visual field progression in Glaucoma
Source: PLoS One. 2026 Aug 28;21(8):e0357179. doi: 10.1371/journal.pone.0357179 (PMC13524274; doi:10.1371/journal.pone.0357179)
Supplement: Supplementary file 4 [file pone.0357179.s004.docx]

**Supporting Table 2. Classification performance of machine learning models after excluding Collaborative Initial Glaucoma Treatment Study-related features.**

|  | Accuracy  (95% CI) | Sensitivity  (95% CI) | Specificity  (95% CI) | Precision  (95% CI) | F1 score  (95% CI) |
| --- | --- | --- | --- | --- | --- |
| SVM | 0.948  (0.937-0.957) | 0.830  (0.796-0.858) | 0.999  (0.996-1.00) | 0.996  (0.989-1.000) | 0.905  (0.885-0.922) |
| Random Forest | 0.955  (0.945-0.964) | 0.852  (0.821-0.880) | 0.999  (0.996-1.00) | 0.996  (0.990-1.000) | 0.919  (0.899-0.934) |
| Logistic Regression | 0.945  (0.935-0.955) | 0.817  (0.783-0.846) | 1.00  (1.00-1.00) | 1.000  (1.000-1.000) | 0.899  (0.879-0.917) |
| XGBoost | 0.951  (0.940-0.960) | 0.849  (0.817-0.876) | 0.994  (0.990-0.998) | 0.984  (0.971-0.994) | 0.911  (0.892-0.927) |

Nine features excluding Collaborative Initial Glaucoma Treatment Study score were used for model training and evaluation.
