# Supplementary material for: Labeling matters: A multicenter machine learning study on visual field progression in Glaucoma
Source: PLoS One. 2026 Aug 28;21(8):e0357179. doi: 10.1371/journal.pone.0357179 (PMC13524274; doi:10.1371/journal.pone.0357179)
Supplement: Supplementary file 5 [file pone.0357179.s005.docx]

**Supporting Table 3. Classification performance of machine learning models after excluding mean deviation-related features.**

|  | Accuracy  (95% CI) | Sensitivity  (95% CI) | Specificity  (95% CI) | Precision  (95% CI) | F1 score  (95% CI) |
| --- | --- | --- | --- | --- | --- |
| SVM | 0.897  (0.883-0.910) | 0.657  (0.617-0.694) | 0.999  (0.998-1.000) | 0.997  (0.992-1.000) | 0.792  (0.762-0.819) |
| Random Forest | 0.909  (0.896-0.921) | 0.710  (0.674-0.744) | 0.993  (0.989-0.997) | 0.978  (0.963-0.991) | 0.823  (0.798-0.847) |
| Logistic Regression | 0.895  (0.882-0.909) | 0.654  (0.614-0.692) | 0.999  (0.996-1.000) | 0.995  (0.986-1.000) | 0.789  (0.760-0.816) |
| XGBoost | 0.899  (0.884-0.912) | 0.714  (0.676-0.748) | 0.978  (0.969-0.985) | 0.931  (0.905-0.955) | 0.808  (0.781-0.834) |

Eight features excluding mean deviation and mean deviation slope were used for model training and evaluation.
