# Supplementary material for: Labeling matters: A multicenter machine learning study on visual field progression in Glaucoma
Source: PLoS One. 2026 Aug 28;21(8):e0357179. doi: 10.1371/journal.pone.0357179 (PMC13524274; doi:10.1371/journal.pone.0357179)
Supplement: Supplementary file 6 [file pone.0357179.s006.docx]

**Supporting Table 4. Classification performance of machine learning models after excluding Visual Field Index-related features.**

|  | Accuracy  (95% CI) | Sensitivity  (95% CI) | Specificity  (95% CI) | Precision  (95% CI) | F1 score  (95% CI) |
| --- | --- | --- | --- | --- | --- |
| SVM | 0.893  (0.879-0.906) | 0.643  (0.602-0.680) | 0.999  (0.998-1.000) | 0.997  (0.992-1.000) | 0.782  (0.751-0.809) |
| Random Forest | 0.885  (0.872-0.900) | 0.652  (0.612-0.687) | 0.985  (0.979-0.991) | 0.949  (0.926-0.970) | 0.773  (0.742-0.800) |
| Logistic Regression | 0.889  (0.875-0.902) | 0.636  (0.595-0.673) | 0.996  (0.993-0.999) | 0.986  (0.973-0.997) | 0.774  (0.742-0.800) |
| XGBoost | 0.884  (0.870-0.899) | 0.661  (0.620-0.696) | 0.980  (0.972-0.987) | 0.933  (0.907-0.956) | 0.774  (0.743-0.800) |

Eight features excluding Visual Field Index and Visual Field Index slope were used for model training and evaluation.
