# Supplementary material for: Labeling matters: A multicenter machine learning study on visual field progression in Glaucoma
Source: PLoS One. 2026 Aug 28;21(8):e0357179. doi: 10.1371/journal.pone.0357179 (PMC13524274; doi:10.1371/journal.pone.0357179)
Supplement: Supplementary file 7 [file pone.0357179.s007.docx]

**Supporting Table 5. Classification performance of machine learning models after excluding pointwise linear regression analysis-related features.**

|  | Accuracy  (95% CI) | Sensitivity  (95% CI) | Specificity  (95% CI) | Precision  (95% CI) | F1 score  (95% CI) |
| --- | --- | --- | --- | --- | --- |
| SVM | 0.947  (0.937-0.956) | 0.828  (0.793-0.857) | 0.999  (0.996-1.000) | 0.996  (0.989-1.000) | 0.904  (0.884-0.921) |
| Random Forest | 0.954  (0.944-0.963) | 0.851  (0.819-0.878) | 0.998  (0.995-1.000) | 0.994  (0.986-1.000) | 0.917  (0.897-0.933) |
| Logistic Regression | 0.945  (0.935-0.955) | 0.817  (0.783-0.846) | 1.000  (1.000-1.000) | 1.000  (1.000-1.000) | 0.899  (0.879-0.917) |
| XGBoost | 0.951  (0.941-0.960) | 0.847  (0.815-0.874) | 0.995  (0.991-0.998) | 0.986  (0.975-0.996) | 0.911  (0.891-0.927) |

Eight features excluding mean and standard deviation of total deviation value were used for model training and evaluation.
