# Supplementary material for: Labeling matters: A multicenter machine learning study on visual field progression in Glaucoma
Source: PLoS One. 2026 Aug 28;21(8):e0357179. doi: 10.1371/journal.pone.0357179 (PMC13524274; doi:10.1371/journal.pone.0357179)
Supplement: Supplementary file 8 [file pone.0357179.s008.docx]

**Supporting Table 6. Correlation between input features and progression labels (Consensus label and Wiggs’ label).**

|  | AGIS | CIGTS | MD | MD slope | VFI | VFI slope | TDV mean | TDV std | PDV mean | PDV std |
| --- | --- | --- | --- | --- | --- | --- | --- | --- | --- | --- |
| Consensus label | 0.26 | 0.25 | −0.32 | −0.50 | −0.29 | −0.49 | −0.31 | 0.22 | −0.23 | 0.22 |
| Wiggs’ label | 0.26 | 0.18 | −0.29 | −0.34 | −0.32 | −0.37 | −0.29 | 0.49 | −0.31 | 0.49 |
