# Supplementary material for: Labeling matters: A multicenter machine learning study on visual field progression in Glaucoma
Source: PLoS One. 2026 Aug 28;21(8):e0357179. doi: 10.1371/journal.pone.0357179 (PMC13524274; doi:10.1371/journal.pone.0357179)
Supplement: Supplementary file 9 [file pone.0357179.s009.docx]

**Supporting Table 7. Demographic and visual field–derived features used as machine learning inputs in the training and test sets for patient-level split analyses.** To prevent information leakage, the dataset was divided into training and test sets at the patient level. All eye-episodes from a given patient, including both eyes and repeated episodes from the same eye, were assigned exclusively to either the training or test set.

|  |  | Training data  (n = 7641) | Test data  (n = 1873) |
| --- | --- | --- | --- |
| Dataset characteristics | Age (year), mean $\pm$ SD | 56.738 ± 14.331 | 56.723 ± 14.555 |
|  | The intervals between visual field examinations (year), mean $\pm$ SD | 1.93 ± 0.54 | 1.938 ± 0.558 |
|  | Initial MD ≥ −6 dB | 7572 | 1850 |
|  | −6 dB > Initial MD ≥ −12 dB | 62 | 22 |
|  | −12 dB > Initial MD | 7 | 1 |
|  | Consensus label: stable / progressing, n (%) | 5365 (70.2%) / 2276 (29.8%) | 1302 (69.5%) / 571 (30.5%) |
|  | Wiggs’ label: stable / progressing, n (%) | 4846 (63.4%) / 2795 (36.6%) | 1171 (62.5%) / 702 (37.5%) |
|  | Center balance, n (%) | Dong-A : 3236 (42.4%),  Busan Paik : 949(12.4%),  Kosin : 253 (3.3%),  Pusan : 2669 (34.9%),  Yangsan : 534 (7.0%) | Dong-A : 810 (43.2%),  Busan Paik : 277(14.8%),  Kosin : 51 (2.7%),  Pusan : 624 (33.3%),  Yangsan : 111 (5.9%) |
| Features used for machine learning | AGIS, mean ± SD | 0.181 ± 1.728 | 0.17 ± 1.782 |
|  | CIGTS, mean ± SD | 0.012 ± 2.552 | -0.008 ± 2.57 |
|  | MD, mean ± SD | -0.148 ± 1.834 | -0.173 ± 1.953 |
|  | MD slope, mean ± SD | -0.137 ± 0.422 | -0.143 ± 0.426 |
|  | VFI, mean ± SD | -1.054 ± 5.293 | -0.966 ± 5.697 |
|  | VFI slope, mean ± SD | -0.462 ± 1.445 | -0.455 ± 1.461 |
|  | Mean 52 TDV mean, mean ± SD | -0.143 ± 1.877 | -0.167 ± 1.972 |
|  | Mean 52 TDV SD, mean ± SD | 2.501 ± 1.397 | 2.58 ± 1.467 |
|  | Mean 52 PDV mean, mean ± SD | -0.217 ± 1.147 | -0.203 ± 1.186 |
|  | Mean 52 PDV SD, mean ± SD | 2.501 ± 1.397 | 2.577 ± 1.469 |
