# Supplementary material for: Labeling matters: A multicenter machine learning study on visual field progression in Glaucoma
Source: PLoS One. 2026 Aug 28;21(8):e0357179. doi: 10.1371/journal.pone.0357179 (PMC13524274; doi:10.1371/journal.pone.0357179)
Supplement: Supplementary file 10 [file pone.0357179.s010.docx]

**Supporting Table 8. Classification performance of machine learning models for detecting visual field progression using the Consensus label with patient-level train–test splitting.**

|  | Accuracy  (95% CI) | Sensitivity  (95% CI) | Specificity  (95% CI) | Precision  (95% CI) | F1 score  (95% CI) |
| --- | --- | --- | --- | --- | --- |
| SVM | 0.939  (0.927-0.950) | 0.809  (0.776-0.840) | 0.996  (0.993-0.999) | 0.989  (0.980-0.998) | 0.890  (0.870-0.909) |
| RF | 0.942  (0.931-0.952) | 0.821  (0.790-0.852) | 0.995  (0.990-0.998) | 0.985  (0.973-0.996) | 0.896  (0.876-0.914) |
| LR | 0.935  (0.924-0.947) | 0.792  (0.758-0.824) | 0.998  (0.996-1.000) | 0.996  (0.989-1.000) | 0.882  (0.861-0.902) |
| XGBoost | 0.937  (0.926-0.948) | 0.813  (0.780-0.845) | 0.992  (0.987-0.996) | 0.977  (0.964-0.990) | 0.887  (0.867-0.906) |
