# Supplementary material for: Labeling matters: A multicenter machine learning study on visual field progression in Glaucoma
Source: PLoS One. 2026 Aug 28;21(8):e0357179. doi: 10.1371/journal.pone.0357179 (PMC13524274; doi:10.1371/journal.pone.0357179)
Supplement: Supplementary file 11 [file pone.0357179.s011.docx]

**Supporting Table 9. Classification performance of machine learning models for detecting visual field progression using the Wiggs’ label with patient-level train–test splitting.**

|  | Accuracy  (95% CI) | Sensitivity  (95% CI) | Specificity  (95% CI) | Precision  (95% CI) | F1 score  (95% CI) |
| --- | --- | --- | --- | --- | --- |
| SVM | 0.809  (0.791-0.826) | 0.651  (0.617-0.685) | 0.904  (0.887-0.919) | 0.802  (0.769-0.833) | 0.719  (0.691-0.747) |
| RF | 0.799  (0.781-0.816) | 0.675  (0.641-0.710) | 0.873  (0.855-0.890) | 0.761  (0.729-0.793) | 0.715  (0.688-0.741) |
| LR | 0.800  (0.782-0.818) | 0.613  (0.577-0.648) | 0.913  (0.897-0.927) | 0.808  (0.774-0.839) | 0.697  (0.667-0.726) |
| XGBoost | 0.799  (0.782-0.816) | 0.682  (0.650-0.716) | 0.869  (0.851-0.887) | 0.758  (0.725-0.789) | 0.718  (0.690-0.744) |
