# Supplementary material for: Labeling matters: A multicenter machine learning study on visual field progression in Glaucoma
Source: PLoS One. 2026 Aug 28;21(8):e0357179. doi: 10.1371/journal.pone.0357179 (PMC13524274; doi:10.1371/journal.pone.0357179)
Supplement: Supplementary file 12 [file pone.0357179.s012.docx]

**Supporting Table 10. Leave-one-center-out validation analysis.** Performance of machine learning models under leave-one-center-out validation. In each iteration, one center was held out for testing while models were trained using data from all remaining centers. Results demonstrate model performance and generalizability across participating institutions.

| Removed Center | Label | Model | **Accuracy** | **Recall** | **Specificity** | **Precision** | **F1** |
| --- | --- | --- | --- | --- | --- | --- | --- |
| Dong-A | Consensus | SVM | 0.949 (0.936-0.962) | 0.835 (0.793-0.877) | 0.996 (0.991-1.000) | 0.989 (0.975-1.000) | 0.906 (0.880-0.930) |
|  |  | RF | 0.956 (0.942-0.967) | 0.863 (0.822-0.900) | 0.994 (0.987-0.999) | 0.982 (0.965-0.996) | 0.919 (0.894-0.940) |
|  |  | LR | 0.944 (0.929-0.957) | 0.807 (0.762-0.850) | 1.000 (1.000-1.000) | 1.000 (1.000-1.000) | 0.893 (0.865-0.919) |
|  |  | XGBoost | 0.950 (0.937-0.963) | 0.860 (0.819-0.898) | 0.987 (0.978-0.995) | 0.965 (0.943-0.985) | 0.910 (0.884-0.932) |
|  | Wiggs’ | SVM | 0.795 (0.771-0.820) | 0.724 (0.685-0.764) | 0.852 (0.822-0.880) | 0.796 (0.759-0.834) | 0.759 (0.728-0.791) |
|  |  | RF | 0.800 (0.774-0.822) | 0.765 (0.727-0.802) | 0.827 (0.795-0.859) | 0.780 (0.743-0.817) | 0.772 (0.744-0.799) |
|  |  | LR | 0.791 (0.766-0.812) | 0.671 (0.629-0.711) | 0.886 (0.860-0.911) | 0.825 (0.788-0.861) | 0.740 (0.706-0.771) |
|  |  | XGBoost | 0.791 (0.766-0.813) | 0.753 (0.715-0.792) | 0.821 (0.790-0.849) | 0.770 (0.733-0.806) | 0.762 (0.731-0.791) |
| Busan Paik | Consensus | SVM | 0.959 (0.949-0.968) | 0.867 (0.837-0.895) | 0.997 (0.994-1.000) | 0.993 (0.984-1.000) | 0.926 (0.908-0.942) |
|  |  | RF | 0.964 (0.955-0.972) | 0.886 (0.857-0.912) | 0.996 (0.993-0.999) | 0.991 (0.981-0.998) | 0.935 (0.918-0.950) |
|  |  | LR | 0.960 (0.951-0.969) | 0.865 (0.835-0.894) | 1.000 (1.000-1.000) | 1.000 (1.000-1.000) | 0.928 (0.910-0.944) |
|  |  | XGBoost | 0.960 (0.950-0.970) | 0.890 (0.861-0.916) | 0.989 (0.983-0.995) | 0.972 (0.955-0.987) | 0.930 (0.912-0.945) |
|  | Wiggs’ | SVM | 0.824 (0.806-0.843) | 0.663 (0.625-0.703) | 0.915 (0.898-0.933) | 0.816 (0.781-0.851) | 0.732 (0.700-0.763) |
|  |  | RF | 0.812 (0.793-0.832) | 0.694 (0.656-0.733) | 0.878 (0.860-0.896) | 0.764 (0.727-0.798) | 0.727 (0.695-0.757) |
|  |  | LR | 0.808 (0.789-0.827) | 0.615 (0.574-0.655) | 0.917 (0.901-0.934) | 0.808 (0.773-0.846) | 0.699 (0.665-0.728) |
|  |  | XGBoost | 0.809 (0.791-0.828) | 0.692 (0.653-0.731) | 0.874 (0.854-0.896) | 0.758 (0.720-0.792) | 0.724 (0.693-0.753) |
| Kosin | Consensus | SVM | 0.948 (0.938-0.958) | 0.832 (0.803-0.862) | 0.999 (0.998-1.000) | 0.998 (0.993-1.000) | 0.908 (0.889-0.925) |
|  |  | RF | 0.959 (0.951-0.968) | 0.876 (0.849-0.901) | 0.996 (0.992-0.999) | 0.990 (0.981-0.998) | 0.929 (0.913-0.944) |
|  |  | LR | 0.949 (0.939-0.959) | 0.836 (0.806-0.866) | 0.999 (0.998-1.000) | 0.998 (0.993-1.000) | 0.910 (0.892-0.927) |
|  |  | XGBoost | 0.958 (0.948-0.967) | 0.876 (0.849-0.902) | 0.994 (0.989-0.998) | 0.984 (0.973-0.994) | 0.927 (0.911-0.943) |
|  | Wiggs’ | SVM | 0.817 (0.800-0.834) | 0.653 (0.620-0.685) | 0.916 (0.900-0.931) | 0.823 (0.791-0.852) | 0.728 (0.700-0.754) |
|  |  | RF | 0.819 (0.802-0.837) | 0.700 (0.668-0.732) | 0.891 (0.873-0.909) | 0.793 (0.760-0.826) | 0.744 (0.716-0.769) |
|  |  | LR | 0.814 (0.796-0.831) | 0.630 (0.595-0.665) | 0.924 (0.908-0.939) | 0.833 (0.799-0.863) | 0.717 (0.688-0.745) |
|  |  | XGBoost | 0.821 (0.803-0.837) | 0.696 (0.662-0.730) | 0.895 (0.878-0.913) | 0.799 (0.767-0.832) | 0.744 (0.716-0.770) |
| Pusan | Consensus | SVM | 0.938 (0.923-0.951) | 0.817 (0.778-0.854) | 1.000 (1.000-1.000) | 1.000 (1.000-1.000) | 0.899 (0.875-0.922) |
|  |  | RF | 0.951 (0.938-0.962) | 0.855 (0.816-0.888) | 1.000 (1.000-1.000) | 1.000 (1.000-1.000) | 0.922 (0.899-0.941) |
|  |  | LR | 0.937 (0.922-0.951) | 0.814 (0.775-0.853) | 1.000 (1.000-1.000) | 1.000 (1.000-1.000) | 0.898 (0.873-0.920) |
|  |  | XGBoost | 0.946 (0.932-0.959) | 0.860 (0.822-0.894) | 0.990 (0.984-0.996) | 0.978 (0.964-0.992) | 0.915 (0.893-0.936) |
|  | Wiggs’ | SVM | 0.812 (0.790-0.833) | 0.636 (0.591-0.682) | 0.906 (0.885-0.925) | 0.782 (0.739-0.824) | 0.701 (0.664-0.737) |
|  |  | Random_Forest | 0.815 (0.794-0.836) | 0.682 (0.638-0.726) | 0.885 (0.863-0.906) | 0.758 (0.713-0.802) | 0.718 (0.683-0.751) |
|  |  | Logistic_Regression | 0.804 (0.782-0.826) | 0.607 (0.564-0.656) | 0.909 (0.888-0.928) | 0.778 (0.734-0.822) | 0.682 (0.646-0.719) |
|  |  | XGBoost | 0.819 (0.797-0.838) | 0.692 (0.648-0.735) | 0.886 (0.864-0.906) | 0.763 (0.718-0.803) | 0.725 (0.688-0.761) |
| Yangsan | Consensus | SVM | 0.939 (0.928-0.949) | 0.802 (0.770-0.834) | 0.999 (0.998-1.000) | 0.998 (0.993-1.000) | 0.889 (0.869-0.909) |
|  |  | Random_Forest | 0.948 (0.937-0.958) | 0.834 (0.803-0.864) | 0.998 (0.994-1.000) | 0.993 (0.985-1.000) | 0.907 (0.888-0.925) |
|  |  | Logistic_Regression | 0.934 (0.923-0.945) | 0.786 (0.752-0.821) | 0.999 (0.998-1.000) | 0.998 (0.993-1.000) | 0.879 (0.857-0.901) |
|  |  | XGBoost | 0.945 (0.935-0.955) | 0.830 (0.798-0.860) | 0.996 (0.992-0.999) | 0.989 (0.979-0.998) | 0.903 (0.883-0.921) |
|  | Wiggs’ | SVM | 0.834 (0.817-0.853) | 0.671 (0.637-0.711) | 0.924 (0.909-0.939) | 0.832 (0.800-0.864) | 0.743 (0.716-0.771) |
|  |  | Random_Forest | 0.825 (0.807-0.842) | 0.727 (0.694-0.762) | 0.880 (0.860-0.898) | 0.771 (0.737-0.802) | 0.748 (0.722-0.775) |
|  |  | Logistic_Regression | 0.824 (0.807-0.844) | 0.641 (0.604-0.682) | 0.926 (0.910-0.942) | 0.829 (0.797-0.860) | 0.723 (0.694-0.754) |
|  |  | XGBoost | 0.819 (0.801-0.837) | 0.717 (0.685-0.752) | 0.875 (0.857-0.895) | 0.762 (0.730-0.796) | 0.739 (0.713-0.767) |
