# Supplementary material for: Labeling matters: A multicenter machine learning study on visual field progression in Glaucoma
Source: PLoS One. 2026 Aug 28;21(8):e0357179. doi: 10.1371/journal.pone.0357179 (PMC13524274; doi:10.1371/journal.pone.0357179)
Supplement: Supplementary file 13 [file pone.0357179.s016.docx]

**Reviewer #1:** 

**Major comments**

1. The study is framed as prediction, but the present design appears closer to retrospective within-window classification: The unit of analysis is a 5-visit eye-episode. The labels are defined within that same episode, and the model inputs are also derived from the same episode by comparing the first two visits with the last three visits. In particular, the Wiggs label explicitly uses the final visit or final two visits in its definition. Because late-episode information contributes both to the target and to the predictor set, the present design appears to support retrospective classification of already observed progression within a fixed window more than true future prediction. Accordingly, claims such as “early progression detection” and “early warning systems” should be moderated unless the authors can demonstrate a strictly forward-looking prediction framework.

Response: We thank the reviewer for this important methodological observation. We agree that the terms "early progression detection" and "early warning systems" imply a prospective, forward-looking prediction framework, which is not what the present study design supports. Our study is retrospective in nature: the machine learning models were trained and evaluated on labeled eye-episodes in which all five visual field examinations within each episode were available at the time of labeling. Accordingly, the models classify whether progression occurred within a completed series of observations, rather than predicting future progression from an incomplete or early time series.

In response, we have revised the relevant sentences in the Discussion section to more accurately reflect the retrospective, classification-based nature of our framework. Specifically, "early progression detection" has been replaced with "sensitive identification of progression," and "early warning systems" has been removed.

2. There is substantial overlap between label construction and model inputs, raising a serious concern about circularity: The Consensus label is defined by AGIS, CIGTS, MD slope, VFI slope, and PLR. However, the model inputs also include AGIS, CIGTS, MD, MD slope, VFI, VFI slope, and TDV/PDV-based summary variables. This means that the classifiers are trained using predictors that substantially overlap with the target definition itself. The ablation analysis is useful, but it does not fully resolve the core concern because the principal reported model performances still rely on overlapping labels and features. At minimum, this issue should be discussed more explicitly. Ideally, the authors should provide a sensitivity analysis using features that are independent of the criteria used to define the labels.

Response: As the reviewer appropriately pointed out, there is both a conceptual and statistical relationship between the criteria used to construct the Consensus label and some of the model input variables. Specifically, the Consensus label was defined based on rule-based progression indicators, including AGIS_test, CIGTS_test, MD_slope_test, VFI_slope_test, and PLR_test, whereas the model input variables included AGIS, CIGTS, MD, VFI, MD slope value, VFI slope value, and TDV/PDV-derived summary metrics. Therefore, it is possible that a portion of the model performance reflects information that is also related to the label definition.

However, we would like to emphasize that the label-defining variables and predictor variables used in this study were not direct duplicates of the same variables. The Consensus label was defined using binary rule-based indicators that determine whether specific progression criteria were met, whereas the model inputs consisted of continuous numerical values or summary statistics. For example, AGIS_test is a binary indicator that evaluates whether a predefined level of change from baseline has occurred consistently over time, whereas AGIS refers to the raw score itself at each visit. Similarly, MD_slope_test and VFI_slope_test are binary outcome variables defined according to progression criteria that incorporate statistical significance, whereas the predictor variables MD slope and VFI slope values represent the continuous magnitude of change itself. Therefore, we considered the structure of our study to be closer to the use of progression-related clinical measures in different forms, rather than a case of direct label leakage resulting from the reuse of identical variables.

In addition, the objective of this study was not to construct a completely independent prediction model after excluding all progression-related features. Rather, our aim was to investigate how different labeling strategies influence machine learning performance when clinically relevant progression descriptors are used as model inputs. Therefore, the inclusion of progression-related clinical variables as features was an intentional aspect of the study design.

Nevertheless, we agree that the possibility of circularity raised by the reviewer represents an important methodological consideration. To further evaluate this issue, we performed an additional ablation analysis (included in the Supplementary Materials) in which feature groups associated with individual progression criteria were removed one at a time, and model performance was re-evaluated. The results showed that the overall performance patterns were largely preserved despite the removal of specific feature groups, suggesting that the observed performance cannot be explained solely by a single progression criterion or simple one-to-one feature duplication.

Furthermore, not only the Consensus label but also the Wiggs’ label demonstrated correlations with progression-related variables, and several spatial variability-related features showed even stronger correlations with the Wiggs’ label. This suggests that the association between progression-related clinical descriptors and the labels is not unique to a particular labeling strategy but rather reflects a broader structural characteristic shared across different definitions of glaucoma progression. Therefore, we believe that the observed model performance is better explained by the integration of multiple progression-related patterns than by direct feature duplication alone. Accordingly, we have added a table reporting the correlations between each feature and both the Consensus and Wiggs’ labels as shown below as Supplementary Table 6.

Supplementary Table 6. Correlation between input features and progression labels (Consensus label and Wiggs’ label)

|  | AGIS | CIGTS | MD | MD slope | VFI | VFI slope | TDV mean | TDV SD | PDV mean | PDV SD |
| --- | --- | --- | --- | --- | --- | --- | --- | --- | --- | --- |
| Consensus label | 0.26 | 0.25 | −0.32 | −0.50 | −0.29 | −0.49 | −0.31 | 0.22 | −0.23 | 0.22 |
| Wiggs’ label | 0.26 | 0.18 | −0.29 | −0.34 | −0.32 | −0.37 | −0.29 | 0.49 | −0.31 | 0.49 |

We have added following sentences in the Results section.

"To further examine the relationship between input features and progression labels, and to address the potential concern that model performance under the Consensus label may partly reflect overlap between label-defining criteria and predictor variables, we calculated point-biserial correlation coefficients between each continuous input feature and both progression labels (Supplementary Table 6). Although the Consensus label showed stronger correlations with global trend-based features such as MD slope (r = −0.50) and VFI slope (r = −0.49), consistent with its algorithmic definition, the Wiggs' label demonstrated comparatively stronger associations with spatial variability features, particularly TDV standard deviation (r = 0.49) and PDV standard deviation (r = 0.49). This divergence in feature-label correlation patterns indicates that each label captures a distinct and internally coherent dimension of VF deterioration. Importantly, the performance differences observed between the two labeling strategies are therefore not solely attributable to direct overlap between input features and label-defining criteria, but rather reflect the degree to which each label is aligned with the overall feature space available in this study. The lower predictive performance observed under the Wiggs' label likely reflects the relative underrepresentation of spatially granular features in the current input set, rather than the clinical inferiority of the label itself."

3. The manuscript should clarify the train-test split in enough detail to rule out patient-level or eye-level leakage: The paper states that the unit of analysis is the eye-episode, that 9,514 eye-episodes from 4,496 patients were analyzed, and that 80% of eye-episodes were assigned to training and 20% to testing. However, it is not stated whether the split was grouped by patient or by eye. If multiple episodes from the same eye, or both eyes from the same patient, were allowed to appear in both training and test sets, the reported performance could be optimistic. The authors should clarify this explicitly and, preferably, provide a grouped patient-level split as a sensitivity analysis.

Response: We thank the reviewer for this important comment. As described in the original manuscript, 80% of all eye-episodes were used as the training dataset and the remaining 20% were used as the test dataset (“Of all eye-episodes, 80% were used as the training dataset and the remaining 20% were used as the test dataset.”). Because the present study was conducted at the eye-episode level, multiple eye-episodes originating from the same patient or eye could potentially be included in both the training and test datasets. This may introduce correlated information between the two datasets and could lead to optimistic estimates of model performance.

However, the primary objective of this study was not to develop a strictly patient-independent prediction model, but rather to compare how different labeling strategies influence the performance of machine learning models in learning longitudinal progression patterns. In addition, glaucomatous progression may manifest as heterogeneous longitudinal progression patterns over time, even within the same patient. Therefore, we considered eye-episode–level analysis to be appropriate for capturing a broader range of progression dynamics.

Nevertheless, to evaluate the potential impact raised by the reviewer, we performed an additional sensitivity analysis using a patient-level split, and the results have been provided in Supplementary Tables 7-9. After ensuring that all eye-episodes originating from the same patient were assigned exclusively to either the training or test dataset, the relative performance trends across labeling strategies and the main study conclusions remained consistent. These findings suggest that the primary observations of this study are not solely dependent on eye-episode–level data partitioning and that the comparative results of the proposed labeling strategies are relatively robust.

Supplementary Table 7. To prevent information leakage, train–test splitting was performed at the patient level using GroupShuffleSplit. All eye-episodes originating from the same patient, including both eyes and multiple episodes from the same eye, were assigned exclusively to either the training or test set.

|  |  | Training data (n=7641) | Test data  (n=1873) |
| --- | --- | --- | --- |
| Dataset characteristics | Age (year), mean $\pm$ SD | 56.738 ± 14.331 | 56.723 ± 14.555 |
|  | The intervals between visual field examinations (year), mean $\pm$ SD | 1.93 ± 0.54 | 1.938 ± 0.558 |
|  | Consensus label: stable / progressing, n (%) | 5365 (70.2%) / 2276 (29.8%) | 1302 (69.5%) / 571 (30.5%) |
|  | Wiggs’ label: stable / progressing, n (%) | 4846 (63.4%) / 2795 (36.6%) | 1171 (62.5%) / 702 (37.5%) |
| Features used for Machine Learning | AGIS, mean ± SD | 0.181 ± 1.728 | 0.17 ± 1.782 |
|  | CIGTS, mean ± SD | 0.012 ± 2.552 | -0.008 ± 2.57 |
|  | MD, mean ± SD | -0.148 ± 1.834 | -0.173 ± 1.953 |
|  | MD slope, mean ± SD | -0.137 ± 0.422 | -0.143 ± 0.426 |
|  | VFI, mean ± SD | -1.054 ± 5.293 | -0.966 ± 5.697 |
|  | VFI slope, mean ± SD | -0.462 ± 1.445 | -0.455 ± 1.461 |
|  | Mean 52 TDV, mean ± SD | -0.143 ± 1.877 | -0.167 ± 1.972 |
|  | Mean 52 PDV, mean ± SD | -0.217 ± 1.147 | -0.203 ± 1.186 |

Supplementary Table 8. Classification performance of machine learning models for detecting visual field progression using the Consensus label with patient-level train–test splitting

|  | Accuracy  (95% CI) | Sensitivity  (95% CI) | Specificity  (95% CI) | Precision  (95% CI) | F1 score  (95% CI) |
| --- | --- | --- | --- | --- | --- |
| SVM | 0.939  (0.928-0.950) | 0.809  (0.777-0.841) | 0.996  (0.992-0.999) | 0.989  (0.979-0.998) | 0.890  (0.869-0.909) |
| RF | 0.941  (0.929-0.952) | 0.823  (0.792-0.855) | 0.993  (0.988-0.997) | 0.981  (0.968-0.993) | 0.895  (0.875-0.913) |
| LR | 0.935  (0.923-0.946) | 0.792  (0.760-0.824) | 0.998  (0.996-1.000) | 0.996  (0.989-1.000) | 0.882  (0.860-0.902) |
| XGBoost | 0.937  (0.926-0.947) | 0.813  (0.780-0.845) | 0.992 (0.987-0.996) | 0.977  (0.963-0.989) | 0.887  (0.863-0.906) |

Supplementary Table 9. Classification performance of machine learning models for detecting visual field progression using the Wiggs’ label with patient-level train–test splitting

|  | Accuracy  (95% CI) | Sensitivity  (95% CI) | Specificity  (95% CI) | Precision  (95% CI) | F1 score  (95% CI) |
| --- | --- | --- | --- | --- | --- |
| SVM | 0.809  (0.791-0.826) | 0.651  (0.618-0.688) | 0.904  (0.886-0.921) | 0.802  (0.770-0.835) | 0.719  (0.691-0.747) |
| RF | 0.805  (0.788-0.821) | 0.687  (0.653-0.721) | 0.875  (0.855-0.894) | 0.768  (0.734-0.800) | 0.725  (0.697-0.752) |
| LR | 0.800  (0.784-0.818) | 0.613  (0.575-0.649) | 0.913  (0.897-0.929) | 0.808  (0.772-0.840) | 0.697  (0.669-0.724) |
| XGBoost | 0.799  (0.780-0.816) | 0.682  (0.648-0.715) | 0.869  (0.850-0.889) | 0.758  (0.726-0.793) | 0.718  (0.692-0.744) |

4. The multicenter nature of the dataset is a strength, but the current validation strategy does not fully support the manuscript’s generalizability claims: The manuscript emphasizes heterogeneous clinical settings and concludes that the proposed labeling strategy supports robust and generalizable AI systems. However, the Methods describe a single 80/20 split rather than center-held-out validation or a leave-one-center-out design. If data from the same institutions appear in both training and test sets, institution-specific patterns may be shared across splits. A center-level validation strategy would provide much stronger support for the generalizability claims, or alternatively the wording in the Abstract and Discussion should be moderated.

Response: We thank the reviewer for this important comment. we have moderated the wording in the Abstract to avoid overstating the generalizability of the proposed framework.

Specifically, the sentence: “These findings underscore that careful definition of ground truth is critical for developing robust and generalizable artificial intelligence systems for glaucoma.” has been revised to: “These findings underscore that careful definition of ground truth is critical for developing reliable artificial intelligence systems for glaucoma progression assessment.”

In addition, the Discussion statement: “Strict inclusion criteria regarding test intervals, reliability indices, and temporal continuity minimized noise-driven variability and enhanced the generalizability of the models.” has been revised to “Strict inclusion criteria regarding test intervals, reliability indices, and temporal continuity minimized noise-driven variability and improved data quality.”

These revisions more appropriately reflect the scope of the present study while avoiding claims of external generalizability that were not directly evaluated.

5. The reporting of the predictor set is internally inconsistent and should be corrected: The Methods state that the model uses 10 features, including the mean and standard deviation of TDV and PDV. However, Table 2 lists age, interval between examinations, AGIS, CIGTS, MD, MD slope, VFI, VFI slope, Mean 52 TDV, and Mean 52 PDV, but does not show the standard deviation of TDV or the standard deviation of PDV. In addition, age and interval are described in Table 2 as machine learning inputs, yet they are not included in the explicit list of 10 features in the Methods. The authors should reconcile the Methods and Table 2 so that the exact predictor set is unambiguous.

Response: We agree with the reviewer that the description may have caused confusion. The classifier input features consisted of the following 10 variables: AGIS, CIGTS, MD, MD slope, VFI, VFI slope, TDV mean, TDV standard deviation, PDV mean, and PDV standard deviation.

And the notation “mean ± SD” referred to the descriptive statistics of each variable rather that the TDV/PDV standard deviation features used as classifier inputs. As a result, the TDV/PDV standard deviation predictor variables were not explicitly distinguished in the table, which may have caused ambiguity regarding the exact predictor set.

In contrast, age and interval between VF examinations were presented as descriptive variables to summarize the characteristics of the dataset and were not used as classifier inputs. In addition, the original Table 2 was intended to present descriptive statistics of the training and test datasets rather than to define the exact predictor set used for machine learning.

Accordingly, we revised Table 2 to clearly distinguish descriptive variables from classifier input features and to explicitly label the TDV/PDV standard deviation variables.

Table 2. Demographic and visual field–derived features used as machine learning inputs in the training and test sets. Age and the interval between visual field examinations are reported as the mean and standard deviation for each eye-episode, whereas all other variables are presented as the mean and standard deviation of the change values, calculated as the difference between the late-phase and early-phase averages within each eye-episode.

|  |  | Training data (n=7611) | Test data  (n=1903) |
| --- | --- | --- | --- |
| Dataset characteristics | Age (year), mean $\pm$ SD | 56.755 ± 14.484 | 56.656 ± 13.932 |
|  | The intervals between visual field examinations (year), mean $\pm$ SD | 1.933 ± 0.546 | 1.924 ± 0.536 |
|  | Consensus label: stable / progressing, n (%) | 5333 (70.1%) / 2278 (29.9%) | 1334 (70.1%) / 569 (29.9%) |
|  | Wiggs’ label: stable / progressing, n (%) | 4814 (63.3%) / 2797 (36.7%) | 1203 (63.2%) / 700 (36.8%) |
| Features used for Machine Learning | AGIS, mean $\pm$ SD | 0.179 ± 1.744 | 0.176 ± 1.718 |
|  | CIGTS, mean $\pm$ SD | 0.012 ± 2.561 | −0.006 ± 2.533 |
|  | MD, mean $\pm$ SD | −0.154 ± 1.87 | −0.149 ± 1.81 |
|  | MD slope, mean $\pm$ SD | −0.14 ± 0.429 | −0.129 ± 0.394 |
|  | VFI, mean $\pm$ SD | −1.026 ± 5.408 | −1.08 ± 5.239 |
|  | VFI slope, mean $\pm$ SD | −0.469 ± 1.466 | −0.427 ± 1.373 |
|  | Mean 52 TDV, mean $\pm$ SD | −0.149 ± 1.905 | −0.143 ± 1.861 |
|  | Mean 52 PDV, mean $\pm$ SD | −0.21 ± 1.16 | −0.231 ± 1.133 |

6. The statistical reporting should be strengthened with uncertainty estimates and clearer comparison of labeling strategies: The paper reports point estimates for accuracy, sensitivity, specificity, precision, F1 score, AUC, and average precision, but I did not see confidence intervals, repeated resampling, or formal statistical comparisons between models or between the two labeling strategies. Because the central claim is that label definition materially affects model performance, uncertainty around these differences should be quantified. Bootstrapped confidence intervals and appropriate comparisons for AUC and threshold-based metrics would strengthen the manuscript considerably.

Response: We thank the reviewer for this valuable suggestion regarding statistical reporting and uncertainty estimation. In response, we additionally estimated 95% confidence intervals for all classification performance metrics, including accuracy, sensitivity, specificity, precision, F1 score, AUC, and average precision, using 1000 bootstrap resamples. The corresponding confidence intervals have been added to the revised tables.

In addition, the ROC curve figures were revised to incorporate the confidence interval information for AUC estimates. These revisions provide a more robust assessment of model performance and variability across labeling strategies.

Table 4. Classification performance of machine learning models for detecting visual field progression using the Consensus label

|  | Accuracy  (95% CI) | Sensitivity  (95% CI) | Specificity  (95% CI) | Precision  (95% CI) | F1 score  (95% CI) |
| --- | --- | --- | --- | --- | --- |
| SVM | 0.947  (0.936-0.956) | 0.824  (0.790-0.853) | 0.999  (0.998-1.000) | 0.998  (0.993-1.000) | 0.903  (0.882-0.920) |
| RF | 0.955  (0.944-0.964) | 0.852  (0.821-0.879) | 0.999  (0.996-1.000) | 0.996  (0.990-1.000) | 0.919  (0.899-0.934) |
| LR | 0.945  (0.935-0.955) | 0.817  (0.783-0.846) | 1.000  (1.000-1.000) | 1.000  (1.000-1.000) | 0.899  (0.879-0.917) |
| XGBoost | 0.951  (0.941-0.960) | 0.849  (0.817-0.877) | 0.995  (0.991-0.998) | 0.986  (0.974-0.996) | 0.912  (0.891-0.929) |

CI, Confidence Interval; SVM, Support Vector Machine; RF, Random Forest; LR, Logistic Regression

Table 6. Classification performance of machine learning models for detecting visual field progression using the Wiggs’ label.

|  | Accuracy  (95% CI) | Sensitivity  (95% CI) | Specificity  (95% CI) | Precision  (95% CI) | F1 score  (95% CI) |
| --- | --- | --- | --- | --- | --- |
| SVM | 0.826  (0.809-0.843) | 0.660  (0.624-0.694) | 0.922  (0.907-0.936) | 0.831  (0.802-0.859) | 0.736  (0.707-0.761) |
| RF | 0.828  (0.811-0.844) | 0.717  (0.683-0.747) | 0.892  (0.875-0.910) | 0.794  (0.766-0.825) | 0.754  (0.728-0.776) |
| LR | 0.811  (0.794-0.827) | 0.630  (0.594-0.665) | 0.916  (0.901-0.932) | 0.814  (0.782-0.845) | 0.710  (0.683-0.737) |
| XGBoost | 0.799  (0.780-0.816) | 0.681  (0.647-0.716) | 0.867  (0.848-0.888) | 0.749  (0.715-0.782) | 0.714  (0.685-0.739) |

CI, Confidence Interval; SVM, Support Vector Machine; RF, Random Forest; LR, Logistic Regression

7. The Introduction is generally clear, but several framing claims should be moderated or better justified: The Introduction appropriately summarizes the problem of low agreement among visual field progression algorithms and motivates the study question. However, the novelty claim that no previous study has systematically compared how labeling strategy affects machine learning performance should be phrased more cautiously, especially because the manuscript itself cites prior consensus-based work. In addition, the rationale for defining the proposed Consensus label as positivity under any one of five criteria is underdeveloped, particularly given that the cited prior consensus-based approach used a much stricter threshold. Finally, the phrase “optimized ground truth” appears stronger than the present study design can support, because the labels are not externally validated against structural OCT, expert adjudication, or long-term clinical outcomes.

Response: Despite the clinical importance of detecting glaucomatous visual field (VF) progression, substantial disagreement exists among commonly used progression algorithms. Each algorithm, including the Advanced Glaucoma Intervention Study (AGIS) score, the Collaborative Initial Glaucoma Treatment Study (CIGTS) score, mean deviation (MD) slope, visual field index (VFI) slope, and pointwise linear regression (PLR), captures distinct aspects of VF deterioration through different analytical frameworks, and no single method has demonstrated consistently superior performance across all clinical scenarios **[1, 2]**.

Indeed, significant discordance has been reported among these traditional algorithms, with only a small proportion of eyes classified identically across all methods **[2-5]**. Furthermore, relying solely on any single algorithm carries an inherent risk of false-negative classification, as each method may miss progression signals that others detect **[6]**. This variability poses a fundamental challenge when constructing labeled datasets for supervised machine learning, as the choice of labeling strategy directly determines what the model learns to detect.

To address this challenge, we proposed a Consensus label defined as positive when at least one of the five algorithms detected progression. This "any-positive" strategy was adopted based on both clinical and methodological rationale. Glaucoma is the leading cause of irreversible blindness worldwide, and because visual loss once incurred cannot be restored, early detection and timely intervention are paramount **[7]**. Given this irreversibility, the clinical consequence of a missed progression event (false negative) far outweighs the cost of a false-positive alert. Since each algorithm may detect progression signals that others miss, particularly given that different methods are sensitive to different patterns of VF loss, requiring agreement among multiple algorithms before assigning a positive label risks systematically discarding genuine progression signals present in the training data. The any-positive Consensus label was therefore designed to maximize sensitivity in label assignment, ensuring that eyes with any algorithm-detectable signal of deterioration are retained as positive cases, consistent with the clinical principle that any indicator of progression in an irreversible disease warrants heightened vigilance.

**[1]** Dixit A, Yohannan J, Boland MV. Assessing Glaucoma Progression Using Machine Learning Trained on Longitudinal Visual Field and Clinical Data. Ophthalmology. 2021;128(7):1016-1026. doi:10.1016/j.ophtha.2020.12.020

**[2]** Rabiolo A, Morales E, Mohamed L, et al. Comparison of Methods to Detect and Measure Glaucomatous Visual Field Progression. Transl Vis Sci Technol. 2019;8(5):2. doi:10.1167/tvst.8.5.2

**[3]** Antón A, Pazos M, Martín B, Navero JM, Ayala ME, Castany M, Martínez P, Bardavío J. Glaucoma progression detection: agreement, sensitivity, and specificity of expert visual field evaluation, event analysis, and trend analysis. Eur J Ophthalmol. 2013 Mar-Apr;23(2):187-95. doi: 10.5301/ejo.5000193. PMID: 23065852.

[4] Rao HL, Kumbar T, Kumar AU, Babu JG, Senthil S, Garudadri CS. Agreement between event-based and trend-based glaucoma progression analyses. Eye (Lond). 2013 Jul;27(7):803-8. doi: 10.1038/eye.2013.77. Epub 2013 Apr 19. PMID: 23598668; PMCID: PMC3709397.

[5] Wu Z, Medeiros FA. Comparison of Visual Field Point-Wise Event-Based and Global Trend-Based Analysis for Detecting Glaucomatous Progression. Transl Vis Sci Technol. 2018 Aug 27;7(4):20. doi: 10.1167/tvst.7.4.20. PMID: 30159212; PMCID: PMC6111742.

**[6]** Tsamis E, La Bruna S, Rai A, Leshno A, Grossman J, Cioffi G, Liebmann JM, De Moraes CG, Hood DC. Progression of Early Glaucomatous Damage: Performance of Summary Statistics From Optical Coherence Tomography and Perimetry. Transl Vis Sci Technol. 2023 Mar 1;12(3):19. doi: 10.1167/tvst.12.3.19. PMID: 36939711; PMCID: PMC10043504.

**[7]** Tham YC, Li X, Wong TY, Quigley HA, Aung T, Cheng CY. Global prevalence of glaucoma and projections of glaucoma burden through 2040: a systematic review and meta-analysis. Ophthalmology. 2014;121(11):2081–2090. **PMID: 24974815**

We have added following sentences in the Introduction section.

“Furthermore, relying solely on any single algorithm carries an inherent risk of false-negative classification, as each method may miss progression signals that others detect.^10^ This variability poses a fundamental challenge when constructing labeled datasets for supervised machine learning, as the choice of labeling strategy directly determines what the model learns to detect.”

“To address this challenge, we proposed a “Consensus label” defined as positive when at least one of the five algorithms detected progression. This "any-positive" strategy was adopted based on both clinical and methodological rationale. Glaucoma is the leading cause of irreversible blindness worldwide, and because visual loss once incurred cannot be restored, early detection and timely intervention are paramount.^11^ Given this irreversibility, the clinical consequence of a missed progression event (false negative) far outweighs the cost of a false-positive alert. Since each algorithm may detect progression signals that others miss, particularly given that different methods are sensitive to different patterns of VF loss, requiring agreement among multiple algorithms before assigning a positive label risks systematically discarding genuine progression signals present in the training data. The any-positive Consensus label was therefore designed to maximize sensitivity in label assignment, ensuring that eyes with any algorithm-detectable signal of deterioration are retained as positive cases, consistent with the clinical principle that any indicator of progression in an irreversible disease warrants heightened vigilance.”

In addition, the sentence: “However, no previous study has systematically compared how the choice of labeling strategy (i.e., definition of progression) affects the performance of machine learning models in detecting VF progression.” has been revised to: “However, to our knowledge, prior studies have not directly and systematically compared how the choice of labeling strategy, independent of model architecture or dataset, affects machine learning performance in VF progression detection, and the relative impact of labeling definition on model learning remains poorly characterized.”.

**Minor comments**

1. The interpretation of the Wiggs label as “stricter” or more “conservative” requires clearer explanation, because its observed positive rate is higher than that of the Consensus label. This may be explainable, but it is counterintuitive and should be discussed explicitly so that readers can reconcile the narrative with the empirical class distribution.

Response: We thank the reviewer for this important comment. As the reviewer correctly noted, describing the Wiggs’ label as “stricter” or more “conservative” may appear counterintuitive because its progression-positive rate was higher than that of the Consensus label.

In this study, however, the term “conservative” refers not to the overall frequency of progression-positive cases, but rather to the nature of the progression criteria themselves. The Wiggs’ label is based on a region-level rule requiring spatial consistency and repeated confirmation of visual field defects, which makes it a more conservative and structured definition of progression.

At the same time, the Wiggs’ label includes event-based criteria such as PDV-based regional worsening and the appearance of new regional defects. These criteria can identify localized spatial changes that may not be captured by global trend-based algorithms such as MD slope or VFI slope. As a result, the Wiggs’ label may classify a larger number of eye-episodes as progression despite its more conservative progression framework.

We have clarified this point in the revised Discussion section to avoid potential confusion for readers.

2. The data availability statement should be clarified. The submission materials state that all data are fully available without restriction and that the data and code supporting the findings are publicly available in a GitHub repository. However, the repository description indicates that the original dataset cannot be publicly released and that only preprocessed train and test data are provided. The authors should specify precisely what is available, what is restricted, and whether the shared materials are sufficient for independent reproduction of the reported analyses.

Response: We thank the reviewer for pointing out the ambiguity in the data availability statement.

Because the study dataset consists of multicenter clinical visual field data containing sensitive patient information, the complete raw dataset cannot be publicly released due to institutional and ethical restrictions. We have therefore revised the data availability statement to clearly distinguish between publicly available and restricted materials.

The study materials are publicly available at the following GitHub repository: https://github.com/kimhb1029/Visual_Field_Progression

Specifically, the GitHub repository includes:
(1) the complete analysis and preprocessing code used in this study,
(2) the fully processed train and test datasets used for machine learning analyses, and
(3) a small sample of de-identified raw visual field data provided for demonstration of the preprocessing pipeline.

Using the provided sample raw data together with the shared preprocessing code, users can reproduce the generation of eye-episodes and verify the preprocessing workflow described in the manuscript. In addition, the released processed datasets and full analysis scripts are sufficient to reproduce the reported machine learning experiments and performance results.

3. The reporting of follow-up timing appears internally inconsistent and should be clarified. Table 1 reports a mean follow-up duration of 1.53 years and exactly 5.0 visual field tests per eye-episode, whereas Table 2 reports the interval between visual field examinations as approximately 1.93 years in both training and test sets. As currently labeled, these quantities do not appear to be compatible and may reflect a unit error, mislabeled variable, or reporting inconsistency.

Response: Thank you for pointing out this inconsistency. We agree that the originally reported follow-up duration in Table 1 was incorrect. The value has now been corrected from 1.53 ± 1.24 years to 4.434 ± 2.596 years.

The revised value has been updated in Table 1 of the manuscript.

4. There appears to be an incorrect table citation in the Methods section. In the description of the Wiggs-based criteria, the manuscript refers to Table 3, whereas the Wiggs-related counts appear in Table 5. This should be corrected for clarity.

Response: Thank you for pointing this out. We confirmed that the counts related to the Wiggs-based criteria are correctly presented in Table 5. In the revised manuscript, we have corrected the table citation to Table 5.

5. Please clarify the decision threshold used for the reported sensitivity, specificity, precision, and F1 score values. The manuscript refers to a fixed decision threshold, but the threshold itself is not clearly specified.

Response: Thank you for your comment. In this study, sensitivity, specificity, precision, and F1 score were not calculated using separately optimized thresholds. Instead, they were derived from the default binary class outputs of each classifier. For logistic regression and XGBoost, class labels were assigned using the default probability threshold of 0.5. For SVM and random forest, the default prediction rules implemented in scikit-learn were used.

6. Please provide complete implementation details for all models, especially XGBoost, including package versions and key hyperparameters.

Response: We thank the reviewer for requesting additional implementation details. We revised the Methods section to provide more complete information on the implementation of all machine learning models. All models were implemented in Python3.12 using scikit-learn(1.7.2) and XGBoost(3.2.0). The following classifiers were used: support vector machine, random forest, logistic regression, and XGBoost. A fixed random seed was applied to ensure reproducibility of the train–test split and model training.

No additional hyperparameter optimization was performed, because the primary aim of this study was to compare the effect of labeling strategies under the same modeling framework rather than to maximize the performance of individual classifiers. Therefore, all models were trained using the default hyperparameters of the corresponding packages, except for the fixed random seed. We have now explicitly stated this in the revised manuscript and added the package versions and key model settings, including those for XGBoost.

7. The preprocessing workflow would be easier to evaluate if the manuscript quantified how many eyes, segments, or episodes were excluded at each filtering step.

Response: We thank the reviewer for this helpful suggestion. To improve transparency of the preprocessing workflow, we have quantified the number of records retained after each filtering step and added this information to the revised manuscript (Supplementary Figure 1).

The initial dataset consisted of 364,153 visual field (VF) examinations from 74,722 patients and 144,123 eyes collected between June 2004 and January 2021. After deduplication and restricting the cohort to patients aged ≥18 years, 347,514 VF examinations remained. Application of reliability criteria (false-positive rate <15% and fixation loss rate <33%) yielded 271,070 VF examinations from 113,739 eyes. After excluding the first VF examination from each eye to minimize learning effects, 157,331 VF examinations remained. Restricting the dataset to eyes with at least five VF examinations resulted in 96,867 VF examinations. Following the segmentation and preprocessing procedures described in the Methods, the final analytical dataset comprised 47,570 VF examinations corresponding to 9,514 eye-episodes from 7,196 eyes and 4,496 patients.

Supplementary Figure 1. Flowchart of the visual field dataset preprocessing pipeline. The number of visual field examinations, eyes, and patients retained after each filtering step are shown. VF, visual field.

8. Table 2’s independent t-tests comparing the random train and test splits are not especially informative. It would be more useful to report class balance, center balance, and disease severity balance across the two splits.

Response: We thank the reviewer for this helpful suggestion. We agree that independent t-tests comparing randomly generated train and test splits provide limited additional information. In response, we revised Table 2 to include the class balance between progression and non-progression cases across the training and test datasets. This revision provides a more informative assessment of the comparability between the two splits in the context of machine learning model development.

Table 2. Demographic and visual field–derived features used as machine learning inputs in the training and test sets. Age and the interval between visual field examinations are reported as the mean and standard deviation for each eye-episode, whereas all other variables are presented as the mean and standard deviation of the change values, calculated as the difference between the late-phase and early-phase averages within each eye-episode.

|  |  | Training data (n=7611) | Test data  (n=1903) |
| --- | --- | --- | --- |
| Dataset characteristics | Age (year), mean $\pm$ SD | 56.755 ± 14.484 | 56.656 ± 13.932 |
|  | The intervals between visual field examinations (year), mean $\pm$ SD | 1.933 ± 0.546 | 1.924 ± 0.536 |
|  | Consensus label: stable / progressing, n (%) | 5333 (70.1%) / 2278 (29.9%) | 1334 (70.1%) / 569 (29.9%) |
|  | Wiggs’ label: stable / progressing, n (%) | 4814 (63.3%) / 2797 (36.7%) | 1203 (63.2%) / 700 (36.8%) |
| Features used for Machine Learning | AGIS, mean $\pm$ SD | 0.179 ± 1.744 | 0.176 ± 1.718 |
|  | CIGTS, mean $\pm$ SD | 0.012 ± 2.561 | −0.006 ± 2.533 |
|  | MD, mean $\pm$ SD | −0.154 ± 1.87 | −0.149 ± 1.81 |
|  | MD slope, mean $\pm$ SD | −0.14 ± 0.429 | −0.129 ± 0.394 |
|  | VFI, mean $\pm$ SD | −1.026 ± 5.408 | −1.08 ± 5.239 |
|  | VFI slope, mean $\pm$ SD | −0.469 ± 1.466 | −0.427 ± 1.373 |
|  | Mean 52 TDV, mean $\pm$ SD | −0.149 ± 1.905 | −0.143 ± 1.861 |
|  | Mean 52 PDV, mean $\pm$ SD | −0.21 ± 1.16 | −0.231 ± 1.133 |

9. The limitation that the Consensus label reflects only functional progression and is not linked to structural OCT evidence is important and should be emphasized clearly, potentially earlier in the manuscript.

Response: We thank the reviewer for this important point. We agree that the absence of structural OCT validation represents a meaningful limitation that warrants more prominent acknowledgment. In the revised manuscript, we have expanded the Limitations paragraph to more explicitly address this issue. Specifically, we have added text clarifying that: (1) the Consensus label is derived exclusively from functional visual field data and does not incorporate structural information from OCT; (2) structural changes detected by OCT, particularly RNFL and macular ganglion cell layer thinning, are known to precede functional visual field loss in glaucoma, meaning that some eyes with genuine structural progression may not have been captured by our functional labeling scheme; and (3) future studies integrating structural OCT data into the labeling framework could further improve the validity and completeness of the ground truth definition. These additions have been incorporated into the Limitations section with supporting references as shown below.

“Several limitations of this study warrant consideration. First, the Consensus label was derived exclusively from functional visual field data and was not linked to structural OCT evidence, such as retinal nerve fiber layer or macular ganglion cell layer thickness measurements. This is a critical limitation, as structural changes detected by OCT are known to frequently precede functional visual field loss in glaucoma, suggesting that some eyes with genuine early structural progression may have been labeled as non-progressing under the current framework [1, 2]. The absence of structural validation means that the Consensus label, despite its functional sensitivity, cannot be considered a fully comprehensive ground truth. Future studies incorporating longitudinal OCT data alongside VF measurements into the labeling strategy would be valuable to assess whether a combined structure–function label further improves model performance and clinical validity [2].”

[1] Weinreb RN, Aung T, Medeiros FA. The pathophysiology and treatment of glaucoma: a review. JAMA. 2014 May 14;311(18):1901-11. doi: 10.1001/jama.2014.3192. PMID: 24825645; PMCID: PMC4523637.

[2] Tatham AJ, Medeiros FA. Detecting Structural Progression in Glaucoma with Optical Coherence Tomography. Ophthalmology. 2017 Dec;124(12S):S57-S65. doi: 10.1016/j.ophtha.2017.07.015. PMID: 29157363; PMCID: PMC6882427.

10. The description of the regional partition used for the Wiggs label is not fully clear. The Methods state that pattern deviation plots were divided into six peripheral regions and two paracentral regions, but the parenthetical description appears to name only superior and inferior nasal step and superior and inferior Bjerrum areas. Please describe the regional partition unambiguously.

Response: We thank the reviewer for identifying this ambiguity. The original parenthetical description was incomplete. The six peripheral regions should include not only the superior and inferior nasal steps and superior and inferior Bjerrum areas, but also the **superior and inferior temporal wedge regions,** yielding a total of six peripheral regions. We have corrected the relevant sentence in the Methods section accordingly, as shown below.

"In addition, pattern deviation plots were divided into six peripheral regions (superior and inferior nasal steps, superior and inferior Bjerrum areas, and superior and inferior temporal wedge regions) and two paracentral regions."

11. The Discussion states that the models were compared on the same dataset and architecture with “only the labeling strategy varied.” This wording appears somewhat too strong, because the positive class prevalence also differs between the Consensus and Wiggs labels. The authors should revise this sentence to reflect that the comparison changes not only label definition but also class balance and likely the difficulty of the classification task.

Response: We thank the reviewer for this thoughtful comment. The primary objective of this study was to compare how different progression definitions influence machine learning performance while maintaining the same input features and machine learning framework. As the reviewer correctly noted, differences in positive class prevalence exist between the two labeling strategies, and such differences may influence the difficulty of the classification task.

However, we believe that these differences are an inherent consequence of the labeling strategies themselves rather than an independent factor that should be controlled for. In theory, it would be possible to artificially match the class distributions by excluding a subset of eye-episodes. Nevertheless, such an approach would alter the characteristics of the progression populations defined by each labeling strategy and could distort the practical behavior of the original labeling frameworks. Because our goal was to evaluate the performance implications of each labeling strategy as originally defined, we considered it more appropriate to preserve the natural class distributions associated with each label.

**Reviewer #2**

Major concern 1: Bibliography needs to be extended. Current references are relatively old. Therefore, it is recommended : 1-) update some already references with more recently published papers in the same context, and 2-) you need to add more references related to the title of this manuscript. After visiting google scholar, the following two links could be useful:
<https://scholar.google.com/scholar?hl=en&as_sdt=0%2C5&as_ylo=2025&q=labeling+matters+in+ophthalmology+glaucoma&btnG=>
<https://scholar.google.com/scholar?as_ylo=2025&q=labeling+matters+in+ophthalmology&hl=en&as_sdt=0,5>

After addressing this concern, it is recommended that you can extend the introduction and discussion sections accordingly to the new reference list.
It is good to tell the reader that title of this paper is NOT restricted to "glaucoma". Other applications also have the same argument.
In summary, the current 17 references, in my point of view, need to be remarkably extended along with their associated text specifically in the introduction section.

Response: We thank the reviewer for this constructive suggestion. We agree that the reference list required updating and broadening. In the revised manuscript, we have added two recently published papers (2025) that are directly relevant to the scope of this work. First, Mungle et al. (JAMIA Open, 2025) demonstrated that ensemble machine learning models, including LR, RF, and SVC, applied to electronic health records for glaucoma identification face significant class imbalance challenges, and that both data resampling strategies and model selection critically influence performance, consistent with our findings on the impact of labeling strategy. Second, Reddy and Battula (IEEE STCR, 2025) conducted a comprehensive survey of AI applications in glaucoma diagnosis and reported that while deep learning accounts for the largest proportion of published methods (31.5%), conventional machine learning, particularly SVM, remains widely used (25.9%), supporting the continued relevance of the traditional ML classifiers employed in our study. We have incorporated these references into the Introduction with corresponding explanatory text, as detailed below

“The application of machine learning to glaucoma-related tasks has grown substantially in recent years. A comprehensive survey of AI methods in glaucoma diagnosis reported that deep learning accounts for the largest proportion of published approaches (31.5%), yet conventional machine learning methods, particularly support vector machines, remain widely adopted, representing 25.9% of reported methods, underscoring the continued clinical and methodological relevance of traditional classifiers.^15^ Beyond model architecture, the quality and definition of training labels have emerged as a critical determinant of model performance across ophthalmic AI applications. For instance, a recent multicenter study applying ensemble machine learning models, including logistic regression, random forest, and support vector classifier, to electronic health records for glaucoma identification demonstrated that severe class imbalance and heterogeneous data definitions substantially impaired model performance, and that resampling strategies designed to address label distribution directly improved classification outcomes.^16^ These findings, alongside evidence from broader medical AI literature,^12–14^ indicate that the challenge of ground truth definition is not unique to glaucoma but represents a fundamental issue across AI-based clinical applications. The present study addresses this challenge directly by systematically comparing the effect of labeling strategy on machine learning performance in visual field progression detection.”

Major concern 2: In my point of view, it would be complementary correct to mention the machines (types of images or tests) used in diagnosing eyes in Tables 1 and 2, in each center whom participated in this study.

Response: We thank the reviewer for this suggestion. We acknowledge that providing device-level information for each participating center would be ideal for transparency and reproducibility. However, we would like to clarify the following points regarding the scope and design of this study.

First, all five participating centers performed a standardized set of comprehensive ophthalmological examinations for all patients, including best-corrected visual acuity, slit-lamp biomicroscopy, intraocular pressure measurement using Goldmann applanation tonometry, gonioscopy, fundus examination, and keratometry using an auto-kerato-refractometer. This examination protocol was common across all centers, ensuring a consistent clinical baseline for patient inclusion.

Second, while specific device models varied across centers, the clinical examination protocol was standardized, and the primary data source, Humphrey visual field testing, was uniform across all sites. Detailed center-by-center device inventories were not systematically collected as part of the study protocol, as this study was not designed to compare device-specific performance.

In response to the reviewer's comment, we have added a sentence to the Methods section explicitly describing the standardized examination protocol performed at all centers.

“At all five participating centers, patients underwent comprehensive ophthalmological examinations including best-corrected visual acuity, slit-lamp biomicroscopy, intraocular pressure measurement using Goldmann applanation tonometry, gonioscopy, fundus examination, and keratometry using an auto-kerato-refractometer.”

**Reviewer #3**

**Major comments**

1. The study addresses an important and underappreciated issue in glaucoma progression modeling: the extent to which the definition of the outcome label determines apparent machine-learning performance. However, the manuscript does not always keep this distinction clear. At several points, the study is presented as if it developed a clinically reliable detector of glaucomatous visual field progression, whereas the actual analysis mainly shows that one algorithm-derived label was more readily reproduced from selected visual-field summary features than another. The authors should more clearly frame the study as an investigation of label behavior and model-label compatibility, rather than as validation of a clinically useful progression-detection system.

Response: We sincerely thank the reviewer for this insightful and constructive comment, which identifies a genuine tension in how the study is framed. We fully agree that the core contribution of this work is an investigation of label behavior and model-label compatibility, and that certain passages in the original manuscript overstated the clinical readiness of the models presented. We have revised the manuscript accordingly to ensure this distinction is consistently maintained throughout.

The abstract conclusion originally stated that "These findings underscore that careful definition of ground truth is critical for developing reliable artificial intelligence systems for glaucoma progression assessment." which implied clinical validation of the models. We have revised this to make clear that the study evaluates how labeling strategy shapes model behavior, and that the performance differences observed reflect the differential learnability of each label from VF summary features—not the clinical superiority of any detection system per se. The revised conclusion now reads:

“The observed performance differences primarily reflect model-label compatibility rather than the clinical validity of either detection system, underscoring that careful definition of ground truth is critical for interpreting ML-based glaucoma progression research.”

We have added a clarifying statement in the Introduction to explicitly frame the study objective as an analysis of label behavior and model-label compatibility:

"Importantly, the present study is not designed to validate a clinically deployable progression-detection system. Rather, it investigates how the choice of outcome label determines the apparent performance of ML classifiers trained on the same dataset, and what this reveals about the learnability and internal structure of each labeling scheme."

The conclusion has been revised to explicitly situate the study as a label-behavior investigation, and the phrase "clinically valid ground truth candidate" has been replaced with language that acknowledges the need for future external validation before clinical deployment can be considered:

"In summary, this study demonstrates that the labeling strategy used to define glaucomatous progression is a critical determinant of apparent ML performance, and that differences in model performance across labeling schemes primarily reflect model-label compatibility given the input feature space. Whether the Consensus label constitutes a clinically valid ground truth remains an open question that requires prospective validation incorporating structural endpoints."

We believe these revisions collectively reframe the manuscript as an investigation of label behavior and model-label compatibility, as the reviewer appropriately recommended, while preserving the scientific contribution of the work. We hope the revised framing adequately addresses this concern.

2. I am concerned about the use of the term “Consensus label.” The proposed label is defined as progression according to at least one of five algorithms. This is an inclusive OR rule, not a consensus rule in the usual sense of agreement among methods. This point is not merely semantic, because the term “consensus” may imply greater clinical validity or reliability than the definition supports. The authors should either rename this label, for example as an “inclusive composite algorithmic label,” or provide a stronger justification for why a one-positive-rule should be regarded as a consensus definition.

Response: Despite the clinical importance of detecting glaucomatous visual field (VF) progression, substantial disagreement exists among commonly used progression algorithms. Each algorithm, including the Advanced Glaucoma Intervention Study (AGIS) score, the Collaborative Initial Glaucoma Treatment Study (CIGTS) score, mean deviation (MD) slope, visual field index (VFI) slope, and pointwise linear regression (PLR), captures distinct aspects of VF deterioration through different analytical frameworks, and no single method has demonstrated consistently superior performance across all clinical scenarios [1, 2].

Indeed, significant discordance has been reported among these traditional algorithms, with only a small proportion of eyes classified identically across all methods [2-5]. Furthermore, relying solely on any single algorithm carries an inherent risk of false-negative classification, as each method may miss progression signals that others detect [6]. This variability poses a fundamental challenge when constructing labeled datasets for supervised machine learning, as the choice of labeling strategy directly determines what the model learns to detect.

To address this challenge, we proposed a Consensus label defined as positive when at least one of the five algorithms detected progression. This "any-positive" strategy was adopted based on both clinical and methodological rationale. Glaucoma is the leading cause of irreversible blindness worldwide, and because visual loss once incurred cannot be restored, early detection and timely intervention are paramount [7]. Given this irreversibility, the clinical consequence of a missed progression event (false negative) far outweighs the cost of a false-positive alert. Since each algorithm may detect progression signals that others miss, particularly given that different methods are sensitive to different patterns of VF loss, requiring agreement among multiple algorithms before assigning a positive label risks systematically discarding genuine progression signals present in the training data. The any-positive Consensus label was therefore designed to maximize sensitivity in label assignment, ensuring that eyes with any algorithm-detectable signal of deterioration are retained as positive cases, consistent with the clinical principle that any indicator of progression in an irreversible disease warrants heightened vigilance.

[1] Dixit A, Yohannan J, Boland MV. Assessing Glaucoma Progression Using Machine Learning Trained on Longitudinal Visual Field and Clinical Data. Ophthalmology. 2021;128(7):1016-1026. doi:10.1016/j.ophtha.2020.12.020

[2] Rabiolo A, Morales E, Mohamed L, et al. Comparison of Methods to Detect and Measure Glaucomatous Visual Field Progression. Transl Vis Sci Technol. 2019;8(5):2. doi:10.1167/tvst.8.5.2

[3] Antón A, Pazos M, Martín B, Navero JM, Ayala ME, Castany M, Martínez P, Bardavío J. Glaucoma progression detection: agreement, sensitivity, and specificity of expert visual field evaluation, event analysis, and trend analysis. Eur J Ophthalmol. 2013 Mar-Apr;23(2):187-95. doi: 10.5301/ejo.5000193. PMID: 23065852.

[4] Rao HL, Kumbar T, Kumar AU, Babu JG, Senthil S, Garudadri CS. Agreement between event-based and trend-based glaucoma progression analyses. Eye (Lond). 2013 Jul;27(7):803-8. doi: 10.1038/eye.2013.77. Epub 2013 Apr 19. PMID: 23598668; PMCID: PMC3709397.

[5] Wu Z, Medeiros FA. Comparison of Visual Field Point-Wise Event-Based and Global Trend-Based Analysis for Detecting Glaucomatous Progression. Transl Vis Sci Technol. 2018 Aug 27;7(4):20. doi: 10.1167/tvst.7.4.20. PMID: 30159212; PMCID: PMC6111742.

[6] Tsamis E, La Bruna S, Rai A, Leshno A, Grossman J, Cioffi G, Liebmann JM, De Moraes CG, Hood DC. Progression of Early Glaucomatous Damage: Performance of Summary Statistics From Optical Coherence Tomography and Perimetry. Transl Vis Sci Technol. 2023 Mar 1;12(3):19. doi: 10.1167/tvst.12.3.19. PMID: 36939711; PMCID: PMC10043504.

[7] Tham YC, Li X, Wong TY, Quigley HA, Aung T, Cheng CY. Global prevalence of glaucoma and projections of glaucoma burden through 2040: a systematic review and meta-analysis. Ophthalmology. 2014;121(11):2081–2090. PMID: 24974815

We have added following sentences in the Introduction section.

“Furthermore, relying solely on any single algorithm carries an inherent risk of false-negative classification, as each method may miss progression signals that others detect.^10^ This variability poses a fundamental challenge when constructing labeled datasets for supervised machine learning, as the choice of labeling strategy directly determines what the model learns to detect.”

“To address this challenge, we proposed a “Consensus label” defined as positive when at least one of the five algorithms detected progression. This "any-positive" strategy was adopted based on both clinical and methodological rationale. Glaucoma is the leading cause of irreversible blindness worldwide, and because visual loss once incurred cannot be restored, early detection and timely intervention are paramount.^11^ Given this irreversibility, the clinical consequence of a missed progression event (false negative) far outweighs the cost of a false-positive alert. Since each algorithm may detect progression signals that others miss, particularly given that different methods are sensitive to different patterns of VF loss, requiring agreement among multiple algorithms before assigning a positive label risks systematically discarding genuine progression signals present in the training data. The any-positive Consensus label was therefore designed to maximize sensitivity in label assignment, ensuring that eyes with any algorithm-detectable signal of deterioration are retained as positive cases, consistent with the clinical principle that any indicator of progression in an irreversible disease warrants heightened vigilance.”

3. The study lacks an independent reference standard for true glaucomatous progression. Both the Consensus label and the Wiggs’ label are derived from visual-field rules, and neither is validated here against masked expert adjudication, OCT or optic disc progression, subsequent visual-field worsening, treatment escalation, or another clinically independent endpoint. Therefore, the results cannot establish that either label is closer to true disease progression. Claims such as “clinically reliable detection,” “minimal false positives,” “clinically valid ground truth,” and “early warning systems” should be substantially toned down unless additional validation is provided.

Response: We thank the reviewer for this important and valid critique. We fully agree that neither the Consensus label nor the Wiggs' label has been validated against an independent clinical reference standard, and that the absence of such validation, whether from masked expert adjudication, structural OCT progression, optic disc changes, subsequent visual field worsening, or treatment escalation, means that neither label can be claimed to represent true glaucomatous progression. We have revised the manuscript accordingly to remove or substantially tone down all claims that imply clinical validity beyond what the study design supports.

Specifically, the following expressions have been revised:

1. "clinically reliable detection performance" (Results) → "high classification performance relative to the training label"

2. "minimal false positives" (Abstract Conclusion) → "high specificity with respect to the Consensus label definition"

3. "clinically valid ground truth candidate" "early warning systems" (Conclusion) → In summary, this study demonstrates that the labeling strategy used to define glaucomatous progression is a critical determinant of apparent ML performance, and that differences in model performance across labeling schemes primarily reflect model-label compatibility given the input feature space. Whether the Consensus label constitutes a clinically valid ground truth remains an open question that requires prospective validation incorporating structural endpoints.

4. There is a potentially serious circularity problem between the labels and the input features. The Consensus label is constructed from AGIS, CIGTS, MD slope, VFI slope, and PLR, while the model inputs include closely related quantities such as AGIS score, CIGTS score, MD, MD slope, VFI, VFI slope, and TDV/PDV summaries. Under this design, high AUC and specificity may largely reflect the model’s ability to reconstruct an algorithmic label from overlapping features. The current ablation analysis is useful, but it does not fully address this concern, because correlated features remain in the model and the target label still contains information derived from the same family of measurements. A leakage-reduced sensitivity analysis excluding all features directly or indirectly used to define each target label would be important.

Response: As the reviewer appropriately pointed out, there is both a conceptual and statistical relationship between the criteria used to construct the Consensus label and some of the model input variables. Specifically, the Consensus label was defined based on rule-based progression indicators, including AGIS_test, CIGTS_test, MD_slope_test, VFI_slope_test, and PLR_test, whereas the model input variables included AGIS, CIGTS, MD, VFI, MD slope value, VFI slope value, and TDV/PDV-derived summary metrics. Therefore, it is possible that a portion of the model performance reflects information that is also related to the label definition.

However, we would like to emphasize that the label-defining variables and predictor variables used in this study were not direct duplicates of the same variables. The Consensus label was defined using binary rule-based indicators that determine whether specific progression criteria were met, whereas the model inputs consisted of continuous numerical values or summary statistics. For example, AGIS_test is a binary indicator that evaluates whether a predefined level of change from baseline has occurred consistently over time, whereas AGIS refers to the raw score itself at each visit. Similarly, MD_slope_test and VFI_slope_test are binary outcome variables defined according to progression criteria that incorporate statistical significance, whereas the predictor variables MD slope and VFI slope values represent the continuous magnitude of change itself. Therefore, we considered the structure of our study to be closer to the use of progression-related clinical measures in different forms, rather than a case of direct label leakage resulting from the reuse of identical variables.

In addition, the objective of this study was not to construct a completely independent prediction model after excluding all progression-related features. Rather, our aim was to investigate how different labeling strategies influence machine learning performance when clinically relevant progression descriptors are used as model inputs. Therefore, the inclusion of progression-related clinical variables as features was an intentional aspect of the study design.

Nevertheless, we agree that the possibility of circularity raised by the reviewer represents an important methodological consideration. To further evaluate this issue, we performed an additional ablation analysis (included in the Supplementary Materials) in which feature groups associated with individual progression criteria were removed one at a time, and model performance was re-evaluated. The results showed that the overall performance patterns were largely preserved despite the removal of specific feature groups, suggesting that the observed performance cannot be explained solely by a single progression criterion or simple one-to-one feature duplication.

5. The comparison between the Consensus and Wiggs’ labels may be confounded by the choice of input representation. The Consensus label is closely aligned with global and trend-based summary features, whereas the Wiggs’ label is based more on regional and spatially organized visual-field defects. If the model receives only hand-crafted summary features rather than the raw visual-field map or a richer regional representation, the Wiggs’ label may be disadvantaged by design. Thus, lower performance with the Wiggs’ label should not be interpreted as evidence that the label is inferior. The authors should discuss this issue explicitly and avoid equating predictability with clinical validity.

Response: We thank the reviewer for this important comment. Not only the Consensus label but also the Wiggs’ label demonstrated correlations with progression-related variables, and several spatial variability-related features showed even stronger correlations with the Wiggs’ label. This suggests that the association between progression-related clinical descriptors and the labels is not unique to a particular labeling strategy but rather reflects a broader structural characteristic shared across different definitions of glaucoma progression. Therefore, we believe that the observed model performance is better explained by the integration of multiple progression-related patterns than by direct feature duplication alone. The correlations between the input features and the progression labels are shown in the following:

Supplementary Table 6. Correlation between input features and progression labels (Consensus label and Wiggs’ label)

|  | AGIS | CIGTS | MD | MD slope | VFI | VFI slope | TDV mean | TDV std | PDV mean | PDV std |
| --- | --- | --- | --- | --- | --- | --- | --- | --- | --- | --- |
| Consensus label | 0.26 | 0.25 | −0.32 | −0.50 | −0.29 | −0.49 | −0.31 | 0.22 | −0.23 | 0.22 |
| Wiggs’ label | 0.26 | 0.18 | −0.29 | −0.34 | −0.32 | −0.37 | −0.29 | 0.49 | −0.31 | 0.49 |

We have added the following sentences in the Discussion section.

“A potential concern is that the observed performance difference between the two labeling strategies may partly reflect an asymmetry in input representation rather than a true difference in label quality. The Consensus label is algorithmically aligned with global trend-based features such as MD slope and VFI slope, which were among the strongest predictors in the current feature set (r = −0.50 and −0.49, respectively), potentially conferring an inherent advantage to Consensus label–based models. However, examination of feature-label correlations revealed that the Wiggs' label showed comparatively stronger associations with spatial variability features, particularly TDV standard deviation and PDV standard deviation (r = 0.49 for both), which were less prominent predictors of the Consensus label (Supplementary Table 6). This pattern suggests that each label captures a distinct and internally coherent dimension of VF deterioration, and that the lower predictive performance observed under the Wiggs' label reflects the relative underrepresentation of spatially granular features in the current input set rather than the clinical inferiority of the label itself. Future studies incorporating raw VF maps or richer regional representations may better capture the spatially organized defect patterns underlying the Wiggs' label and yield more balanced model performance across labeling strategies.”

6. The validation strategy needs to be strengthened or, at minimum, described more transparently. The Methods indicate an 80:20 train–test split with a fixed random seed, but it is unclear whether the split was performed at the patient, eye, or eye-episode level. Because multiple eye-episodes can come from the same eye or patient, random episode-level splitting could inflate performance. The authors should clarify the splitting unit and provide patient-level or eye-level validation. Repeated cross-validation or bootstrapped confidence intervals would also make the performance estimates more credible. Since the study emphasizes a multicenter dataset, a leave-one-center-out analysis would be a particularly informative assessment of generalizability.

Response: We thank the reviewer for this important comment. As described in the original manuscript, 80% of all eye-episodes were used as the training dataset and the remaining 20% were used as the test dataset (“Of all eye-episodes, 80% were used as the training dataset and the remaining 20% were used as the test dataset.”). Because the present study was conducted at the eye-episode level, multiple eye-episodes originating from the same patient or eye could potentially be included in both the training and test datasets. This may introduce correlated information between the two datasets and could lead to optimistic estimates of model performance.

However, the primary objective of this study was not to develop a strictly patient-independent progression-classification model, but rather to compare how different labeling strategies influence the performance of machine learning models in learning longitudinal progression patterns. In addition, glaucomatous progression may manifest as heterogeneous longitudinal progression patterns over time, even within the same patient. Therefore, we considered eye-episode–level analysis to be appropriate for capturing a broader range of progression dynamics.

Nevertheless, to evaluate the potential impact raised by the reviewer, we performed an additional sensitivity analysis using a patient-level split, and the results have been provided in Supplementary Table 7-9. After ensuring that all eye-episodes originating from the same patient were assigned exclusively to either the training or test dataset, the relative performance trends across labeling strategies and the main study conclusions remained consistent. These findings suggest that the primary observations of this study are not solely dependent on eye-episode–level data partitioning and that the comparative results of the proposed labeling strategies are relatively robust.

Supplementary Table 7. To prevent information leakage, the dataset was divided into training and test sets at the patient level. All eye-episodes from a given patient, including both eyes and repeated episodes from the same eye, were assigned exclusively to either the training or test set.

|  |  | Training data (n=7641) | Test data  (n=1873) |
| --- | --- | --- | --- |
| Dataset characteristics | Age (year), mean $\pm$ SD | 56.738 ± 14.331 | 56.723 ± 14.555 |
|  | The intervals between visual field examinations (year), mean $\pm$ SD | 1.93 ± 0.54 | 1.938 ± 0.558 |
|  | Consensus label: stable / progressing, n (%) | 5365 (70.2%) / 2276 (29.8%) | 1302 (69.5%) / 571 (30.5%) |
|  | Wiggs’ label: stable / progressing, n (%) | 4846 (63.4%) / 2795 (36.6%) | 1171 (62.5%) / 702 (37.5%) |
| Features used for Machine Learning | AGIS, mean ± SD | 0.181 ± 1.728 | 0.17 ± 1.782 |
|  | CIGTS, mean ± SD | 0.012 ± 2.552 | -0.008 ± 2.57 |
|  | MD, mean ± SD | -0.148 ± 1.834 | -0.173 ± 1.953 |
|  | MD slope, mean ± SD | -0.137 ± 0.422 | -0.143 ± 0.426 |
|  | VFI, mean ± SD | -1.054 ± 5.293 | -0.966 ± 5.697 |
|  | VFI slope, mean ± SD | -0.462 ± 1.445 | -0.455 ± 1.461 |
|  | Mean 52 TDV, mean ± SD | -0.143 ± 1.877 | -0.167 ± 1.972 |
|  | Mean 52 PDV, mean ± SD | -0.217 ± 1.147 | -0.203 ± 1.186 |

Supplementary Table 8. Classification performance of machine learning models for detecting visual field progression using the Consensus label with patient-level train–test splitting

|  | Accuracy  (95% CI) | Sensitivity  (95% CI) | Specificity  (95% CI) | Precision  (95% CI) | F1 score  (95% CI) |
| --- | --- | --- | --- | --- | --- |
| SVM | 0.939  (0.928-0.950) | 0.809  (0.777-0.841) | 0.996  (0.992-0.999) | 0.989  (0.979-0.998) | 0.890  (0.869-0.909) |
| RF | 0.941  (0.929-0.952) | 0.823  (0.792-0.855) | 0.993  (0.988-0.997) | 0.981  (0.968-0.993) | 0.895  (0.875-0.913) |
| LR | 0.935  (0.923-0.946) | 0.792  (0.760-0.824) | 0.998  (0.996-1.000) | 0.996  (0.989-1.000) | 0.882  (0.860-0.902) |
| XGBoost | 0.937  (0.926-0.947) | 0.813  (0.780-0.845) | 0.992 (0.987-0.996) | 0.977  (0.963-0.989) | 0.887  (0.863-0.906) |

Supplementary Table 9. Classification performance of machine learning models for detecting visual field progression using the Wiggs’ label with patient-level train–test splitting

|  | Accuracy  (95% CI) | Sensitivity  (95% CI) | Specificity  (95% CI) | Precision  (95% CI) | F1 score  (95% CI) |
| --- | --- | --- | --- | --- | --- |
| SVM | 0.809  (0.791-0.826) | 0.651  (0.618-0.688) | 0.904  (0.886-0.921) | 0.802  (0.770-0.835) | 0.719  (0.691-0.747) |
| RF | 0.805  (0.788-0.821) | 0.687  (0.653-0.721) | 0.875  (0.855-0.894) | 0.768  (0.734-0.800) | 0.725  (0.697-0.752) |
| LR | 0.800  (0.784-0.818) | 0.613  (0.575-0.649) | 0.913  (0.897-0.929) | 0.808  (0.772-0.840) | 0.697  (0.669-0.724) |
| XGBoost | 0.799  (0.780-0.816) | 0.682  (0.648-0.715) | 0.869  (0.850-0.889) | 0.758  (0.726-0.793) | 0.718  (0.692-0.744) |

We also note that, as suggested by the reviewer, uncertainty in model performance was further quantified by estimating 95% confidence intervals using 1000 bootstrap resamples, which have been added to the revised tables (Table 4 and 6).

Table 4. Classification performance of machine learning models for detecting visual field progression using the Consensus label

|  | Accuracy  (95% CI) | Sensitivity  (95% CI) | Specificity  (95% CI) | Precision  (95% CI) | F1 score  (95% CI) |
| --- | --- | --- | --- | --- | --- |
| SVM | 0.947  (0.936-0.956) | 0.824  (0.790-0.853) | 0.999  (0.998-1.000) | 0.998  (0.993-1.000) | 0.903  (0.882-0.920) |
| RF | 0.955  (0.944-0.964) | 0.852  (0.821-0.879) | 0.999  (0.996-1.000) | 0.996  (0.990-1.000) | 0.919  (0.899-0.934) |
| LR | 0.945  (0.935-0.955) | 0.817  (0.783-0.846) | 1.000  (1.000-1.000) | 1.000  (1.000-1.000) | 0.899  (0.879-0.917) |
| XGBoost | 0.951  (0.941-0.960) | 0.849  (0.817-0.877) | 0.995  (0.991-0.998) | 0.986  (0.974-0.996) | 0.912  (0.891-0.929) |

CI, Confidence Interval; SVM, Support Vector Machine; RF, Random Forest; LR, Logistic Regression

Table 6. Classification performance of machine learning models for detecting visual field progression using the Wiggs’ label.

|  | Accuracy  (95% CI) | Sensitivity  (95% CI) | Specificity  (95% CI) | Precision  (95% CI) | F1 score  (95% CI) |
| --- | --- | --- | --- | --- | --- |
| SVM | 0.826  (0.809-0.843) | 0.660  (0.624-0.694) | 0.922  (0.907-0.936) | 0.831  (0.802-0.859) | 0.736  (0.707-0.761) |
| RF | 0.828  (0.811-0.844) | 0.717  (0.683-0.747) | 0.892  (0.875-0.910) | 0.794  (0.766-0.825) | 0.754  (0.728-0.776) |
| LR | 0.811  (0.794-0.827) | 0.630  (0.594-0.665) | 0.916  (0.901-0.932) | 0.814  (0.782-0.845) | 0.710  (0.683-0.737) |
| XGBoost | 0.799  (0.780-0.816) | 0.681  (0.647-0.716) | 0.867  (0.848-0.888) | 0.749  (0.715-0.782) | 0.714  (0.685-0.739) |

CI, Confidence Interval; SVM, Support Vector Machine; RF, Random Forest; LR, Logistic Regression

In response to the reviewer’s suggestion, we additionally performed a leave-one-center-out validation analysis. The results are presented in Supplementary Table 10 and demonstrate the performance and generalizability of the models when applied to data from centers not included in model training. We have added following sentences in the Results section.

“To assess the generalizability of the models across participating institutions, a leave-one-center-out validation analysis was additionally performed. In each iteration, one of the five centers was held out as an independent test set while models were trained on data from the remaining four centers. Under the Consensus label, models consistently achieved high accuracy (0.934–0.964) and sensitivity (0.786–0.890) with near-perfect specificity (0.987–1.000) across all held-out centers. Under the Wiggs' label, models maintained moderate-to-high accuracy (0.791–0.834) and specificity (0.821–0.926), with sensitivity ranging from 0.607 to 0.757. The relative performance patterns across labeling strategies were consistent with those observed in the primary analysis, supporting the robustness of the labeling effect across heterogeneous clinical settings (Supplementary Table 10).”

Supplementary Table 10. Performance of machine learning models under leave-one-center-out validation. In each iteration, one center was held out for testing while models were trained using data from all remaining centers. Results demonstrate model performance and generalizability across participating institutions.

| Removed Center | Label | Model | **Accuracy** | **Recall** | **Specificity** | **Precision** | **F1** |
| --- | --- | --- | --- | --- | --- | --- | --- |
| Dong-A | Consensus | SVM | 0.949 (0.935-0.961) | 0.835 (0.794-0.872) | 0.996 (0.991-1.000) | 0.989 (0.975-1.000) | 0.906 (0.879-0.928) |
|  |  | RF | 0.955 (0.942-0.966) | 0.857 (0.816-0.893) | 0.995 (0.990-0.999) | 0.986 (0.970-0.997) | 0.917 (0.893-0.938) |
|  |  | LR | 0.944 (0.929-0.956) | 0.807 (0.762-0.846) | 1.000 (1.000-1.000) | 1.000 (1.000-1.000) | 0.893 (0.865-0.917) |
|  |  | XGBoost | 0.950 (0.937-0.962) | 0.860 (0.819-0.896) | 0.987 (0.979-0.994) | 0.965 (0.944-0.984) | 0.910 (0.884-0.931) |
|  | Wiggs’ | SVM | 0.795 (0.771-0.820) | 0.724 (0.683-0.763) | 0.852 (0.822-0.880) | 0.796 (0.760-0.833) | 0.759 (0.727-0.790) |
|  |  | RF | 0.801 (0.776-0.826) | 0.757 (0.716-0.794) | 0.837 (0.807-0.865) | 0.788 (0.753-0.826) | 0.772 (0.741-0.802) |
|  |  | LR | 0.791 (0.766-0.813) | 0.671 (0.626-0.712) | 0.886 (0.860-0.911) | 0.825 (0.788-0.863) | 0.740 (0.706-0.771) |
|  |  | XGBoost | 0.791 (0.767-0.816) | 0.753 (0.716-0.790) | 0.821 (0.791-0.851) | 0.770 (0.735-0.810) | 0.762 (0.733-0.792) |
| Busan Paik | Consensus | SVM | 0.959 (0.949-0.968) | 0.867 (0.837-0.897) | 0.997 (0.994-1.000) | 0.993 (0.984-1.000) | 0.926 (0.908-0.943) |
|  |  | RF | 0.964 (0.955-0.973) | 0.886 (0.856-0.915) | 0.996 (0.993-0.999) | 0.991 (0.981-0.998) | 0.935 (0.919-0.952) |
|  |  | LR | 0.960 (0.951-0.970) | 0.865 (0.834-0.894) | 1.000 (1.000-1.000) | 1.000 (1.000-1.000) | 0.928 (0.910-0.944) |
|  |  | XGBoost | 0.960 (0.950-0.970) | 0.890 (0.862-0.918) | 0.989 (0.983-0.995) | 0.972 (0.957-0.988) | 0.930 (0.911-0.946) |
|  | Wiggs’ | SVM | 0.824 (0.808-0.843) | 0.663 (0.625-0.704) | 0.915 (0.899-0.932) | 0.816 (0.783-0.850) | 0.732 (0.704-0.761) |
|  |  | RF | 0.817 (0.799-0.836) | 0.706 (0.668-0.744) | 0.880 (0.861-0.900) | 0.770 (0.734-0.803) | 0.737 (0.706-0.765) |
|  |  | LR | 0.808 (0.790-0.828) | 0.615 (0.576-0.658) | 0.917 (0.901-0.933) | 0.808 (0.772-0.843) | 0.699 (0.666-0.731) |
|  |  | XGBoost | 0.809 (0.791-0.827) | 0.692 (0.657-0.729) | 0.874 (0.854-0.895) | 0.758 (0.723-0.794) | 0.724 (0.695-0.753) |
| Kosin | Consensus | SVM | 0.948 (0.938-0.957) | 0.832 (0.803-0.861) | 0.999 (0.998-1.000) | 0.998 (0.993-1.000) | 0.908 (0.890-0.924) |
|  |  | RF | 0.958 (0.948-0.967) | 0.876 (0.848-0.901) | 0.994 (0.989-0.998) | 0.984 (0.973-0.994) | 0.927 (0.911-0.942) |
|  |  | LR | 0.949 (0.939-0.958) | 0.836 (0.807-0.863) | 0.999 (0.998-1.000) | 0.998 (0.993-1.000) | 0.910 (0.892-0.926) |
|  |  | XGBoost | 0.958 (0.948-0.966) | 0.876 (0.849-0.901) | 0.994 (0.989-0.998) | 0.984 (0.972-0.994) | 0.927 (0.911-0.942) |
|  | Wiggs’ | SVM | 0.817 (0.799-0.834) | 0.653 (0.617-0.687) | 0.916 (0.900-0.931) | 0.823 (0.790-0.852) | 0.728 (0.700-0.754) |
|  |  | RF | 0.820 (0.801-0.838) | 0.699 (0.665-0.732) | 0.893 (0.875-0.910) | 0.796 (0.763-0.828) | 0.744 (0.716-0.769) |
|  |  | LR | 0.814 (0.795-0.832) | 0.630 (0.592-0.664) | 0.924 (0.910-0.940) | 0.833 (0.801-0.863) | 0.717 (0.686-0.745) |
|  |  | XGBoost | 0.821 (0.802-0.837) | 0.696 (0.662-0.731) | 0.895 (0.877-0.911) | 0.799 (0.765-0.829) | 0.744 (0.716-0.770) |
| Pusan | Consensus | SVM | 0.938 (0.925-0.951) | 0.817 (0.780-0.852) | 1.000 (1.000-1.000) | 1.000 (1.000-1.000) | 0.899 (0.876-0.920) |
|  |  | RF | 0.949 (0.937-0.960) | 0.852 (0.819-0.885) | 0.999 (0.996-1.000) | 0.997 (0.991-1.000) | 0.919 (0.899-0.938) |
|  |  | LR | 0.937 (0.923-0.950) | 0.814 (0.777-0.850) | 1.000 (1.000-1.000) | 1.000 (1.000-1.000) | 0.898 (0.874-0.919) |
|  |  | XGBoost | 0.946 (0.934-0.958) | 0.860 (0.827-0.892) | 0.990 (0.983-0.996) | 0.978 (0.962-0.992) | 0.915 (0.894-0.934) |
|  | Wiggs’ | SVM | 0.812 (0.790-0.834) | 0.636 (0.590-0.680) | 0.906 (0.885-0.927) | 0.782 (0.736-0.825) | 0.701 (0.665-0.736) |
|  |  | Random_Forest | 0.815 (0.792-0.837) | 0.678 (0.633-0.724) | 0.888 (0.865-0.910) | 0.761 (0.715-0.803) | 0.717 (0.678-0.751) |
|  |  | Logistic_Regression | 0.804 (0.782-0.827) | 0.607 (0.561-0.650) | 0.909 (0.888-0.928) | 0.778 (0.732-0.821) | 0.682 (0.643-0.716) |
|  |  | XGBoost | 0.819 (0.798-0.840) | 0.692 (0.648-0.734) | 0.886 (0.863-0.909) | 0.763 (0.718-0.806) | 0.725 (0.688-0.759) |
| Yangsan | Consensus | SVM | 0.939 (0.927-0.951) | 0.802 (0.767-0.836) | 0.999 (0.998-1.000) | 0.998 (0.993-1.000) | 0.889 (0.867-0.910) |
|  |  | Random_Forest | 0.948 (0.936-0.958) | 0.834 (0.798-0.863) | 0.998 (0.994-1.000) | 0.993 (0.985-1.000) | 0.907 (0.885-0.924) |
|  |  | Logistic_Regression | 0.934 (0.922-0.946) | 0.786 (0.748-0.820) | 0.999 (0.997-1.000) | 0.998 (0.993-1.000) | 0.879 (0.855-0.901) |
|  |  | XGBoost | 0.945 (0.934-0.955) | 0.830 (0.795-0.860) | 0.996 (0.992-0.999) | 0.989 (0.978-0.998) | 0.903 (0.881-0.920) |
|  | Wiggs’ | SVM | 0.834 (0.816-0.850) | 0.671 (0.635-0.707) | 0.924 (0.908-0.940) | 0.832 (0.797-0.863) | 0.743 (0.715-0.769) |
|  |  | Random_Forest | 0.831 (0.813-0.849) | 0.736 (0.703-0.768) | 0.884 (0.865-0.903) | 0.779 (0.742-0.813) | 0.757 (0.730-0.783) |
|  |  | Logistic_Regression | 0.824 (0.805-0.841) | 0.641 (0.603-0.676) | 0.926 (0.910-0.941) | 0.829 (0.794-0.860) | 0.723 (0.692-0.751) |
|  |  | XGBoost | 0.819 (0.800-0.838) | 0.717 (0.681-0.752) | 0.875 (0.857-0.896) | 0.762 (0.728-0.798) | 0.739 (0.709-0.767) |

We have added following sentences in the discussion section.

“The robustness of the primary findings was further supported by two additional validation analyses. In a patient-level split analysis, all eye-episodes from the same patient were assigned exclusively to either the training or test set, thereby eliminating potential data leakage arising from episode-level partitioning. Performance trends across labeling strategies remained consistent with those observed in the primary analysis (Supplementary Tables 7–9). Similarly, in a leave-one-center-out analysis in which each of the five participating centers was held out in turn as an independent test set, the relative performance patterns were preserved across all centers under both labeling strategies (Supplementary Table 10). Together, these analyses indicate that the observed labeling effect is not an artifact of the data-splitting strategy or center-specific characteristics, and that the performance differences between the Consensus and Wiggs' labels generalize across heterogeneous multicenter settings.”

7. The decision not to tune hyperparameters is not fully convincing. Although the authors state that this was done to ensure an objective comparison, default or fixed parameters are not necessarily neutral across labels. The two labels may differ in class balance, noise structure, and decision boundaries, and one label may be better matched to default settings than the other. A prespecified tuning strategy applied equally to both labels, or at least a sensitivity analysis showing that the main conclusions are robust to tuning, would strengthen the methodological comparison.

Response: We thank the reviewer for this thoughtful comment. Our original intention was to compare different labeling strategies under an identical modeling framework, and therefore the same default hyperparameters were applied across all experiments. This approach was chosen to minimize additional sources of variation arising from model-specific optimization and to facilitate a direct comparison between labeling strategies.

Nevertheless, to evaluate the potential influence of hyperparameter selection, we performed an additional sensitivity analysis using prespecified hyperparameter tuning applied equally to both labeling strategies. Specifically, grid-search hyperparameter optimization with 5-fold stratified cross-validation was performed using balanced accuracy as the optimization metric. The same hyperparameter search space was applied to all classifiers trained with both the Consensus and Wiggs labels.

The tuned models showed only minor changes in absolute performance compared with the default-parameter models. Importantly, the relative performance pattern remained unchanged, with models trained using the Consensus label consistently outperforming those trained using the Wiggs label across classifiers. These findings suggest that the primary conclusions of the study are robust to reasonable variations in hyperparameter selection and are not primarily driven using default model settings.

The hyperparameter search ranges and the corresponding tuned-model performance results are summarized in the following:

| Model | Hyperparameters |
| --- | --- |
| SVM | C: 0.1, 1, 10, 100; gamma: scale, 0.01, 0.1; kernel: rbf |
| Random Forest | n_estimators: 100, 300; max_depth: None, 10, 20; min_samples_split: 2, 5 |
| Logistic Regression | C: 0.01, 0.1, 1, 10, 100 |
| XGBoost | n_estimators: 100, 300; max_depth: 3, 5, 7; learning_rate: 0.01, 0.1; subsample: 0.8, 1.0 |

|  | | Accuracy | Sensitivity | Specificity | Precision | F1 score |
| --- | --- | --- | --- | --- | --- | --- |
|  |  | (95% CI) | | | | |
| Consensus Tuned | SVM | 0.951  (0.940-0.960) | 0.837  (0.804-0.864) | 0.999  (0.998-1.000) | 0.998  (0.994-1.000) | 0.910  (0.890-0.926) |
|  | RF | 0.955  (0.945-0.963) | 0.851  (0.819-0.878) | 0.999  (0.998-1.000) | 0.998  (0.994-1.000) | 0.918  (0.900-0.934) |
|  | LR | 0.951  (0.941-0.960) | 0.838  (0.807-0.866) | 0.999  (0.996-1.000) | 0.996  (0.989-1.000) | 0.910  (0.891-0.927) |
|  | XGBoost | 0.953  (0.943-0.962) | 0.844  (0.811-0.872) | 0.999  (0.998-1.000) | 0.998  (0.994-1.000) | 0.914  (0.894-0.930) |
| Wiggs Tuned | SVM | 0.826  (0.809-0.843) | 0.660  (0.624-0.694) | 0.922  (0.907-0.936) | 0.831  (0.802-0.859) | 0.736  (0.707-0.761) |
|  | RF | 0.823  (0.806-0.839) | 0.700  (0.667-0.732) | 0.894  (0.877-0.912) | 0.794  (0.763-0.825) | 0.744  (0.717-0.767) |
|  | LR | 0.811  (0.794-0.827) | 0.630  (0.594-0.665) | 0.916  (0.901-0.932) | 0.814  (0.782-0.845) | 0.710  (0.683-0.737) |
|  | XGBoost | 0.815  (0.797-0.831) | 0.683  (0.647-0.717) | 0.892  (0.873-0.910) | 0.786  (0.754-0.818) | 0.731  (0.704-0.756) |

8. The manuscript should distinguish more carefully between classifying progression within a five-test episode and predicting future progression. The models appear to use features derived from the same five visual fields that are used to assign the labels. This is a concurrent classification task, not a prospective prediction task. Therefore, statements about early warning or future clinical decision support are premature unless the authors perform an analysis in which earlier visual fields are used to predict progression occurring later.

Response: We thank the reviewer for this important methodological observation. We agree that the present framework represents a concurrent classification task rather than a prospective prediction task, as both the input features and progression labels were derived from the same five visual field examinations within each eye-episode. We agree that the terms “early progression detection” and “early warning systems” imply a prospective, forward-looking prediction framework, which is not what the present study design supports.

Our study is retrospective in nature: the machine learning models were trained and evaluated on labeled eye-episodes in which all five visual field examinations within each episode were available at the time of labeling. Accordingly, the models classify whether progression occurred within a completed series of observations, rather than predicting future progression from an incomplete or early time series.

In response, we have revised the relevant sentences in the Discussion section to more accurately reflect the retrospective, classification-based nature of our framework. Specifically, “early progression detection” has been replaced with “sensitive identification of progression,” and “early warning systems” has been removed.

Minor comments
1. The abstract should state more explicitly that both labels are algorithm-derived and that no independent clinical reference standard was used. This would prevent readers from overinterpreting the reported AUC and specificity.

Response: We have added following sentences in the Abstract Methods.

“Two algorithm-derived labeling approaches were evaluated without an independent clinical reference standard”

2. The authors should report the exact hyperparameters used for each model, including the SVM kernel and regularization parameter, random forest depth and number of trees, and XGBoost learning rate, depth, number of estimators, and regularization settings.

Response: We thank the reviewer for this comment. No hyperparameter optimization was performed in this study, and all models were trained using the default hyperparameters of the corresponding software packages, except for the fixed random seed. Because the aim of the study was to compare labeling strategies under an identical modeling framework, the same default settings were applied across all experiments

In addition, as described in our response to Major Comment 7, we performed a supplementary sensitivity analysis using prespecified hyperparameter tuning applied equally to both labeling strategies. The tuned-model results yielded conclusions consistent with those of the primary analysis, indicating that the main findings were robust to reasonable variations in hyperparameter selection.

To improve reproducibility, we have revised the Methods section to explicitly report the software versions (Python 3.12, scikit-learn 1.7.2, and XGBoost 3.2.0).

3. The manuscript should clarify whether feature scaling or normalization was performed, particularly for SVM and logistic regression.

Response: We thank the reviewer for this comment. We have clarified the implementation details of the machine learning models in the revised manuscript. Specifically, all classifiers were implemented using scikit-learn pipelines. Standardization was applied using StandardScaler prior to model training, and the classifiers were trained using the default hyperparameters provided by the corresponding software packages, except for the random seed, which was fixed to ensure reproducibility.

4. The authors should report whether class imbalance was addressed through class weights, resampling, or threshold adjustment. If no such methods were used, this should be stated.

Response: We thank the reviewer for this important comment. No class-weighting, resampling, or threshold-adjustment methods were applied in this study. All classifiers were trained using the original class distributions and default decision thresholds. To improve transparency, the class distributions for both labeling strategies have been added to Table 2. Because the degree of class imbalance was relatively moderate, we chose to evaluate all models under the original data distribution to ensure a consistent comparison of labeling strategies. We have clarified this point in the revised manuscript.

5. Confidence intervals should be provided for AUC, average precision, sensitivity, specificity, precision, and F1 score.

Response: We thank the reviewer for this valuable suggestion regarding statistical reporting and uncertainty estimation. In response, we additionally estimated 95% confidence intervals for all classification performance metrics, including accuracy, sensitivity, specificity, precision, F1 score, AUC, and average precision, using 1000 bootstrap resamples. The corresponding confidence intervals have been added to the revised tables.

In addition, the ROC curve figures were revised to incorporate the confidence interval information for AUC estimates. These revisions provide a more robust assessment of model performance and variability across labeling strategies.

6. Confusion matrix counts should also be shown, not only derived metrics.

Response: We thank the reviewer for this valuable suggestion. To provide the underlying classification results beyond the derived performance metrics, we have added confusion matrices for all machine learning models under both the Consensus- and Wiggs-based labeling strategies as Supplementary Figure 2. These matrices present the numbers of true positives, false positives, true negatives, and false negatives for each model, allowing a more detailed assessment of classification performance.

**Supplementary Figure 2.** Confusion matrices of machine learning models for detecting visual field progression according to two labeling strategies.
Confusion matrices for support vector machine (SVM), random forest, logistic regression, and XGBoost models are shown using the Consensus label and Wiggs’ label as reference standards. Rows indicate the true class labels and columns indicate the predicted class labels. Cell values represent the number of eye-episodes classified as stable or progressing.

7. The authors should explain how both eyes from the same patient were handled and whether within-patient correlation was considered.

Response: We thank the reviewer for this important comment. Both eyes were included when they met the eligibility criteria, and multiple eye-episodes could therefore originate from the same patient. We acknowledge that observations from fellow eyes of the same patient may not be fully independent and could introduce within-patient correlation.

To address the concern regarding within-patient correlation, we repeated the analysis using only one eye per patient. Accuracy, sensitivity, specificity, precision, and F1 score were calculated for both the Consensus- and Wiggs-based labeling strategies. The results were comparable to those of the primary analysis, indicating that the inclusion of both eyes did not substantially affect model performance.

|  |  | Training data  (n = 4614) | Test data  (n = 1142) |
| --- | --- | --- | --- |
| Dataset characteristics | Age (year), mean $\pm$ SD | 57.104 ± 14.4 | 57.021 ± 14.561 |
|  | The intervals between visual field examinations (year), mean $\pm$ SD | 1.94 ± 0.552 | 1.962 ± 0.567 |
|  | Consensus label: stable / progressing, n (%) | 3247 (70.4%) / 1367 (29.6%) | 811 (71.0%) / 331 (29.0%) |
|  | Wiggs’ label: stable / progressing, n (%) | 2860 (62.0%) / 1754 (38.0%) | 717 (62.8%) / 425 (37.2%) |
| Features used for Machine Learning | AGIS, mean ± SD | 0.192 ± 1.811 | 0.147 ± 1.871 |
|  | CIGTS, mean ± SD | −0.011 ± 2.619 | −0.08 ± 2.568 |
|  | MD, mean ± SD | −0.153 ± 1.915 | −0.122 ± 2.027 |
|  | MD slope, mean ± SD | −0.142 ± 0.449 | −0.137 ± 0.448 |
|  | VFI, mean ± SD | −1.142 ± 5.613 | −0.908 ± 6.14 |
|  | VFI slope, mean ± SD | −0.494 ± 1.553 | −0.476 ± 1.602 |
|  | Mean 52 TDV, mean ± SD | −0.151 ± 1.963 | −0.119 ± 2.048 |
|  | Mean 52 PDV, mean ± SD | −0.244 ± 1.203 | −0.201 ± 1.225 |

|  |  | Accuracy  (95% CI) | Sensitivity  (95% CI) | Specificity  (95% CI) | Precision  (95% CI) | F1 score  (95% CI) |
| --- | --- | --- | --- | --- | --- | --- |
| Consensus label | SVM | 0.934 (0.920-0.948) | 0.782 (0.737-0.827) | 0.996 (0.991-1.000) | 0.989 (0.974-1.000) | 0.874 (0.844-0.902) |
|  | RF | 0.940 (0.926-0.954) | 0.804 (0.761-0.845) | 0.995 (0.990-0.999) | 0.985 (0.970-0.996) | 0.885 (0.859-0.911) |
|  | LR | 0.930 (0.915-0.944) | 0.764 (0.719-0.810) | 0.998 (0.994-1.000) | 0.992 (0.980-1.000) | 0.863 (0.833-0.893) |
|  | XGBoost | 0.939 (0.925-0.953) | 0.813 (0.769-0.854) | 0.990 (0.983-0.996) | 0.971 (0.951-0.989) | 0.885 (0.859-0.910) |
| Wiggs’ label | SVM | 0.803 (0.779-0.827) | 0.642 (0.595-0.689) | 0.898 (0.876-0.919) | 0.789 (0.747-0.828) | 0.708 (0.672-0.744) |
|  | RF | 0.791 (0.766-0.815) | 0.649 (0.601-0.695) | 0.874 (0.851-0.899) | 0.754 (0.712-0.797) | 0.698 (0.662-0.734) |
|  | LR | 0.793 (0.770-0.818) | 0.595 (0.548-0.642) | 0.911 (0.890-0.931) | 0.798 (0.751-0.840) | 0.682 (0.644-0.720) |
|  | XGBoost | 0.792 (0.769-0.816) | 0.682 (0.639-0.726) | 0.858 (0.834-0.884) | 0.740 (0.698-0.783) | 0.710 (0.677-0.745) |
