# Supplementary material for: Labeling matters: A multicenter machine learning study on visual field progression in Glaucoma
Source: PLoS One. 2026 Aug 28;21(8):e0357179. doi: 10.1371/journal.pone.0357179 (PMC13524274; doi:10.1371/journal.pone.0357179)
Supplement: Supplementary file 14 [file pone.0357179.s017.docx]

**Major comments**

**1. The predictor set is still not reported consistently**: The Methods state that 10 machine-learning input features were used, including the mean and standard deviation of TDV and PDV. However, Table 2 still reports only Mean 52 TDV and Mean 52 PDV and does not explicitly report TDV standard deviation and PDV standard deviation as separate input features. Since these variables appear to be part of the classifier input set, Table 2 should be revised to include all 10 input features clearly, or the Methods should be corrected if these variables were not included in the final models.

Response: We thank the reviewer for this comment. To improve consistency between the Methods section and Table 2, we have revised Table 2 to explicitly list all 10 input features used in the model training. Specifically, the standard deviation variables for TDV and PDV, which were included in the classifier input set, are now listed as separate entries in the table.

Table 2. Demographic and visual field–derived features used as machine learning inputs in the training and test sets. Age and the interval between visual field examinations are reported as the mean and standard deviation for each eye-episode, whereas all other variables are presented as the mean and standard deviation of the change values, calculated as the difference between the late-phase and early-phase averages within each eye-episode.

|  |  | Training data  (n = 7611) | Test data  (n = 1903) |
| --- | --- | --- | --- |
| Dataset characteristics | Age (year), mean $\pm$ SD | 56.755 ± 14.484 | 56.656 ± 13.932 |
|  | The intervals between visual field examinations (year), mean $\pm$ SD | 1.933 ± 0.546 | 1.924 ± 0.536 |
|  | Initial MD ≥ −6 dB | 7536 | 1886 |
|  | −6 dB > Initial MD ≥ −12 dB | 68 | 16 |
|  | −12 dB > Initial MD | 7 | 1 |
|  | Consensus label: stable / progressing, n (%) | 5333 (70.1%) / 2278 (29.9%) | 1334 (70.1%) / 569 (29.9%) |
|  | Wiggs’ label: stable / progressing, n (%) | 4814 (63.3%) / 2797 (36.7%) | 1203 (63.2%) / 700 (36.8%) |
|  | Center balance, n (%) | Dong-A:3262 (42.9%),  Busan Paik : 979 (12.9%), Kosin : 236 (3.1%),  Pusan : 2627 (34.5%),  Yangsan : 507 (6.7%) | Dong-A : 784 (41.2%),  Busan Paik : 247 (13.0%),  Kosin : 68 (3.6%),  Pusan : 666 (35.0%),  Yangsan : 138 (7.3%) |
| Features used for machine learning | AGIS, mean $\pm$ SD | 0.179 ± 1.744 | 0.176 ± 1.718 |
|  | CIGTS, mean $\pm$ SD | 0.012 ± 2.561 | −0.006 ± 2.533 |
|  | MD, mean $\pm$ SD | −0.154 ± 1.87 | −0.149 ± 1.81 |
|  | MD slope, mean $\pm$ SD | −0.14 ± 0.429 | −0.129 ± 0.394 |
|  | VFI, mean $\pm$ SD | −1.026 ± 5.408 | −1.08 ± 5.239 |
|  | VFI slope, mean $\pm$ SD | −0.469 ± 1.466 | −0.427 ± 1.373 |
|  | Mean 52 TDV mean, mean $\pm$ SD | −0.149 ± 1.905 | −0.143 ± 1.861 |
|  | Mean 52 TDV SD, mean $\pm$ SD | 2.52 ± 1.419 | 2.504 ± 1.382 |
|  | Mean 52 PDV mean, mean $\pm$ SD | −0.21 ± 1.16 | −0.231 ± 1.133 |
|  | Mean 52 PDV SD, mean $\pm$ SD | 2.519 ± 1.419 | 2.503 ± 1.383 |

**2. The feature construction procedure requires clearer description:** The Methods state that each eye-episode was divided into an early phase and a late phase, that mean values of 10 features were calculated separately for the two phases, and that the late-minus-early difference was used as the classifier input. However, this description is not fully clear for slope-based variables such as MD slope and VFI slope, which are usually calculated as trend estimates over time rather than simple early/late mean values. The authors should define exactly how each of the 10 features was computed, particularly MD slope, VFI slope, TDV standard deviation, and PDV standard deviation, and clarify whether each feature represents a late-minus-early difference, a full-episode slope, or another summary measure.

Response: We revised the Methods section as follows.

(Original) For each eye-episode, the mean values of 10 features were calculated separately for the early and late phases. The difference between the late-phase means and the early-phase means (late minus early) was used as the feature value and served as the input to the classifier. The features used in this study were: the AGIS score, CIGTS score, MD, MD slope, VFI, VFI slope, and the mean and standard deviation of TDV and PDV for each eye-episode. Of all eye-episodes, 80% were used as the training dataset and the remaining 20% were used as the test dataset.

(Revised) For AGIS, CIGTS, MD, and VFI, the feature values were calculated as the differences between the late-phase and early-phase means. For TDV and PDV, the mean values for the early and late phases were first calculated at each of the 52 visual field locations. Pointwise changes were then obtained by subtracting the early-phase mean from the late-phase mean at each location. The mean and standard deviation of these pointwise changes across the 52 locations were calculated for each eye-episode. MD slope and VFI slope were calculated by linear regression using the five consecutive visual field examinations within each eye-episode, with time in years from the first examination in the episode as the independent variable. The features used in this study were the AGIS score, CIGTS score, MD, MD slope, VFI, VFI slope, and the mean and standard deviation of TDV and PDV for each eye-episode. Of all eye-episodes, 80% were used as the training dataset and the remaining 20% were used as the test dataset.

**3. The data availability statement remains inconsistent:** The authors’ response explains that the complete raw multicenter clinical dataset cannot be publicly released because of ethical and institutional restrictions, while processed train/test data, sample data, and analysis code are available through GitHub. However, the submitted data availability information still indicates that all data are fully available without restriction. This discrepancy should be corrected. The final data availability statement should clearly distinguish restricted raw multicenter clinical data from publicly available processed datasets, sample data, and analysis code.

Response: We thank the reviewer for identifying this discrepancy. The selection of “all data are fully available without restriction” during the initial submission resulted from our misunderstanding of the submission question. We interpreted this option to mean that all materials that could be ethically and legally shared would be made publicly available, rather than that the raw multicenter clinical data were available without restriction.

The raw multicenter clinical data contain protected patient information and therefore cannot be publicly released because of institutional and ethical restrictions. In contrast, the processed train/test datasets, de-identified sample data, and analysis code are publicly available through our GitHub repository.

We have revised the Data Availability Statement in the manuscript to clearly distinguish the restricted raw clinical data from the publicly available processed datasets, sample data, and analysis code.

In addition, we confirmed that the submission system does not allow authors to modify the originally selected Data Availability option after submission. Therefore, we have contacted the journal and requested that the submission record be corrected accordingly, and we are currently awaiting their response.

“The raw multicenter clinical data are not publicly available because of institutional and ethical restrictions protecting patient privacy. Processed train/test datasets, sample data, and analysis code used in this study are publicly available through the following GitHub repository:

https://github.com/kimhb1029/Visual_Field_Progression”

**4. The public repository should be updated and aligned with the revised manuscript:** The authors should ensure that the shared code, notebooks, and outputs reproduce the revised tables and analyses as presented in the manuscript. If some parts of the preprocessing workflow cannot be reproduced because the raw clinical data are restricted, this limitation should be stated clearly in both the repository and the manuscript. The repository should also clearly document which parts of the revised analysis can be reproduced from the shared materials, including bootstrap confidence intervals, patient-level split analysis, leave-one-center-out validation, and any hyperparameter-tuning sensitivity analysis. Any outputs in the public repository should also be updated to match the corrected values in the revised manuscript.

Response: We thank the reviewer for this important comment regarding the public repository and the reproducibility of the revised analyses. We acknowledge that the previously shared repository no longer reflected the current version of the manuscript because substantial revisions and additional analyses were introduced during the revision process.

To avoid confusion arising from inconsistencies between the repository and the revised manuscript, we temporarily removed the outdated repository contents while preparing the revised version. The repository has now been updated to align with the revised manuscript, including the revised code, notebooks, and outputs corresponding to the reported analyses.

Because the raw multicenter clinical data cannot be publicly shared owing to institutional and patient privacy restrictions, this limitation is explicitly stated in both the manuscript and the repository documentation. The repository also clearly specifies which analyses can be reproduced using the publicly available processed datasets, sample data, and analysis code, and which components require access to the original clinical dataset.

**5. The concern about overlap between label construction and predictor variables remains only partially resolved:** The added correlation and ablation analyses are useful and improve the manuscript. However, the primary models still use features that are closely aligned with the label-defining criteria. Therefore, the reported high performance should not be interpreted as evidence of clinical ground-truth validity. The manuscript should state more clearly that the results primarily reflect the learnability and internal consistency of algorithm-derived functional labels within the selected feature space.

Response: We agree with the reviewer that, because the predictor variables are closely related to the criteria used to construct the algorithm-derived labels, the high classification performance observed in this study should not be interpreted as evidence of the clinical validity of either label. Rather, the observed performance primarily reflects the relative learnability and internal consistency of the algorithm-derived functional progression labels within the selected feature space.
Accordingly, we added the following sentence to the Discussion:

- The present results should not be interpreted as demonstrating the clinical validity of either label. Rather, they demonstrate the relative learnability and internal consistency of two algorithm-derived functional progression labels within the selected feature space.

**6. The ground-truth terminology and interpretation remain too strong in places:** Phrases such as “optimized ground truth,” “reliable ground truth,” and “true glaucomatous progression” remain stronger than the study design supports, given the absence of independent expert adjudication, structural OCT validation, or prospective clinical outcome validation. These phrases should be replaced with more precise wording such as “algorithm-derived functional reference label” or “functional progression label.”

Response: We thank the reviewer for this important observation. We agree that terms such as "optimized ground truth," "reliable ground truth," and "true glaucomatous progression" imply a degree of validation that our study design does not provide. Because these labels were generated algorithmically from functional (visual field) data, describing them as a definitive ground truth overstates their epistemic status.

Accordingly, we have revised the terminology throughout the manuscript. All instances of "optimized/reliable ground truth" and "true glaucomatous progression" have been replaced with "algorithm-derived functional reference label" and “glaucomatous progression”.

Response: We thank the reviewer for this valuable comment. As suggested, we have revised the terminology throughout the manuscript.

**7. The term “Consensus label” remains potentially misleading:** The proposed label is defined as progression when at least one of five algorithms identifies progression. This is an inclusive any-positive composite rule rather than a consensus rule in the usual sense of agreement among methods. Because the term “Consensus label” may imply stronger agreement or clinical validity than the definition supports, the authors should either rename the label, for example as an “inclusive composite algorithmic label” or “any-positive algorithmic label,” or state more explicitly throughout the manuscript that the term refers to an inclusive composite definition rather than agreement among algorithms.

Response: We thank the reviewer for this precise and important observation. We fully agree that the term "consensus" conventionally implies agreement among methods, and that our label, defined as progression detected by at least one of five algorithms, is more accurately characterized as an inclusive OR rule rather than a majority-agreement rule.

We are also aware that prior studies by Saeedi et al. and Sabharwal et al. defined their reference standards in the opposite direction, requiring agreement among four of six algorithms ("majority progression"), which more closely corresponds to a conventional consensus definition. We therefore cannot invoke these studies to justify our use of the term "Consensus label," and we appreciate the reviewer's implicit caution on this point.

Rather than renaming the label, however, we would like to offer a methodological and clinical justification for retaining the term with a clarified definition, as the reviewer indicated this would be acceptable.

The rationale for adopting an any-positive rule in the present study differs fundamentally from the rationale underlying majority-based definitions. Saeedi et al. and Sabharwal et al. aimed to construct a high-specificity reference standard by requiring agreement among multiple algorithms, thereby minimizing false-positive labels in the training set. Our study, by contrast, was designed to address a different problem: the systematic loss of genuine progression signals that occurs when any single algorithm, or a majority rule, is applied to a dataset where inter-algorithm agreement is known to be poor (κ = 0.12–0.52). Because each algorithm captures a distinct pattern of VF deterioration, requiring agreement risks discarding true progression events that are detectable only by a subset of methods. In the context of an irreversible disease where false negatives carry greater clinical consequence than false positives, an any-positive rule represents a deliberate and clinically motivated labeling strategy.

We acknowledge, however, that "Consensus label" may mislead readers into assuming majority agreement. To resolve this ambiguity without abandoning the term, which we believe captures the intent of integrating signals across heterogeneous algorithms, we have revised the manuscript to explicitly define the label at first use as follows in the Introduction section:

"Unlike majority-based consensus approaches that require agreement among multiple algorithms, this any-positive rule was adopted on clinical grounds: given the irreversibility of glaucomatous vision loss, the cost of a missed progression event was judged to outweigh the cost of a false-positive alert."

**8. The manuscript should clarify whether performance comparisons are descriptive or inferential:** The authors added confidence intervals for several performance metrics, which is helpful. However, the manuscript should clearly state whether AUC and average precision confidence intervals were calculated and where they are reported. In addition, if no formal statistical comparison between labeling strategies was performed, the comparison should be described as descriptive rather than inferential.

Response: We thank the reviewer for this helpful comment. We agree that the manuscript should clearly distinguish descriptive performance comparisons from formal statistical comparisons.

In the Methods section, we added the following sentence:

- 95% confidence intervals were calculated for AUC and AP. Because no formal statistical comparisons between labeling strategies or classifiers were performed, performance comparisons were interpreted descriptively.

In the Results section, we added the following sentences:

- Receiver Operating Characteristic (ROC) and Precision–Recall (PR) analyses for the Consensus label, including 95% confidence intervals for AUC and average precision (AP), are presented in Figure 3.
- ROC and PR analyses for the Wiggs’ label, including 95% confidence intervals for AUC and AP, are presented in Figure 4.

Accordingly, we revised the relevant descriptions in the manuscript.

- (In the Results) provided the greatest discriminative performance => showed the highest observed AUC values
- (In the Results) provided the greatest discriminative capability => showed the highest observed AUC
- (In the Discussion) The superior AUC and precision–recall performance … => The higher observed AUC and precision–recall values…

**Minor comments**

1. The Discussion still states that the models were compared with “only the labeling strategy varied.” This wording should be revised because changing the labeling strategy also changes positive class prevalence and likely the difficulty of the classification task. A more accurate statement would be that the same dataset, input feature set, and model architectures were used, while label definition, class balance, and classification difficulty differed between the two labeling approaches.

Response: We thank the reviewer for this comment. We agree that the original wording could be misleading. We revised the Discussion to clarify that the models were trained using the same dataset, input features, and model architecture, but with different ground-truth labeling strategies.

(Original) By comparing models trained on the same dataset and architecture with only the labeling strategy varied, this study demonstrates that the definition of ground truth is a critical determinant of performance in machine learning–based progression detection.

(Revised) By comparing models trained on the same dataset, input feature set, and model architecture using different ground-truth labeling strategies, this study demonstrates that the definition of ground truth is a critical determinant of performance in machine learning–based progression detection. Because different labeling strategies inherently result in differences in class prevalence and classification difficulty, the observed performance differences reflect the impact of alternative ground-truth definitions under otherwise consistent experimental conditions.

2. The authors added class balance across the training and test sets, which is helpful. However, my previous comment also requested center balance and disease severity balance across the split. These should either be reported, preferably in Table 2 or a supplementary table, or the authors should explain why they are not available.

Response: We thank the reviewer for this helpful suggestion. We have revised Table 2 to include the center distribution and disease severity distribution for both the training and test sets. This information allows for a clearer assessment of the comparability of the two datasets with respect to center composition and disease severity.

Table 2. Demographic and visual field–derived features used as machine learning inputs in the training and test sets. Age and the interval between visual field examinations are reported as the mean and standard deviation for each eye-episode, whereas all other variables are presented as the mean and standard deviation of the change values, calculated as the difference between the late-phase and early-phase averages within each eye-episode.

|  |  | Training data  (n = 7611) | Test data  (n = 1903) |
| --- | --- | --- | --- |
| Dataset characteristics | Age (year), mean $\pm$ SD | 56.755 ± 14.484 | 56.656 ± 13.932 |
|  | The intervals between visual field examinations (year), mean $\pm$ SD | 1.933 ± 0.546 | 1.924 ± 0.536 |
|  | Initial MD ≥ −6 dB | 7536 | 1886 |
|  | −6 dB > Initial MD ≥ −12 dB | 68 | 16 |
|  | −12 dB > Initial MD | 7 | 1 |
|  | Consensus label: stable / progressing, n (%) | 5333 (70.1%) / 2278 (29.9%) | 1334 (70.1%) / 569 (29.9%) |
|  | Wiggs’ label: stable / progressing, n (%) | 4814 (63.3%) / 2797 (36.7%) | 1203 (63.2%) / 700 (36.8%) |
|  | Center balance, n (%) | Dong-A:3262 (42.9%),  Busan Paik : 979 (12.9%), Kosin : 236 (3.1%),  Pusan : 2627 (34.5%),  Yangsan : 507 (6.7%) | Dong-A : 784 (41.2%),  Busan Paik : 247 (13.0%),  Kosin : 68 (3.6%),  Pusan : 666 (35.0%),  Yangsan : 138 (7.3%) |
| Features used for machine learning | AGIS, mean $\pm$ SD | 0.179 ± 1.744 | 0.176 ± 1.718 |
|  | CIGTS, mean $\pm$ SD | 0.012 ± 2.561 | −0.006 ± 2.533 |
|  | MD, mean $\pm$ SD | −0.154 ± 1.87 | −0.149 ± 1.81 |
|  | MD slope, mean $\pm$ SD | −0.14 ± 0.429 | −0.129 ± 0.394 |
|  | VFI, mean $\pm$ SD | −1.026 ± 5.408 | −1.08 ± 5.239 |
|  | VFI slope, mean $\pm$ SD | −0.469 ± 1.466 | −0.427 ± 1.373 |
|  | Mean 52 TDV mean, mean $\pm$ SD | −0.149 ± 1.905 | −0.143 ± 1.861 |
|  | Mean 52 TDV SD, mean $\pm$ SD | 2.52 ± 1.419 | 2.504 ± 1.382 |
|  | Mean 52 PDV mean, mean $\pm$ SD | −0.21 ± 1.16 | −0.231 ± 1.133 |
|  | Mean 52 PDV SD, mean $\pm$ SD | 2.519 ± 1.419 | 2.503 ± 1.383 |

3. The decision threshold or default prediction rule used to calculate sensitivity, specificity, precision, and F1 score should be stated explicitly in the Methods section, not only in the response letter.

Response: We revised the Methods section as follows.

(Original) Therefore, all models were trained using the default hyperparameters of each package, except for the fixed random seed.

(Revised) Therefore, all models were trained using the default hyperparameters of each package, except for the fixed random seed (random_state = 0). No class weighting, resampling, or threshold adjustment was applied, and the default decision threshold of 0.5 was used for binary classification.

4. The authors state that a fixed random seed was used, but the exact seed value should be reported in the Methods or Supplementary Materials to improve reproducibility.

Response: The exact random seed value used in this study (random_state = 0) has now been explicitly reported in the Methods section to improve reproducibility.

5. The authors should ensure that the repository language is consistent with the revised manuscript. Any statements suggesting that the model or labeling strategy supports fully generalizable clinical AI systems should be moderated unless supported by external validation beyond the included centers.

Response: We thank the reviewer for this important comment. We agree that the present study does not provide external validation beyond the participating centers and therefore does not support claims regarding fully generalizable clinical AI systems.

In the revised manuscript, we have avoided statements implying that the proposed models constitute clinically deployable or universally generalizable AI systems. The repository documentation will be updated accordingly to ensure that its language is consistent with the revised manuscript.

Specifically, the repository will emphasize that the present study investigates model-label compatibility and the influence of labeling strategies on machine learning performance, rather than validating a clinically deployable progression-detection system. The limitations regarding external validation and generalizability will also be explicitly stated.

6. The Methods and supplementary materials should clearly identify which analyses were part of the primary revision and which were sensitivity analyses. This is particularly important for the patient-level split, leave-one-center-out validation, bootstrap confidence intervals, and any hyperparameter-tuning analysis.

Response: We thank the reviewer for this helpful suggestion. Following the comments from multiple reviewers, several additional analyses were incorporated into the revised manuscript and Supplementary Materials. We agree that the increased number of supplementary tables may make it more difficult to distinguish the primary analysis from additional analyses.

To improve clarity, the supplementary tables have now been organized and explicitly identified according to their analytical purpose as follows:

- Supplementary Tables 1–5: Feature ablation analyses (sensitivity analyses)
- Supplementary Table 6: Correlation between input features and progression labels
- Supplementary Tables 7–9: Patient-level train–test split analyses (sensitivity analyses)
- Supplementary Table 10: Leave-one-center-out validation analysis (sensitivity analysis)

In addition, the titles of Supplementary Tables 7–10 have been revised to explicitly indicate their roles as additional or sensitivity analyses.

Although these results were not included in the supplementary materials, the results of the hyperparameter tuning analysis have been added below. The hyperparameter search ranges and the corresponding tuned-model performance results are summarized as follows:

| Model | Hyperparameters |
| --- | --- |
| SVM | C: 0.1, 1, 10, 100; gamma: scale, 0.01, 0.1; kernel: rbf |
| Random Forest | n_estimators: 100, 300; max_depth: None, 10, 20; min_samples_split: 2, 5 |
| Logistic Regression | C: 0.01, 0.1, 1, 10, 100 |
| XGBoost | n_estimators: 100, 300; max_depth: 3, 5, 7; learning_rate: 0.01, 0.1; subsample: 0.8, 1.0 |

|  | | Accuracy | Sensitivity | Specificity | Precision | F1 score |
| --- | --- | --- | --- | --- | --- | --- |
|  |  | (95% CI) | | | | |
| Consensus Tuned | SVM | 0.951  (0.940-0.960) | 0.837  (0.804-0.864) | 0.999  (0.998-1.000) | 0.998  (0.994-1.000) | 0.910  (0.890-0.926) |
|  | RF | 0.955  (0.945-0.963) | 0.851  (0.819-0.878) | 0.999  (0.998-1.000) | 0.998  (0.994-1.000) | 0.918  (0.900-0.934) |
|  | LR | 0.951  (0.941-0.960) | 0.838  (0.807-0.866) | 0.999  (0.996-1.000) | 0.996  (0.989-1.000) | 0.910  (0.891-0.927) |
|  | XGBoost | 0.953  (0.943-0.962) | 0.844  (0.811-0.872) | 0.999  (0.998-1.000) | 0.998  (0.994-1.000) | 0.914  (0.894-0.930) |
| Wiggs Tuned | SVM | 0.826  (0.809-0.843) | 0.660  (0.624-0.694) | 0.922  (0.907-0.936) | 0.831  (0.802-0.859) | 0.736  (0.707-0.761) |
|  | RF | 0.823  (0.806-0.839) | 0.700  (0.667-0.732) | 0.894  (0.877-0.912) | 0.794  (0.763-0.825) | 0.744  (0.717-0.767) |
|  | LR | 0.811  (0.794-0.827) | 0.630  (0.594-0.665) | 0.916  (0.901-0.932) | 0.814  (0.782-0.845) | 0.710  (0.683-0.737) |
|  | XGBoost | 0.815  (0.797-0.831) | 0.683  (0.647-0.717) | 0.892  (0.873-0.910) | 0.786  (0.754-0.818) | 0.731  (0.704-0.756) |

The tuned models showed only minor changes in absolute performance compared with the default-parameter models. Importantly, the relative performance pattern remained unchanged, with models trained using the Consensus label consistently showing higher observed performance than those trained using the Wiggs label across classifiers. These findings suggest that the primary conclusions of the study are robust to reasonable variations in hyperparameter selection and are not primarily driven by default model settings.

7. The preprocessing flow has improved, but the authors should ensure that Supplementary Figure 1 reports the number of visual field tests, eyes, patients, segments, and eye-episodes retained or excluded at each major filtering step, including the segmentation and five-visit episode construction steps.

Response: Supplementary Figure 1 has been revised to provide the numbers of visual field examinations. Specifically, the number of visual field examinations remaining after reliability filtering, exclusion of the first visual field test for each eye, and selection of eyes with at least five visual field examinations are now explicitly reported.

The segmentation procedure and five-visit eye-episode construction process are illustrated in Figures 1 and 2, respectively. Together, these figures provide a complete description of the preprocessing workflow and the derivation of the final analytical dataset.
