# Supplementary material for: Labeling matters: A multicenter machine learning study on visual field progression in Glaucoma
Source: PLoS One. 2026 Aug 28;21(8):e0357179. doi: 10.1371/journal.pone.0357179 (PMC13524274; doi:10.1371/journal.pone.0357179)
Supplement: Peer review history

# Peer review: Decision Letter 0

**DOI:** 10.1371/journal.pone.0357179.r001

**Authors:** Panos Liatsis

1 May 2026

Dear Dr. Lee,

Thank you for submitting your manuscript to PLOS ONE. After careful consideration, we feel that it has merit but does not fully meet PLOS ONE’s publication criteria as it currently stands. Therefore, we invite you to submit a revised version of the manuscript that addresses the points raised during the review process.

- Task framing is overstated. The study is presented as prediction / early-warning detection, but the design is concurrent within-episode classification (labels and inputs both drawn from the same five-visit window) and lacks an independent reference standard (OCT, expert adjudication, longitudinal outcomes). Claims of clinical reliability and generalizability should be better supported.
- There is circularity between labels and features. The Consensus label is constructed from AGIS, CIGTS, MD slope, VFI slope, and PLR while the model inputs include the same or closely related quantities. The ablation does not resolve this, and a leakage-reduced sensitivity analysis using features independent of label definition is needed.
- The Consensus terminology and label comparison are misleading. The label is an inclusive OR rule, not a consensus. The Consensus-vs-Wiggs comparison is also confounded by feature representation and by differing class prevalence, so lower Wiggs performance cannot be read as label inferiority.
- The validation strategy is insufficient for the claims made. Train/test split unit (patient/eye/episode) is unclear and risks leakage, while multicenter generalizability is asserted without center-held-out or leave-one-center-out validation. There are no confidence intervals, repeated resampling, or formal between-label/between-model comparisons. The hyperparameter choice is not justified equally across labels.
- There are reporting, reproducibility, and transparency gaps. There are inconsistencies between Methods and Table 2 on the predictor set (TDV/PDV SDs, age, interval), lack of clarity wrt feature scaling, class-imbalance handling, and decision threshold. There are missing confusion matrices, hyperparameters, and package versions. Data-availability statement contradicts the repository contents. Follow-up duration vs. inter-test interval is inconsistent across tables while regional-partition description for Wiggs label is ambiguous.
- The reference list is short and dated. The introduction and discussion should be extended with recent work, and the paper should acknowledge that the labeling-strategy argument generalises beyond glaucoma. Acquisition devices/test types per center should also be reported in Tables 1–2.

==============================

Please submit your revised manuscript by Jun 15 2026 11:59PM. If you will need more time than this to complete your revisions, please reply to this message or contact the journal office at plosone@plos.org. When you're ready to submit your revision, log on to https://www.editorialmanager.com/pone/ and select the 'Submissions Needing Revision' folder to locate your manuscript file.

Please include the following items when submitting your revised manuscript:

- A letter that responds to each point raised by the academic editor and reviewer(s). You should upload this letter as a separate file labeled 'Response to Reviewers'.
- A marked-up copy of your manuscript that highlights changes made to the original version. You should upload this as a separate file labeled 'Revised Manuscript with Track Changes'.
- An unmarked version of your revised paper without tracked changes. You should upload this as a separate file labeled 'Manuscript'.

If you would like to make changes to your financial disclosure, please include your updated statement in your cover letter. Guidelines for resubmitting your figure files are available below the reviewer comments at the end of this letter.

If applicable, we recommend that you deposit your laboratory protocols in protocols.io to enhance the reproducibility of your results. Protocols.io assigns your protocol its own identifier (DOI) so that it can be cited independently in the future. For instructions see: https://journals.plos.org/plosone/s/submission-guidelines#loc-laboratory-protocols. Additionally, PLOS ONE offers an option for publishing peer-reviewed Lab Protocol articles, which describe protocols hosted on protocols.io. Read more information on sharing protocols at https://plos.org/protocols?utm_medium=editorial-email&utm_source=authorletters&utm_campaign=protocols.

As the corresponding author, your ORCID iD is verified in the submission system and will appear in the published article. PLOS supports the use of ORCID, and we encourage all coauthors to register for an ORCID iD and use it as well. Please encourage your coauthors to verify their ORCID iD within the submission system before final acceptance, as unverified ORCID iDs will not appear in the published article. *Only*  the individual author can complete the verification step; PLOS staff *cannot*  verify ORCID iDs on behalf of authors.

We look forward to receiving your revised manuscript.

Kind regards,

Panos Liatsis, PhD

Academic Editor

PLOS One

**Journal Requirements:**

When submitting your revision, we need you to address these additional requirements.

1. Please ensure that your manuscript meets PLOS ONE's style requirements, including those for file naming. The PLOS ONE style templates can be found at

https://journals.plos.org/plosone/s/file?id=wjVg/PLOSOne_formatting_sample_main_body.pdf and

https://journals.plos.org/plosone/s/file?id=ba62/PLOSOne_formatting_sample_title_authors_affiliations.pdf

2. Please note that PLOS One has specific guidelines on code sharing for submissions in which author-generated code underpins the findings in the manuscript. In these cases, we expect all author-generated code to be made available without restrictions upon publication of the work. Please review our guidelines at https://journals.plos.org/plosone/s/materials-and-software-sharing#loc-sharing-code and ensure that your code is shared in a way that follows best practice and facilitates reproducibility and reuse.

3. Please note that funding information should not appear in any section or other areas of your manuscript. We will only publish funding information present in the Funding Statement section of the online submission form. Please remove any funding-related text from the manuscript.

4. Please include captions for your Supporting Information files at the end of your manuscript, and update any in-text citations to match accordingly. Please see our Supporting Information guidelines for more information: http://journals.plos.org/plosone/s/supporting-information.

5. If the reviewer comments include a recommendation to cite specific previously published works, please review and evaluate these publications to determine whether they are relevant and should be cited. There is no requirement to cite these works unless the editor has indicated otherwise.

[Note: HTML markup is below. Please do not edit.]

Reviewers' comments:

Reviewer's Responses to Questions

**Comments to the Author**

1. Is the manuscript technically sound, and do the data support the conclusions?

Reviewer #1: Partly

Reviewer #2: Yes

Reviewer #3: Partly

**********

2. Has the statistical analysis been performed appropriately and rigorously?

Reviewer #1: No

Reviewer #2: Yes

Reviewer #3: No

**********

3. Have the authors made all data underlying the findings in their manuscript fully available?

The PLOS Data policy

Reviewer #1: No

Reviewer #2: Yes

Reviewer #3: No

**********

4. Is the manuscript presented in an intelligible fashion and written in standard English?

Reviewer #1: Yes

Reviewer #2: No

Reviewer #3: Yes

**********

Reviewer #1: Comments to authors

Recommendation: Major revision

This manuscript addresses a clinically relevant and methodologically important question: how ground-truth labeling strategy affects machine learning performance for detecting visual field progression in glaucoma. The multicenter design, relatively large sample size, and direct comparison of two labeling approaches are clear strengths. The study also raises an issue of broad relevance in medical AI, namely that label definition can materially influence downstream model performance. However, several aspects of the current analytic design and interpretation limit the strength of the conclusions, and these issues should be addressed before the manuscript can be considered for acceptance.

Major comments

1. The study is framed as prediction, but the present design appears closer to retrospective within-window classification: The unit of analysis is a 5-visit eye-episode. The labels are defined within that same episode, and the model inputs are also derived from the same episode by comparing the first two visits with the last three visits. In particular, the Wiggs label explicitly uses the final visit or final two visits in its definition. Because late-episode information contributes both to the target and to the predictor set, the present design appears to support retrospective classification of already observed progression within a fixed window more than true future prediction. Accordingly, claims such as “early progression detection” and “early warning systems” should be moderated unless the authors can demonstrate a strictly forward-looking prediction framework.

2. There is substantial overlap between label construction and model inputs, raising a serious concern about circularity: The Consensus label is defined by AGIS, CIGTS, MD slope, VFI slope, and PLR. However, the model inputs also include AGIS, CIGTS, MD, MD slope, VFI, VFI slope, and TDV/PDV-based summary variables. This means that the classifiers are trained using predictors that substantially overlap with the target definition itself. The ablation analysis is useful, but it does not fully resolve the core concern because the principal reported model performances still rely on overlapping labels and features. At minimum, this issue should be discussed more explicitly. Ideally, the authors should provide a sensitivity analysis using features that are independent of the criteria used to define the labels.

3. The manuscript should clarify the train-test split in enough detail to rule out patient-level or eye-level leakage: The paper states that the unit of analysis is the eye-episode, that 9,514 eye-episodes from 4,496 patients were analyzed, and that 80% of eye-episodes were assigned to training and 20% to testing. However, it is not stated whether the split was grouped by patient or by eye. If multiple episodes from the same eye, or both eyes from the same patient, were allowed to appear in both training and test sets, the reported performance could be optimistic. The authors should clarify this explicitly and, preferably, provide a grouped patient-level split as a sensitivity analysis.

4. The multicenter nature of the dataset is a strength, but the current validation strategy does not fully support the manuscript’s generalizability claims: The manuscript emphasizes heterogeneous clinical settings and concludes that the proposed labeling strategy supports robust and generalizable AI systems. However, the Methods describe a single 80/20 split rather than center-held-out validation or a leave-one-center-out design. If data from the same institutions appear in both training and test sets, institution-specific patterns may be shared across splits. A center-level validation strategy would provide much stronger support for the generalizability claims, or alternatively the wording in the Abstract and Discussion should be moderated.

5. The reporting of the predictor set is internally inconsistent and should be corrected: The Methods state that the model uses 10 features, including the mean and standard deviation of TDV and PDV. However, Table 2 lists age, interval between examinations, AGIS, CIGTS, MD, MD slope, VFI, VFI slope, Mean 52 TDV, and Mean 52 PDV, but does not show the standard deviation of TDV or the standard deviation of PDV. In addition, age and interval are described in Table 2 as machine learning inputs, yet they are not included in the explicit list of 10 features in the Methods. The authors should reconcile the Methods and Table 2 so that the exact predictor set is unambiguous.

6. The statistical reporting should be strengthened with uncertainty estimates and clearer comparison of labeling strategies: The paper reports point estimates for accuracy, sensitivity, specificity, precision, F1 score, AUC, and average precision, but I did not see confidence intervals, repeated resampling, or formal statistical comparisons between models or between the two labeling strategies. Because the central claim is that label definition materially affects model performance, uncertainty around these differences should be quantified. Bootstrapped confidence intervals and appropriate comparisons for AUC and threshold-based metrics would strengthen the manuscript considerably.

7. The Introduction is generally clear, but several framing claims should be moderated or better justified: The Introduction appropriately summarizes the problem of low agreement among visual field progression algorithms and motivates the study question. However, the novelty claim that no previous study has systematically compared how labeling strategy affects machine learning performance should be phrased more cautiously, especially because the manuscript itself cites prior consensus-based work. In addition, the rationale for defining the proposed Consensus label as positivity under any one of five criteria is underdeveloped, particularly given that the cited prior consensus-based approach used a much stricter threshold. Finally, the phrase “optimized ground truth” appears stronger than the present study design can support, because the labels are not externally validated against structural OCT, expert adjudication, or long-term clinical outcomes.

Minor comments

1. The interpretation of the Wiggs label as “stricter” or more “conservative” requires clearer explanation, because its observed positive rate is higher than that of the Consensus label. This may be explainable, but it is counterintuitive and should be discussed explicitly so that readers can reconcile the narrative with the empirical class distribution.

2. The data availability statement should be clarified. The submission materials state that all data are fully available without restriction and that the data and code supporting the findings are publicly available in a GitHub repository. However, the repository description indicates that the original dataset cannot be publicly released and that only preprocessed train and test data are provided. The authors should specify precisely what is available, what is restricted, and whether the shared materials are sufficient for independent reproduction of the reported analyses.

3. The reporting of follow-up timing appears internally inconsistent and should be clarified. Table 1 reports a mean follow-up duration of 1.53 years and exactly 5.0 visual field tests per eye-episode, whereas Table 2 reports the interval between visual field examinations as approximately 1.93 years in both training and test sets. As currently labeled, these quantities do not appear to be compatible and may reflect a unit error, mislabeled variable, or reporting inconsistency.

4. There appears to be an incorrect table citation in the Methods section. In the description of the Wiggs-based criteria, the manuscript refers to Table 3, whereas the Wiggs-related counts appear in Table 5. This should be corrected for clarity.

5. Please clarify the decision threshold used for the reported sensitivity, specificity, precision, and F1 score values. The manuscript refers to a fixed decision threshold, but the threshold itself is not clearly specified.

6. Please provide complete implementation details for all models, especially XGBoost, including package versions and key hyperparameters.

7. The preprocessing workflow would be easier to evaluate if the manuscript quantified how many eyes, segments, or episodes were excluded at each filtering step.

8. Table 2’s independent t-tests comparing the random train and test splits are not especially informative. It would be more useful to report class balance, center balance, and disease severity balance across the two splits.

9. The limitation that the Consensus label reflects only functional progression and is not linked to structural OCT evidence is important and should be emphasized clearly, potentially earlier in the manuscript.

10. The description of the regional partition used for the Wiggs label is not fully clear. The Methods state that pattern deviation plots were divided into six peripheral regions and two paracentral regions, but the parenthetical description appears to name only superior and inferior nasal step and superior and inferior Bjerrum areas. Please describe the regional partition unambiguously.

11. The Discussion states that the models were compared on the same dataset and architecture with “only the labeling strategy varied.” This wording appears somewhat too strong, because the positive class prevalence also differs between the Consensus and Wiggs labels. The authors should revise this sentence to reflect that the comparison changes not only label definition but also class balance and likely the difficulty of the classification task.

Overall assessment

This is a potentially valuable study with an important question, a multicenter design, and a sizable curated dataset. The manuscript’s central idea, that labeling strategy itself can strongly influence machine learning results, is worthwhile. However, the current version overstates what can be concluded from the present design. The most important issues are the framing of the task as prediction rather than within-window classification, overlap between labels and predictors, unresolved risk of patient- or eye-level leakage, inconsistency in feature reporting, limited support for multicenter generalizability claims, and the need for clearer statistical reporting. For these reasons, I recommend major revision.

Reviewer #2: Dear Authors

Strength: Interesting to evaluate the ML performances once clinical labels differ (e.g. in assessing glaucoma). The paper proved statistically that generalizing AI models should take in account that clinical labels should be standardized. Additionally, the paper presents a research results on "large" datasets that obtained from multiple centers.

Major concern 1: Bibliography needs to be extended. Current references are relatively old. Therefore, it is recommended : 1-) update some already references with more recently published papers in the same context, and 2-) you need to add more references related to the title of this manuscript. After visiting google scholar, the following two links could be useful:

https://scholar.google.com/scholar?hl=en&as_sdt=0%2C5&as_ylo=2025&q=labeling+matters+in+ophthalmology+glaucoma&btnG=

https://scholar.google.com/scholar?as_ylo=2025&q=labeling+matters+in+ophthalmology&hl=en&as_sdt=0,5

After addressing this concern, it is recommended that you can extend the introduction and discussion sections accordingly to the new reference list.

It is good to tell the reader that title of this paper is NOT restricted to "glaucoma". Other applications also have the same argument.

In summary, the current 17 references, in my point of view, need to be remarkably extended along with their associated text specifically in the introduction section.

Major concern 2: In my point of view, it would be complementary correct to mention the machines (types of images or tests) used in diagnosing eyes in Tables 1 and 2, in each center whom participated in this study.

Best wishes

Reviewer #3: This is an interesting and potentially useful study addressing an important issue in glaucoma research: how the choice of progression label influences the apparent performance of machine-learning models. The multicenter dataset is a strength, and I appreciate the authors’ attempt to compare different labeling strategies under the same modeling framework. However, I think the current manuscript overstates what can be concluded from the analysis. In particular, the study appears to show how well different algorithm-derived labels can be reproduced from visual-field summary features, rather than validating a clinically reliable detector of true glaucomatous progression.

Major comments

1. The study addresses an important and underappreciated issue in glaucoma progression modeling: the extent to which the definition of the outcome label determines apparent machine-learning performance. However, the manuscript does not always keep this distinction clear. At several points, the study is presented as if it developed a clinically reliable detector of glaucomatous visual field progression, whereas the actual analysis mainly shows that one algorithm-derived label was more readily reproduced from selected visual-field summary features than another. The authors should more clearly frame the study as an investigation of label behavior and model-label compatibility, rather than as validation of a clinically useful progression-detection system.

2. I am concerned about the use of the term “Consensus label.” The proposed label is defined as progression according to at least one of five algorithms. This is an inclusive OR rule, not a consensus rule in the usual sense of agreement among methods. This point is not merely semantic, because the term “consensus” may imply greater clinical validity or reliability than the definition supports. The authors should either rename this label, for example as an “inclusive composite algorithmic label,” or provide a stronger justification for why a one-positive-rule should be regarded as a consensus definition.

3. The study lacks an independent reference standard for true glaucomatous progression. Both the Consensus label and the Wiggs’ label are derived from visual-field rules, and neither is validated here against masked expert adjudication, OCT or optic disc progression, subsequent visual-field worsening, treatment escalation, or another clinically independent endpoint. Therefore, the results cannot establish that either label is closer to true disease progression. Claims such as “clinically reliable detection,” “minimal false positives,” “clinically valid ground truth,” and “early warning systems” should be substantially toned down unless additional validation is provided.

4. There is a potentially serious circularity problem between the labels and the input features. The Consensus label is constructed from AGIS, CIGTS, MD slope, VFI slope, and PLR, while the model inputs include closely related quantities such as AGIS score, CIGTS score, MD, MD slope, VFI, VFI slope, and TDV/PDV summaries. Under this design, high AUC and specificity may largely reflect the model’s ability to reconstruct an algorithmic label from overlapping features. The current ablation analysis is useful, but it does not fully address this concern, because correlated features remain in the model and the target label still contains information derived from the same family of measurements. A leakage-reduced sensitivity analysis excluding all features directly or indirectly used to define each target label would be important.

5. The comparison between the Consensus and Wiggs’ labels may be confounded by the choice of input representation. The Consensus label is closely aligned with global and trend-based summary features, whereas the Wiggs’ label is based more on regional and spatially organized visual-field defects. If the model receives only hand-crafted summary features rather than the raw visual-field map or a richer regional representation, the Wiggs’ label may be disadvantaged by design. Thus, lower performance with the Wiggs’ label should not be interpreted as evidence that the label is inferior. The authors should discuss this issue explicitly and avoid equating predictability with clinical validity.

6. The validation strategy needs to be strengthened or, at minimum, described more transparently. The Methods indicate an 80:20 train–test split with a fixed random seed, but it is unclear whether the split was performed at the patient, eye, or eye-episode level. Because multiple eye-episodes can come from the same eye or patient, random episode-level splitting could inflate performance. The authors should clarify the splitting unit and provide patient-level or eye-level validation. Repeated cross-validation or bootstrapped confidence intervals would also make the performance estimates more credible. Since the study emphasizes a multicenter dataset, a leave-one-center-out analysis would be a particularly informative assessment of generalizability.

7. The decision not to tune hyperparameters is not fully convincing. Although the authors state that this was done to ensure an objective comparison, default or fixed parameters are not necessarily neutral across labels. The two labels may differ in class balance, noise structure, and decision boundaries, and one label may be better matched to default settings than the other. A prespecified tuning strategy applied equally to both labels, or at least a sensitivity analysis showing that the main conclusions are robust to tuning, would strengthen the methodological comparison.

8. The manuscript should distinguish more carefully between classifying progression within a five-test episode and predicting future progression. The models appear to use features derived from the same five visual fields that are used to assign the labels. This is a concurrent classification task, not a prospective prediction task. Therefore, statements about early warning or future clinical decision support are premature unless the authors perform an analysis in which earlier visual fields are used to predict progression occurring later.

Minor comments

1. The abstract should state more explicitly that both labels are algorithm-derived and that no independent clinical reference standard was used. This would prevent readers from overinterpreting the reported AUC and specificity.

2. The authors should report the exact hyperparameters used for each model, including the SVM kernel and regularization parameter, random forest depth and number of trees, and XGBoost learning rate, depth, number of estimators, and regularization settings.

3. The manuscript should clarify whether feature scaling or normalization was performed, particularly for SVM and logistic regression.

4. The authors should report whether class imbalance was addressed through class weights, resampling, or threshold adjustment. If no such methods were used, this should be stated.

5. Confidence intervals should be provided for AUC, average precision, sensitivity, specificity, precision, and F1 score. 7. 6. Confusion matrix counts should also be shown, not only derived metrics.

7. The authors should explain how both eyes from the same patient were handled and whether within-patient correlation was considered.

**********

what does this mean?). If published, this will include your full peer review and any attached files.

If you choose “no”, your identity will remain anonymous but your review may still be made public.

**Do you want your identity to be public for this peer review?**  For information about this choice, including consent withdrawal, please see our Privacy Policy

Reviewer #1: No

Reviewer #2: **Yes:** Abdel-Razzak Al-Hinnawi

Reviewer #3: No

**********

[NOTE: If reviewer comments were submitted as an attachment file, they will be attached to this email and accessible via the submission site. Please log into your account, locate the manuscript record, and check for the action link "View Attachments". If this link does not appear, there are no attachment files.]

To ensure your figures meet our technical requirements, please review our figure guidelines: https://journals.plos.org/plosone/s/figures

You may also use PLOS’s free figure tool, NAAS, to help you prepare publication quality figures: https://journals.plos.org/plosone/s/figures#loc-tools-for-figure-preparation.

NAAS will assess whether your figures meet our technical requirements by comparing each figure against our figure specifications.

Attachment Submitted filename: Comments to authors.pdf

# Author comment: Author response to Decision Letter 1

**DOI:** 10.1371/journal.pone.0357179.r002

8 Jun 2026

Reviewer #1:

Major comments

1. The study is framed as prediction, but the present design appears closer to retrospective within-window classification: The unit of analysis is a 5-visit eye-episode. The labels are defined within that same episode, and the model inputs are also derived from the same episode by comparing the first two visits with the last three visits. In particular, the Wiggs label explicitly uses the final visit or final two visits in its definition. Because late-episode information contributes both to the target and to the predictor set, the present design appears to support retrospective classification of already observed progression within a fixed window more than true future prediction. Accordingly, claims such as “early progression detection” and “early warning systems” should be moderated unless the authors can demonstrate a strictly forward-looking prediction framework.

Response: We thank the reviewer for this important methodological observation. We agree that the terms "early progression detection" and "early warning systems" imply a prospective, forward-looking prediction framework, which is not what the present study design supports. Our study is retrospective in nature: the machine learning models were trained and evaluated on labeled eye-episodes in which all five visual field examinations within each episode were available at the time of labeling. Accordingly, the models classify whether progression occurred within a completed series of observations, rather than predicting future progression from an incomplete or early time series.

In response, we have revised the relevant sentences in the Discussion section to more accurately reflect the retrospective, classification-based nature of our framework. Specifically, "early progression detection" has been replaced with "sensitive identification of progression," and "early warning systems" has been removed.

2. There is substantial overlap between label construction and model inputs, raising a serious concern about circularity: The Consensus label is defined by AGIS, CIGTS, MD slope, VFI slope, and PLR. However, the model inputs also include AGIS, CIGTS, MD, MD slope, VFI, VFI slope, and TDV/PDV-based summary variables. This means that the classifiers are trained using predictors that substantially overlap with the target definition itself. The ablation analysis is useful, but it does not fully resolve the core concern because the principal reported model performances still rely on overlapping labels and features. At minimum, this issue should be discussed more explicitly. Ideally, the authors should provide a sensitivity analysis using features that are independent of the criteria used to define the labels.

Response: As the reviewer appropriately pointed out, there is both a conceptual and statistical relationship between the criteria used to construct the Consensus label and some of the model input variables. Specifically, the Consensus label was defined based on rule-based progression indicators, including AGIS_test, CIGTS_test, MD_slope_test, VFI_slope_test, and PLR_test, whereas the model input variables included AGIS, CIGTS, MD, VFI, MD slope value, VFI slope value, and TDV/PDV-derived summary metrics. Therefore, it is possible that a portion of the model performance reflects information that is also related to the label definition.

However, we would like to emphasize that the label-defining variables and predictor variables used in this study were not direct duplicates of the same variables. The Consensus label was defined using binary rule-based indicators that determine whether specific progression criteria were met, whereas the model inputs consisted of continuous numerical values or summary statistics. For example, AGIS_test is a binary indicator that evaluates whether a predefined level of change from baseline has occurred consistently over time, whereas AGIS refers to the raw score itself at each visit. Similarly, MD_slope_test and VFI_slope_test are binary outcome variables defined according to progression criteria that incorporate statistical significance, whereas the predictor variables MD slope and VFI slope values represent the continuous magnitude of change itself. Therefore, we considered the structure of our study to be closer to the use of progression-related clinical measures in different forms, rather than a case of direct label leakage resulting from the reuse of identical variables.

In addition, the objective of this study was not to construct a completely independent prediction model after excluding all progression-related features. Rather, our aim was to investigate how different labeling strategies influence machine learning performance when clinically relevant progression descriptors are used as model inputs. Therefore, the inclusion of progression-related clinical variables as features was an intentional aspect of the study design.

Nevertheless, we agree that the possibility of circularity raised by the reviewer represents an important methodological consideration. To further evaluate this issue, we performed an additional ablation analysis (included in the Supplementary Materials) in which feature groups associated with individual progression criteria were removed one at a time, and model performance was re-evaluated. The results showed that the overall performance patterns were largely preserved despite the removal of specific feature groups, suggesting that the observed performance cannot be explained solely by a single progression criterion or simple one-to-one feature duplication.

Furthermore, not only the Consensus label but also the Wiggs’ label demonstrated correlations with progression-related variables, and several spatial variability-related features showed even stronger correlations with the Wiggs’ label. This suggests that the association between progression-related clinical descriptors and the labels is not unique to a particular labeling strategy but rather reflects a broader structural characteristic shared across different definitions of glaucoma progression. Therefore, we believe that the observed model performance is better explained by the integration of multiple progression-related patterns than by direct feature duplication alone. Accordingly, we have added a table reporting the correlations between each feature and both the Consensus and Wiggs’ labels as shown below as Supplementary Table 6.

Supplementary Table 6. Correlation between input features and progression labels (Consensus label and Wiggs’ label)

AGIS CIGTS MD MD slope VFI VFI slope TDV mean TDV SD PDV mean PDV SD

Consensus label 0.26 0.25 −0.32 −0.50 −0.29 −0.49 −0.31 0.22 −0.23 0.22

Wiggs’ label 0.26 0.18 −0.29 −0.34 −0.32 −0.37 −0.29 0.49 −0.31 0.49

We have added following sentences in the Results section.

"To further examine the relationship between input features and progression labels, and to address the potential concern that model performance under the Consensus label may partly reflect overlap between label-defining criteria and predictor variables, we calculated point-biserial correlation coefficients between each continuous input feature and both progression labels (Supplementary Table 6). Although the Consensus label showed stronger correlations with global trend-based features such as MD slope (r = −0.50) and VFI slope (r = −0.49), consistent with its algorithmic definition, the Wiggs' label demonstrated comparatively stronger associations with spatial variability features, particularly TDV standard deviation (r = 0.49) and PDV standard deviation (r = 0.49). This divergence in feature-label correlation patterns indicates that each label captures a distinct and internally coherent dimension of VF deterioration. Importantly, the performance differences observed between the two labeling strategies are therefore not solely attributable to direct overlap between input features and label-defining criteria, but rather reflect the degree to which each label is aligned with the overall feature space available in this study. The lower predictive performance observed under the Wiggs' label likely reflects the relative underrepresentation of spatially granular features in the current input set, rather than the clinical inferiority of the label itself."

3. The manuscript should clarify the train-test split in enough detail to rule out patient-level or eye-level leakage: The paper states that the unit of analysis is the eye-episode, that 9,514 eye-episodes from 4,496 patients were analyzed, and that 80% of eye-episodes were assigned to training and 20% to testing. However, it is not stated whether the split was grouped by patient or by eye. If multiple episodes from the same eye, or both eyes from the same patient, were allowed to appear in both training and test sets, the reported performance could be optimistic. The authors should clarify this explicitly and, preferably, provide a grouped patient-level split as a sensitivity analysis.

Response: We thank the reviewer for this important comment. As described in the original manuscript, 80% of all eye-episodes were used as the training dataset and the remaining 20% were used as the test dataset (“Of all eye-episodes, 80% were used as the training dataset and the remaining 20% were used as the test dataset.”). Because the present study was conducted at the eye-episode level, multiple eye-episodes originating from the same patient or eye could potentially be included in both the training and test datasets. This may introduce correlated information between the two datasets and could lead to optimistic estimates of model performance.

However, the primary objective of this study was not to develop a strictly patient-independent prediction model, but rather to compare how different labeling strategies influence the performance of machine learning models in learning longitudinal progression patterns. In addition, glaucomatous progression may manifest as heterogeneous longitudinal progression patterns over time, even within the same patient. Therefore, we considered eye-episode–level analysis to be appropriate for capturing a broader range of progression dynamics.

Nevertheless, to evaluate the potential impact raised by the reviewer, we performed an additional sensitivity analysis using a patient-level split, and the results have been provided in Supplementary Tables 7-9. After ensuring that all eye-episodes originating from the same patient were assigned exclusively to either the training or test dataset, the relative performance trends across labeling strategies and the main study conclusions remained consistent. These findings suggest that the primary observations of this study are not solely dependent on eye-episode–level data partitioning and that the comparative results of the proposed labeling strategies are relatively robust.

Supplementary Table 7. To prevent information leakage, train–test splitting was performed at the patient level using GroupShuffleSplit. All eye-episodes originating from the same patient, including both eyes and multiple episodes from the same eye, were assigned exclusively to either the training or test set.

Training data (n=7641) Test data

(n=1873)

Dataset characteristics Age (year), mean ± SD 56.738 ± 14.331 56.723 ± 14.555

The intervals between visual field examinations (year), mean ± SD 1.93 ± 0.54 1.938 ± 0.558

Consensus label: stable / progressing, n (%) 5365 (70.2%) / 2276 (29.8%) 1302 (69.5%) / 571 (30.5%)

Wiggs’ label: stable / progressing, n (%) 4846 (63.4%) / 2795 (36.6%) 1171 (62.5%) / 702 (37.5%)

Features used for Machine Learning AGIS, mean ± SD 0.181 ± 1.728 0.17 ± 1.782

CIGTS, mean ± SD 0.012 ± 2.552 -0.008 ± 2.57

MD, mean ± SD -0.148 ± 1.834 -0.173 ± 1.953

MD slope, mean ± SD -0.137 ± 0.422 -0.143 ± 0.426

VFI, mean ± SD -1.054 ± 5.293 -0.966 ± 5.697

VFI slope, mean ± SD -0.462 ± 1.445 -0.455 ± 1.461

Mean 52 TDV, mean ± SD -0.143 ± 1.877 -0.167 ± 1.972

Mean 52 PDV, mean ± SD -0.217 ± 1.147 -0.203 ± 1.186

Supplementary Table 8. Classification performance of machine learning models for detecting visual field progression using the Consensus label with patient-level train–test splitting

Accuracy

(95% CI)

Sensitivity

(95% CI) Specificity

(95% CI) Precision

(95% CI) F1 score

(95% CI)

SVM 0.939

(0.928-0.950) 0.809

(0.777-0.841) 0.996

(0.992-0.999) 0.989

(0.979-0.998) 0.890

(0.869-0.909)

RF 0.941

(0.929-0.952) 0.823

(0.792-0.855) 0.993

(0.988-0.997) 0.981

(0.968-0.993) 0.895

(0.875-0.913)

LR 0.935

(0.923-0.946) 0.792

(0.760-0.824) 0.998

(0.996-1.000) 0.996

(0.989-1.000) 0.882

(0.860-0.902)

XGBoost 0.937

(0.926-0.947) 0.813

(0.780-0.845) 0.992 (0.987-0.996) 0.977

(0.963-0.989) 0.887

(0.863-0.906)

Supplementary Table 9. Classification performance of machine learning models for detecting visual field progression using the Wiggs’ label with patient-level train–test splitting

Accuracy

(95% CI)

Sensitivity

(95% CI) Specificity

(95% CI) Precision

(95% CI) F1 score

(95% CI)

SVM 0.809

(0.791-0.826) 0.651

(0.618-0.688) 0.904

(0.886-0.921) 0.802

(0.770-0.835) 0.719

(0.691-0.747)

RF 0.805

(0.788-0.821) 0.687

(0.653-0.721) 0.875

(0.855-0.894) 0.768

(0.734-0.800) 0.725

(0.697-0.752)

LR 0.800

(0.784-0.818) 0.613

(0.575-0.649) 0.913

(0.897-0.929) 0.808

(0.772-0.840) 0.697

(0.669-0.724)

XGBoost 0.799

(0.780-0.816) 0.682

(0.648-0.715) 0.869

(0.850-0.889) 0.758

(0.726-0.793) 0.718

(0.692-0.744)

4. The multicenter nature of the dataset is a strength, but the current validation strategy does not fully support the manuscript’s generalizability claims: The manuscript emphasizes heterogeneous clinical settings and concludes that the proposed labeling strategy supports robust and generalizable AI systems. However, the Methods describe a single 80/20 split rather than center-held-out validation or a leave-one-center-out design. If data from the same institutions appear in both training and test sets, institution-specific patterns may be shared across splits. A center-level validation strategy would provide much stronger support for the generalizability claims, or alternatively the wording in the Abstract and Discussion should be moderated.

Response: We thank the reviewer for this important comment. we have moderated the wording in the Abstract to avoid overstating the generalizability of the proposed framework.

Specifically, the sentence: “These findings underscore that careful definition of ground truth is critical for developing robust and generalizable artificial intelligence systems for glaucoma.” has been revised to: “These findings underscore that careful definition of ground truth is critical for developing reliable artificial intelligence systems for glaucoma progression assessment.”

In addition, the Discussion statement: “Strict inclusion criteria regarding test intervals, reliability indices, and temporal continuity minimized noise-driven variability and enhanced the generalizability of the models.” has been revised to “Strict inclusion criteria regarding test intervals, reliability indices, and temporal continuity minimized noise-driven variability and improved data quality.”

These revisions more appropriately reflect the scope of the present study while avoiding claims of external generalizability that were not directly evaluated.

5. The reporting of the predictor set is internally inconsistent and should be corrected: The Methods state that the model uses 10 features, including the mean and standard deviation of TDV and PDV. However, Table 2 lists age, interval between examinations, AGIS, CIGTS, MD, MD slope, VFI, VFI slope, Mean 52 TDV, and Mean 52 PDV, but does not show the standard deviation of TDV or the standard deviation of PDV. In addition, age and interval are described in Table 2 as machine learning inputs, yet they are not included in the explicit list of 10 features in the Methods. The authors should reconcile the Methods and Table 2 so that the exact predictor set is unambiguous.

Response: We agree with the reviewer that the description may have caused confusion. The classifier input features consisted of the following 10 variables: AGIS, CIGTS, MD, MD slope, VFI, VFI slope, TDV mean, TDV standard deviation, PDV mean

Attachment Submitted filename: Response letter.docx

# Peer review: Decision Letter 1

**DOI:** 10.1371/journal.pone.0357179.r003

**Authors:** Suho Lim

12 Jun 2026

Dear Dr. Lee,

Thank you for submitting your manuscript to PLOS ONE. After careful consideration, we feel that it has merit but does not fully meet PLOS ONE’s publication criteria as it currently stands. Therefore, we invite you to submit a revised version of the manuscript that addresses the points raised during the review process.

Please submit your revised manuscript by Jul 27 2026 11:59PM. If you will need more time than this to complete your revisions, please reply to this message or contact the journal office at plosone@plos.org. When you're ready to submit your revision, log on to https://www.editorialmanager.com/pone/ and select the 'Submissions Needing Revision' folder to locate your manuscript file.

- A letter that responds to each point raised by the academic editor and reviewer(s). You should upload this letter as a separate file labeled 'Response to Reviewers'.
- A marked-up copy of your manuscript that highlights changes made to the original version. You should upload this as a separate file labeled 'Revised Manuscript with Track Changes'.
- An unmarked version of your revised paper without tracked changes. You should upload this as a separate file labeled 'Manuscript'.

If you would like to make changes to your financial disclosure, please include your updated statement in your cover letter. Guidelines for resubmitting your figure files are available below the reviewer comments at the end of this letter.

If applicable, we recommend that you deposit your laboratory protocols in protocols.io to enhance the reproducibility of your results. Protocols.io assigns your protocol its own identifier (DOI) so that it can be cited independently in the future. For instructions see: https://journals.plos.org/plosone/s/submission-guidelines#loc-laboratory-protocols. Additionally, PLOS ONE offers an option for publishing peer-reviewed Lab Protocol articles, which describe protocols hosted on protocols.io. Read more information on sharing protocols at https://plos.org/protocols?utm_medium=editorial-email&utm_source=authorletters&utm_campaign=protocols.

As the corresponding author, your ORCID iD is verified in the submission system and will appear in the published article. PLOS supports the use of ORCID, and we encourage all coauthors to register for an ORCID iD and use it as well. Please encourage your coauthors to verify their ORCID iD within the submission system before final acceptance, as unverified ORCID iDs will not appear in the published article. *Only*  the individual author can complete the verification step; PLOS staff *cannot*  verify ORCID iDs on behalf of authors.

We look forward to receiving your revised manuscript.

Kind regards,

Suho Lim

Academic Editor

PLOS One

**Journal Requirements:**

1. If the reviewer comments include a recommendation to cite specific previously published works, please review and evaluate these publications to determine whether they are relevant and should be cited. There is no requirement to cite these works unless the editor has indicated otherwise.

2. Please review your reference list to ensure that it is complete and correct. If you have cited papers that have been retracted, please include the rationale for doing so in the manuscript text, or remove these references and replace them with relevant current references. Any changes to the reference list should be mentioned in the rebuttal letter that accompanies your revised manuscript. If you need to cite a retracted article, indicate the article’s retracted status in the References list and also include a citation and full reference for the retraction notice.

[Note: HTML markup is below. Please do not edit.]

Reviewers' comments:

Reviewer's Responses to Questions

**Comments to the Author**

Reviewer #1: (No Response)

Reviewer #2: All comments have been addressed

**********

2. Is the manuscript technically sound, and do the data support the conclusions?

Reviewer #1: Partly

Reviewer #2: Yes

**********

3. Has the statistical analysis been performed appropriately and rigorously?

Reviewer #1: No

Reviewer #2: Yes

**********

4. Have the authors made all data underlying the findings in their manuscript fully available?

The PLOS Data policy

Reviewer #1: No

Reviewer #2: Yes

**********

5. Is the manuscript presented in an intelligible fashion and written in standard English?

Reviewer #1: Yes

Reviewer #2: Yes

**********

Reviewer #1: Comments to authors

Recommendation: Major revision

The revised manuscript has improved substantially, and I appreciate the authors’ detailed responses and additional analyses. In particular, the authors have clarified the retrospective classification nature of the study, added patient-level split sensitivity analysis, added leave-one-center-out validation, expanded the limitation regarding the absence of structural OCT validation, clarified the interpretation of the Wiggs’ label, and improved the reporting of preprocessing and implementation details. These changes address several important concerns raised in the previous review.

However, I do not recommend acceptance in the present form. Several important issues remain unresolved, particularly regarding predictor reporting, feature construction, data availability, reproducibility, and interpretation of the algorithm-derived labels. These issues should be addressed before the manuscript can be considered acceptable.

Major comments

1. The predictor set is still not reported consistently: The Methods state that 10 machine-learning input features were used, including the mean and standard deviation of TDV and PDV. However, Table 2 still reports only Mean 52 TDV and Mean 52 PDV and does not explicitly report TDV standard deviation and PDV standard deviation as separate input features. Since these variables appear to be part of the classifier input set, Table 2 should be revised to include all 10 input features clearly, or the Methods should be corrected if these variables were not included in the final models.

2. The feature construction procedure requires clearer description: The Methods state that each eye-episode was divided into an early phase and a late phase, that mean values of 10 features were calculated separately for the two phases, and that the late-minus-early difference was used as the classifier input. However, this description is not fully clear for slope-based variables such as MD slope and VFI slope, which are usually calculated as trend estimates over time rather than simple early/late mean values. The authors should define exactly how each of the 10 features was computed, particularly MD slope, VFI slope, TDV standard deviation, and PDV standard deviation, and clarify whether each feature represents a late-minus-early difference, a full-episode slope, or another summary measure.

3. The data availability statement remains inconsistent: The authors’ response explains that the complete raw multicenter clinical dataset cannot be publicly released because of ethical and institutional restrictions, while processed train/test data, sample data, and analysis code are available through GitHub. However, the submitted data availability information still indicates that all data are fully available without restriction. This discrepancy should be corrected. The final data availability statement should clearly distinguish restricted raw multicenter clinical data from publicly available processed datasets, sample data, and analysis code.

4. The public repository should be updated and aligned with the revised manuscript: The authors should ensure that the shared code, notebooks, and outputs reproduce the revised tables and analyses as presented in the manuscript. If some parts of the preprocessing workflow cannot be reproduced because the raw clinical data are restricted, this limitation should be stated clearly in both the repository and the manuscript. The repository should also clearly document which parts of the revised analysis can be reproduced from the shared materials, including bootstrap confidence intervals, patient-level split analysis, leave-one-center-out validation, and any hyperparameter-tuning sensitivity analysis. Any outputs in the public repository should also be updated to match the corrected values in the revised manuscript.

5. The concern about overlap between label construction and predictor variables remains only partially resolved: The added correlation and ablation analyses are useful and improve the manuscript. However, the primary models still use features that are closely aligned with the label-defining criteria. Therefore, the reported high performance should not be interpreted as evidence of clinical ground-truth validity. The manuscript should state more clearly that the results primarily reflect the learnability and internal consistency of algorithm-derived functional labels within the selected feature space.

6. The ground-truth terminology and interpretation remain too strong in places: Phrases such as “optimized ground truth,” “reliable ground truth,” and “true glaucomatous progression” remain stronger than the study design supports, given the absence of independent expert adjudication, structural OCT validation, or prospective clinical outcome validation. These phrases should be replaced with more precise wording such as “algorithm-derived functional reference label” or “functional progression label.”

7. The term “Consensus label” remains potentially misleading: The proposed label is defined as progression when at least one of five algorithms identifies progression. This is an inclusive any-positive composite rule rather than a consensus rule in the usual sense of agreement among methods. Because the term “Consensus label” may imply stronger agreement or clinical validity than the definition supports, the authors should either rename the label, for example as an “inclusive composite algorithmic label” or “any-positive algorithmic label,” or state more explicitly throughout the manuscript that the term refers to an inclusive composite definition rather than agreement among algorithms.

8. The manuscript should clarify whether performance comparisons are descriptive or inferential: The authors added confidence intervals for several performance metrics, which is helpful. However, the manuscript should clearly state whether AUC and average precision confidence intervals were calculated and where they are reported. In addition, if no formal statistical comparison between labeling strategies was performed, the comparison should be described as descriptive rather than inferential.

Minor comments

1. The Discussion still states that the models were compared with “only the labeling strategy varied.” This wording should be revised because changing the labeling strategy also changes positive class prevalence and likely the difficulty of the classification task. A more accurate statement would be that the same dataset, input feature set, and model architectures were used, while label definition, class balance, and classification difficulty differed between the two labeling approaches.

2. The authors added class balance across the training and test sets, which is helpful. However, my previous comment also requested center balance and disease severity balance across the split. These should either be reported, preferably in Table 2 or a supplementary table, or the authors should explain why they are not available.

3. The decision threshold or default prediction rule used to calculate sensitivity, specificity, precision, and F1 score should be stated explicitly in the Methods section, not only in the response letter.

4. The authors state that a fixed random seed was used, but the exact seed value should be reported in the Methods or Supplementary Materials to improve reproducibility.

5. The authors should ensure that the repository language is consistent with the revised manuscript. Any statements suggesting that the model or labeling strategy supports fully generalizable clinical AI systems should be moderated unless supported by external validation beyond the included centers.

6. The Methods and supplementary materials should clearly identify which analyses were part of the primary revision and which were sensitivity analyses. This is particularly important for the patient-level split, leave-one-center-out validation, bootstrap confidence intervals, and any hyperparameter-tuning analysis.

7. The preprocessing flow has improved, but the authors should ensure that Supplementary Figure 1 reports the number of visual field tests, eyes, patients, segments, and eye-episodes retained or excluded at each major filtering step, including the segmentation and five-visit episode construction steps.

Overall assessment

The revised manuscript is scientifically improved and addresses several of the original methodological concerns. The added patient-level and center-level validation analyses are valuable, and the overall framing is more appropriate than in the original submission. However, the remaining issues are not only editorial. The inconsistent predictor reporting, unclear feature construction, unresolved data availability discrepancy, repository-manuscript mismatch, incomplete reproducibility documentation, and remaining over strong ground-truth language require further correction. I therefore recommend major revision before the manuscript can be considered for acceptance.

Reviewer #2: (No Response)

**********

what does this mean?). If published, this will include your full peer review and any attached files.

If you choose “no”, your identity will remain anonymous but your review may still be made public.

**Do you want your identity to be public for this peer review?**  For information about this choice, including consent withdrawal, please see our Privacy Policy

Reviewer #1: No

Reviewer #2: **Yes:** Abdel-Razzak Al-Hinnawi

**********

[NOTE: If reviewer comments were submitted as an attachment file, they will be attached to this email and accessible via the submission site. Please log into your account, locate the manuscript record, and check for the action link "View Attachments". If this link does not appear, there are no attachment files.]

To ensure your figures meet our technical requirements, please review our figure guidelines: https://journals.plos.org/plosone/s/figures

You may also use PLOS’s free figure tool, NAAS, to help you prepare publication quality figures: https://journals.plos.org/plosone/s/figures#loc-tools-for-figure-preparation.

NAAS will assess whether your figures meet our technical requirements by comparing each figure against our figure specifications.

Attachment Submitted filename: Comments to authors.pdf

# Author comment: Author response to Decision Letter 2

**DOI:** 10.1371/journal.pone.0357179.r004

22 Jul 2026

Major comments

1. The predictor set is still not reported consistently: The Methods state that 10 machine-learning input features were used, including the mean and standard deviation of TDV and PDV. However, Table 2 still reports only Mean 52 TDV and Mean 52 PDV and does not explicitly report TDV standard deviation and PDV standard deviation as separate input features. Since these variables appear to be part of the classifier input set, Table 2 should be revised to include all 10 input features clearly, or the Methods should be corrected if these variables were not included in the final models.

Response: We thank the reviewer for this comment. To improve consistency between the Methods section and Table 2, we have revised Table 2 to explicitly list all 10 input features used in the model training. Specifically, the standard deviation variables for TDV and PDV, which were included in the classifier input set, are now listed as separate entries in the table.

Table 2. Demographic and visual field–derived features used as machine learning inputs in the training and test sets. Age and the interval between visual field examinations are reported as the mean and standard deviation for each eye-episode, whereas all other variables are presented as the mean and standard deviation of the change values, calculated as the difference between the late-phase and early-phase averages within each eye-episode.

Training data

(n = 7611) Test data

(n = 1903)

Dataset characteristics Age (year), mean ± SD 56.755 ± 14.484 56.656 ± 13.932

The intervals between visual field examinations (year), mean ± SD 1.933 ± 0.546 1.924 ± 0.536

Initial MD ≥ −6 dB 7536 1886

−6 dB > Initial MD ≥ −12 dB 68 16

−12 dB > Initial MD 7 1

Consensus label: stable / progressing, n (%) 5333 (70.1%) / 2278 (29.9%) 1334 (70.1%) / 569 (29.9%)

Wiggs’ label: stable / progressing, n (%) 4814 (63.3%) / 2797 (36.7%) 1203 (63.2%) / 700 (36.8%)

Center balance, n (%) Dong-A:3262 (42.9%),

Busan Paik : 979 (12.9%), Kosin : 236 (3.1%),

Pusan : 2627 (34.5%),

Yangsan : 507 (6.7%) Dong-A : 784 (41.2%),

Busan Paik : 247 (13.0%),

Kosin : 68 (3.6%),

Pusan : 666 (35.0%),

Yangsan : 138 (7.3%)

Features used for machine learning AGIS, mean ± SD 0.179 ± 1.744 0.176 ± 1.718

CIGTS, mean ± SD 0.012 ± 2.561 −0.006 ± 2.533

MD, mean ± SD −0.154 ± 1.87 −0.149 ± 1.81

MD slope, mean ± SD −0.14 ± 0.429 −0.129 ± 0.394

VFI, mean ± SD −1.026 ± 5.408 −1.08 ± 5.239

VFI slope, mean ± SD −0.469 ± 1.466 −0.427 ± 1.373

Mean 52 TDV mean, mean ± SD −0.149 ± 1.905 −0.143 ± 1.861

Mean 52 TDV SD, mean ± SD 2.52 ± 1.419 2.504 ± 1.382

Mean 52 PDV mean, mean ± SD −0.21 ± 1.16 −0.231 ± 1.133

Mean 52 PDV SD, mean ± SD 2.519 ± 1.419 2.503 ± 1.383

2. The feature construction procedure requires clearer description: The Methods state that each eye-episode was divided into an early phase and a late phase, that mean values of 10 features were calculated separately for the two phases, and that the late-minus-early difference was used as the classifier input. However, this description is not fully clear for slope-based variables such as MD slope and VFI slope, which are usually calculated as trend estimates over time rather than simple early/late mean values. The authors should define exactly how each of the 10 features was computed, particularly MD slope, VFI slope, TDV standard deviation, and PDV standard deviation, and clarify whether each feature represents a late-minus-early difference, a full-episode slope, or another summary measure.

Response: We revised the Methods section as follows.

(Original) For each eye-episode, the mean values of 10 features were calculated separately for the early and late phases. The difference between the late-phase means and the early-phase means (late minus early) was used as the feature value and served as the input to the classifier. The features used in this study were: the AGIS score, CIGTS score, MD, MD slope, VFI, VFI slope, and the mean and standard deviation of TDV and PDV for each eye-episode. Of all eye-episodes, 80% were used as the training dataset and the remaining 20% were used as the test dataset.

(Revised) For AGIS, CIGTS, MD, and VFI, the feature values were calculated as the differences between the late-phase and early-phase means. For TDV and PDV, the mean values for the early and late phases were first calculated at each of the 52 visual field locations. Pointwise changes were then obtained by subtracting the early-phase mean from the late-phase mean at each location. The mean and standard deviation of these pointwise changes across the 52 locations were calculated for each eye-episode. MD slope and VFI slope were calculated by linear regression using the five consecutive visual field examinations within each eye-episode, with time in years from the first examination in the episode as the independent variable. The features used in this study were the AGIS score, CIGTS score, MD, MD slope, VFI, VFI slope, and the mean and standard deviation of TDV and PDV for each eye-episode. Of all eye-episodes, 80% were used as the training dataset and the remaining 20% were used as the test dataset.

3. The data availability statement remains inconsistent: The authors’ response explains that the complete raw multicenter clinical dataset cannot be publicly released because of ethical and institutional restrictions, while processed train/test data, sample data, and analysis code are available through GitHub. However, the submitted data availability information still indicates that all data are fully available without restriction. This discrepancy should be corrected. The final data availability statement should clearly distinguish restricted raw multicenter clinical data from publicly available processed datasets, sample data, and analysis code.

Response: We thank the reviewer for identifying this discrepancy. The selection of “all data are fully available without restriction” during the initial submission resulted from our misunderstanding of the submission question. We interpreted this option to mean that all materials that could be ethically and legally shared would be made publicly available, rather than that the raw multicenter clinical data were available without restriction.

The raw multicenter clinical data contain protected patient information and therefore cannot be publicly released because of institutional and ethical restrictions. In contrast, the processed train/test datasets, de-identified sample data, and analysis code are publicly available through our GitHub repository.

We have revised the Data Availability Statement in the manuscript to clearly distinguish the restricted raw clinical data from the publicly available processed datasets, sample data, and analysis code.

In addition, we confirmed that the submission system does not allow authors to modify the originally selected Data Availability option after submission. Therefore, we have contacted the journal and requested that the submission record be corrected accordingly, and we are currently awaiting their response.

“The raw multicenter clinical data are not publicly available because of institutional and ethical restrictions protecting patient privacy. Processed train/test datasets, sample data, and analysis code used in this study are publicly available through the following GitHub repository:

https://github.com/kimhb1029/Visual_Field_Progression”

4. The public repository should be updated and aligned with the revised manuscript: The authors should ensure that the shared code, notebooks, and outputs reproduce the revised tables and analyses as presented in the manuscript. If some parts of the preprocessing workflow cannot be reproduced because the raw clinical data are restricted, this limitation should be stated clearly in both the repository and the manuscript. The repository should also clearly document which parts of the revised analysis can be reproduced from the shared materials, including bootstrap confidence intervals, patient-level split analysis, leave-one-center-out validation, and any hyperparameter-tuning sensitivity analysis. Any outputs in the public repository should also be updated to match the corrected values in the revised manuscript.

Response: We thank the reviewer for this important comment regarding the public repository and the reproducibility of the revised analyses. We acknowledge that the previously shared repository no longer reflected the current version of the manuscript because substantial revisions and additional analyses were introduced during the revision process.

To avoid confusion arising from inconsistencies between the repository and the revised manuscript, we temporarily removed the outdated repository contents while preparing the revised version. The repository has now been updated to align with the revised manuscript, including the revised code, notebooks, and outputs corresponding to the reported analyses.

Because the raw multicenter clinical data cannot be publicly shared owing to institutional and patient privacy restrictions, this limitation is explicitly stated in both the manuscript and the repository documentation. The repository also clearly specifies which analyses can be reproduced using the publicly available processed datasets, sample data, and analysis code, and which components require access to the original clinical dataset.

5. The concern about overlap between label construction and predictor variables remains only partially resolved: The added correlation and ablation analyses are useful and improve the manuscript. However, the primary models still use features that are closely aligned with the label-defining criteria. Therefore, the reported high performance should not be interpreted as evidence of clinical ground-truth validity. The manuscript should state more clearly that the results primarily reflect the learnability and internal consistency of algorithm-derived functional labels within the selected feature space.

Response: We agree with the reviewer that, because the predictor variables are closely related to the criteria used to construct the algorithm-derived labels, the high classification performance observed in this study should not be interpreted as evidence of the clinical validity of either label. Rather, the observed performance primarily reflects the relative learnability and internal consistency of the algorithm-derived functional progression labels within the selected feature space.

Accordingly, we added the following sentence to the Discussion:

The present results should not be interpreted as demonstrating the clinical validity of either label. Rather, they demonstrate the relative learnability and internal consistency of two algorithm-derived functional progression labels within the selected feature space.

6. The ground-truth terminology and interpretation remain too strong in places: Phrases such as “optimized ground truth,” “reliable ground truth,” and “true glaucomatous progression” remain stronger than the study design supports, given the absence of independent expert adjudication, structural OCT validation, or prospective clinical outcome validation. These phrases should be replaced with more precise wording such as “algorithm-derived functional reference label” or “functional progression label.”

Response: We thank the reviewer for this important observation. We agree that terms such as "optimized ground truth," "reliable ground truth," and "true glaucomatous progression" imply a degree of validation that our study design does not provide. Because these labels were generated algorithmically from functional (visual field) data, describing them as a definitive ground truth overstates their epistemic status.

Accordingly, we have revised the terminology throughout the manuscript. All instances of "optimized/reliable ground truth" and "true glaucomatous progression" have been replaced with "algorithm-derived functional reference label" and “glaucomatous progression”.

Response: We thank the reviewer for this valuable comment. As suggested, we have revised the terminology throughout the manuscript.

7. The term “Consensus label” remains potentially misleading: The proposed label is defined as progression when at least one of five algorithms identifies progression. This is an inclusive any-positive composite rule rather than a consensus rule in the usual sense of agreement among methods. Because the term “Consensus label” may imply stronger agreement or clinical validity than the definition supports, the authors should either rename the label, for example as an “inclusive composite algorithmic label” or “any-positive algorithmic label,” or state more explicitly throughout the manuscript that the term refers to an inclusive composite definition rather than agreement among algorithms.

Response: We thank the reviewer for this precise and important observation. We fully agree that the term "consensus" conventionally implies agreement among methods, and that our label, defined as progression detected by at least one of five algorithms, is more accurately characterized as an inclusive OR rule rather than a majority-agreement rule.

We are also aware that prior studies by Saeedi et al. and Sabharwal et al. defined their reference standards in the opposite direction, requiring agreement among four of six algorithms ("majority progression"), which more closely corresponds to a conventional consensus definition. We therefore cannot invoke these studies to justify our use of the term "Consensus label," and we appreciate the reviewer's implicit caution on this point.

Rather than renaming the label, however, we would like to offer a methodological and clinical justification for retaining the term with a clarified definition, as the reviewer indicated this would be acceptable.

The rationale for adopting an any-positive rule in the present study differs fundamentally from the rationale underlying majority-based definitions. Saeedi et al. and Sabharwal et al. aimed to construct a high-specificity reference standard by requiring agreement among multiple algorithms, thereby minimizing false-positive labels in the training set. Our study, by contrast, was designed to address a different problem: the systematic loss of genuine progression signals that occurs when any single algorithm, or a majority rule, is applied to a dataset where inter-algorithm agreement is known to be poor (κ = 0.12–0.52). Because each algorithm captures a distinct pattern of VF deterioration, requiring agreement risks discarding true progression events that are detectable only by a subset of methods. In the context of an irreversible disease where false negatives carry greater clinical consequence than false positives, an any-positive rule represents a deliberate and clinically motivated labeling strategy.

We acknowledge, however, that "Consensus label" may mislead readers into assuming majority agreement. To resolve this ambiguity without abandoning the term, which we believe captures the intent of integrating signals across heterogeneous algorithms, we have revised the manuscript to explicitly define the label at first use as follows in the Introduction section:

"Unlike majority-based consensus approaches that require agreement among multiple algorithms, this any-positive rule was adopted on clinical grounds: given the irreversibility of glaucomatous vision loss, the cost of a missed progression event was judged to outweigh the cost of a false-positive alert."

8. The manuscript should clarify whether performance comparisons are descriptive or inferential: The authors added confidence intervals for several performance metrics, which is helpful. However, the manuscript should clearly state whether AUC and average precision confidence intervals were calculated and where they are reported. In addition, if no formal statistical comparison between labeling strategies was performed, the comparison should be described as descriptive rather than inferential.

Response: We thank the reviewer for this helpful comment. We agree that the manuscript should clearly distinguish descriptive performance comparisons from formal statistical comparisons.

In the Methods section, we added the following sentence:

95% confidence int

Attachment Submitted filename: Response_letter_auresp_2.docx

# Editor report: Decision Letter 2

**DOI:** 10.1371/journal.pone.0357179.r005

**Authors:** Suho Lim

14 Aug 2026

Labeling Matters: A Multicenter Machine Learning Study on Visual Field Progression in Glaucoma

PONE-D-26-13861R2

Dear Dr. Lee,

We’re pleased to inform you that your manuscript has been judged scientifically suitable for publication and will be formally accepted for publication once it meets all outstanding technical requirements.

Within one week, you’ll receive an e-mail detailing the required amendments. When these have been addressed, you’ll receive a formal acceptance letter and your manuscript will be scheduled for publication.

An invoice will be generated when your article is formally accepted. Please note, if your institution has a publishing partnership with PLOS and your article meets the relevant criteria, all or part of your publication costs will be covered. Please make sure your user information is up-to-date by logging into Editorial Manager at Editorial Manager® and clicking the ‘Update My Information' link at the top of the page. For questions related to billing, please contact billing support.

If your institution or institutions have a press office, please notify them about your upcoming paper to help maximize its impact. If they’ll be preparing press materials, please inform our press team as soon as possible -- no later than 48 hours after receiving the formal acceptance. Your manuscript will remain under strict press embargo until 2 pm Eastern Time on the date of publication. For more information, please contact onepress@plos.org.

Kind regards,

Suho Lim

Academic Editor

PLOS One

Additional Editor Comments (optional):

Reviewers' comments:

# Editor report: Acceptance letter

**DOI:** 10.1371/journal.pone.0357179.r006

**Authors:** Suho Lim

PONE-D-26-13861R2

PLOS One

Dear Dr. Lee,

I'm pleased to inform you that your manuscript has been deemed suitable for publication in PLOS One. Congratulations! Your manuscript is now being handed over to our production team.

At this stage, our production department will prepare your paper for publication. This includes ensuring the following:

* All references, tables, and figures are properly cited

* All relevant supporting information is included in the manuscript submission,

* There are no issues that prevent the paper from being properly typeset

You will receive further instructions from the production team, including instructions on how to review your proof when it is ready. Please keep in mind that we are working through a large volume of accepted articles, so please give us a few days to review your paper and let you know the next and final steps.

Lastly, if your institution or institutions have a press office, please let them know about your upcoming paper now to help maximize its impact. If they'll be preparing press materials, please inform our press team within the next 48 hours. Your manuscript will remain under strict press embargo until 2 pm Eastern Time on the date of publication. For more information, please contact onepress@plos.org.

You will receive an invoice from PLOS for your publication fee after your manuscript has reached the completed accept phase. If you receive an email requesting payment before acceptance or for any other service, this may be a phishing scheme. Learn how to identify phishing emails and protect your accounts at https://explore.plos.org/phishing.

If we can help with anything else, please email us at customercare@plos.org.

Thank you for submitting your work to PLOS One and supporting open access.

Kind regards,

PLOS ONE Editorial Office Staff

on behalf of

Dr. Suho Lim

Academic Editor

PLOS One
